# Supplementary material for: The nature of small molecules adsorbed on defective carbon nanotubes
Source: R Soc Open Sci. 2019 Aug 14;6(8):190727. doi: 10.1098/rsos.190727 (PMC6731717; doi:10.1098/rsos.190727)
Supplement: Supplemental materials for the detailed calculation and analysis [file rsos190727supp1.docx]

Supplemental materials

**The nature of small molecules adsorbed on defective carbon nanotubes**

Danhui Li,^1^ ,2^,a^Fengting Wang,1, 2^,a^Zhiyuan Zhang,^1,2^ Wanrun Jiang,^1,2^ Yu Zhu,^1,2^ Zhigang Wang^1,2,*^  and Rui-Qin Zhang ^3, 4*^

*Institute of Atomic and Molecular Physics, Jilin University, Changchun 130012, China*

^2^*Jilin Provincial Key Laboratory of Applied Atomic and Molecular Spectroscopy (Jilin University), Changchun 130012, China*

3*Department of Physics, Centre for Functional Photonics (CFP), City University of Hong Kong, Hong Kong SAR, P. R. China*

*^4^ Beijing Computational Science Research Center, Beijing 100193, China*

**a***These authors contributed equally to this work*

^*^*Correspondence to:* [*wangzg@jlu.edu.cn (Z*](mailto:wangzg@jlu.edu.cn%20(Z)*. W.) or* [*aprqz@cityu.edu.hk (R*](mailto:aprqz@cityu.edu.hk%20(R)*. Z.)*

**Contents:**

**Part 1.** The coordinate system for chemical adsorption of “a” and “d” defective nanotubes adsorbed with NH_3_.

**Part 2.** The description of the stable structures for the physical adsorption case that “a”, “b”, “c”, “d” defective and perfect nanotubes adsorbed with CH_4_, H_2_O and NH_3_.

**Part 3.** The comparsion of the adsorption energies and charge transfer of small molecules (CH_4_, H_2_O, NH_3_) adsorbed on defective (“a”, “b”, “c”,“d”) and perfect nanotubes calclulated with DFT (PBE0-D3/6-31G(d,p)) and DFTB for the CA and PA case.

**Part 4.** The energies needed to create the four defect structures on SWCNT

**Part 5.** The comparison of the gap, charge transfer, adsorption energies of the D_5d_ and C_260_ fullerene adsorbed with CH_4_, H_2_O and NH_3_ molecules.

**Part 6.** The stable structure energies and adsorption energies comparison of the dissociative adsorption and coordination adsorption.

**Part 7.** The comparison of the local density of the states of the dissociative adsorption four cases: “a” and “d” defective nanotubes absorbed with the CH-CH_3_, CH-OH and CH-NH_2_ with the coordination adsorption.

**Part 8.** DFT Electronic density difference for adsorption structures.

**Part 9.** Electronic-based analysis of H_2_O, NH_3_ and CH_4_ adsorbed on the type “a” defected CNT.

**Part 10.** The coordinate data of the structures for “a”, “d” defective nanotubes adsorbed with small molecules for chemical adsorption for fig. 2 (a) in the main text.

**Part 11.** The coordinate date of the structures for “a”, “b”, “c”, “d” defective and perfect nanotubes adsorbed with small molecules for physical adsorption for fig. 2 (b) in the main text.

**Part 1.** The coordinate system for chemical adsorption of “a” and “d” defective nanotubes adsorbed with NH_3_.

**
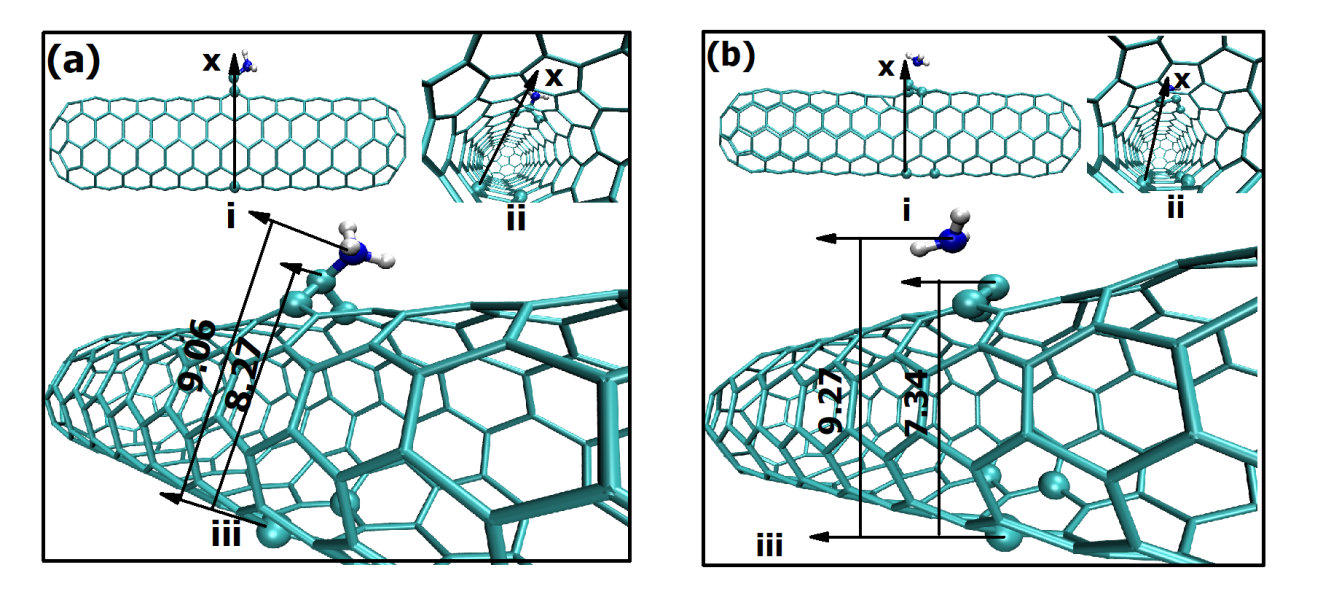
**

Figure S1. (a). The coordinate system of “a” defective nanotube adsorbed with NH_3_. (i) The front of the coodinate system. (ii) The side of the system. (iii) The coordinate of the top defective C-atom and N atom of the “a”defective nanotubes and NH_3_ respectively. (b). The coordinate system of “d” defective nanotube adsorbed with NH_3_. (i) The front of the coodinate system. (ii) The side of the system. (iii) The coordinate of the top defective C-atom and N atom of the “a”, “d” defective nanotubes and NH_3_ respectively.

**Part 2.** The description of the stable structures for the physical adsorption case that “a”, “b”, “c”, “d” defective and perfect nanotubes adsorbed with CH_4_, H_2_O and NH_3_.


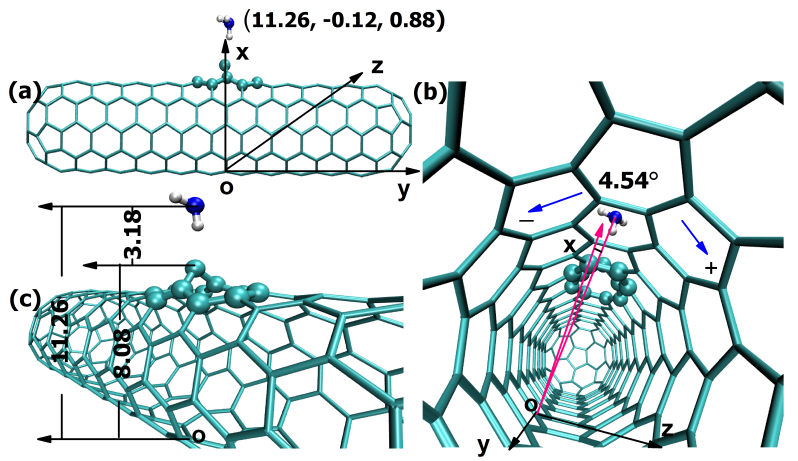


Figure S2. (a) The example of the established coordinate. (b) The angle of the line connecting the small molecules and origin C atom with the x axis. (c) The height of the defective and perfect nanotubes and the distance of small molecules to the bottom of the nanotubes.


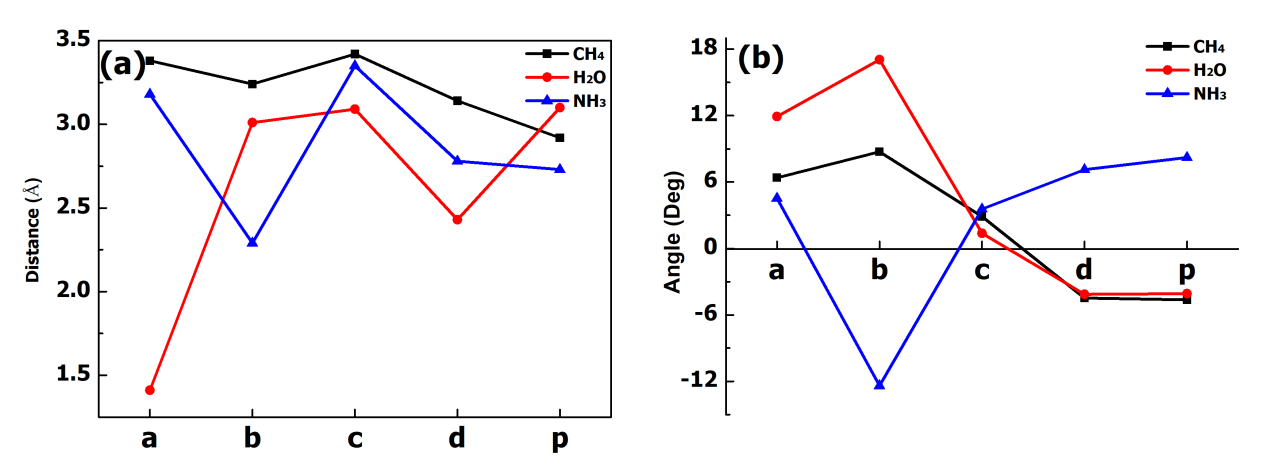


Figure S3. (a) The distance of the small molecules to the defective and perfect nanotubes of the stable structures for the PA case. (b) The angle of the line connecting the small molecules and origin C atom with the x axis.

Table S1. The dates of the distance of the height of the defective and perfect nanotubes (Distance 1) and the distance of small molecules to the bottom of the nanotubes (Distance 2) as well as the difference of them denoted as Dis2-Dis1 which show the distance of the small molecules to the defective or perfect nanotubes. The angles of the line connecting the small molecules and origin C atom with the x axis and the coordinate of the C, O, N atoms in the small molecules are also included.

| Nanotubes  Molecules | | | Coordinate | Angle | Distance 1 | Distance 2 | Dis 2-Dis 1 |
| --- | --- | --- | --- | --- | --- | --- | --- |
| CH_4_ | | “a” | (11.45, 0.31, 1.24) | 6.38 | 8.07 | 11.45 | 3.38 |
|  |  | “b” | ( 9.66,-0.04, 1.48) | 8.73 | 6.42 | 9.66 | 3.24 |
|  |  | “c” | (9.49, 0.07, 0.47) | 2.89 | 6.07 | 9.49 | 3.42 |
|  | | “d” | (10.18,0.03 , -0.81) | -4.48 | 7.04 | 10.18 | 3.14 |
|  |  | “p” | (9.62, 0.10, -0.77) | -4.63 | 6.70 | 9.62 | 2.92 |
|  | | “a” | (9.52, 1.73, 1.02) | 11.91 | 8.11 | 9.52 | 1.41 |
| H_2_O | | “b” | (9.25,-0.97, 2.72) | 17.05 | 6.24 | 9.25 | 3.01 |
|  |  | “c” | (9.17, 0.20, 0.08) | 1.38 | 6.08 | 9.17 | 3.09 |
|  |  | “d” | (9.51, 1.62, -0.15) | -4.13 | 7.08 | 9.51 | 2.43 |
|  | | “p” | (9.65, 0.19, -0.66) | -4.07 | 6.55 | 9.65 | 3.10 |
|  |  | “a” | (11.26, -0.12,0.88) | 4.54 | 8.08 | 11.26 | 3.18 |
|  |  | “b” | (8.56, -0.36, -2.00) | -12.39 | 6.27 | 8.56 | 2.29 |
| NH_3_ | PA | “c” | (9.42, -0.02, 0.59) | 3.56 | 6.07 | 9.42 | 3.35 |
|  |  | “d” | (9.83, 1.25, 0.14) | 7.13 | 7.05 | 9.83 | 2.78 |
|  |  | “p” | (9.44, 0.07, 1.36) | 8.22 | 6.71 | 9.44 | 2.73 |
|  |  | “a” | (9.06, 1.15, 1.01) | 9.58 | 8.27 | 9.06 | 0.79 |
|  | CA | “d” | (9.11, 0.81, 0.08 ) | 5.02 | 7.18 | 9.11 | 1.93 |

**Part 3.** The comparsion of the adsorption energies and charge transfer of small molecules (CH_4_, H_2_O, NH_3_) adsorbed on defective (“a”, “b”, “c”,“d”) and perfect nanotubes calclulated with DFT (PBE0-D3/6-31G(d,p)) and DFTB for the CA and PA case.

Table S2. The comparison of DFTB and DFT on the cases of PA and CA for “a” defective nanotube adsorbed with NH_3_.

| Tube |  | △E (meV) | | | Q (e) | |
| --- | --- | --- | --- | --- | --- | --- |
|  |  | DFTB | DFT |  | DFTB | DFT |
| a | PA | 78 | 175 |  | -0.01 | -0.02 |
|  | CA | 1260 | 1719 |  | 0.66 | 0.42 |

Table S3. The comparison of DFTB and DFT on the cases of PA and CA for “d” defective nanotube adsorbded with NH_3_.

| Tube |  | △E (meV) | |  | Q (e) | |
| --- | --- | --- | --- | --- | --- | --- |
|  |  | DFTB | DFT |  | DFTB | DFT |
| d | PA | 68 | 160 |  | 0 | 0.02 |
|  | CA | 461 | 679 |  | 0.27 | 0.24 |

**
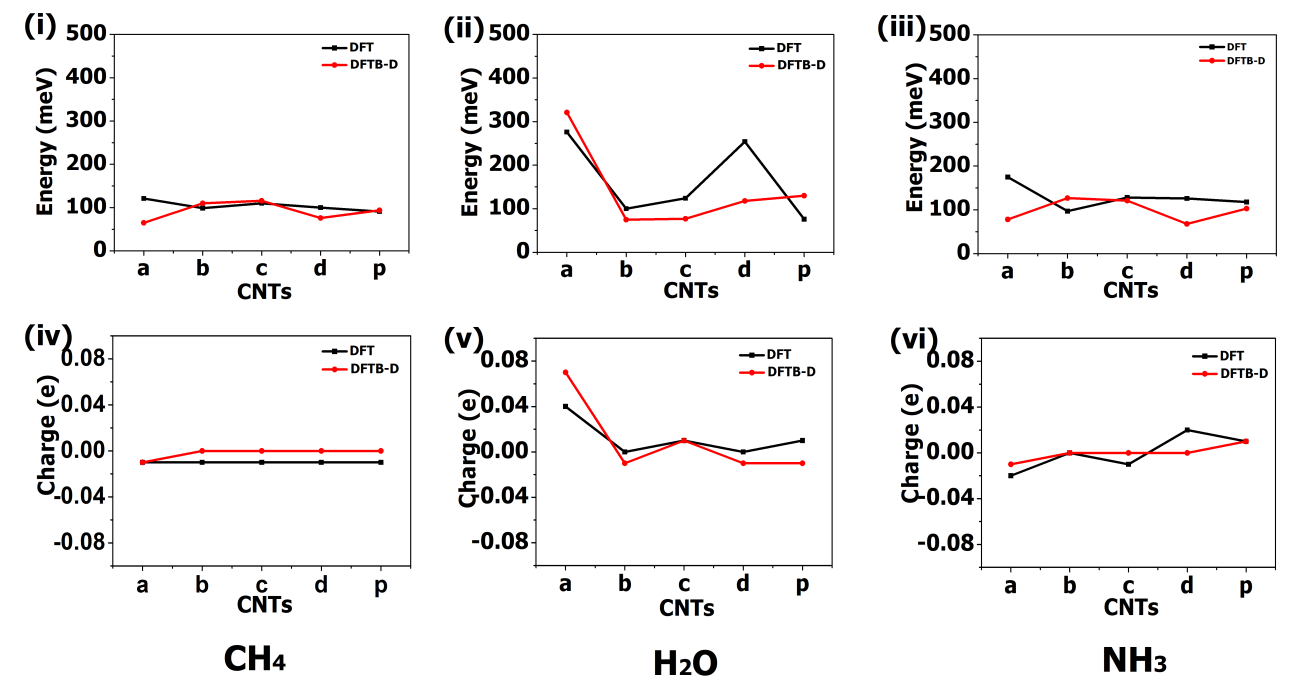
**

Figure S4. The comparsion of the adsorption energies and charge transfer of small molecules (CH_4_, H_2_O, NH_3_) adsorbed on defective (“a”, “b”, “c”,“d”) and perfect nanotubes calclulated with DFT (PBE0-D3/6-31G(d,p)) and DFTB for the PA case.

**Part 4.** The energies (eV) needed to create the four defective structures on SWCNT.

Table S4. The energies (eV) needed to create the four defect structures on SWCNT by DFTB-D. The positive values indicate that the formation is endothermic process.

| Defects | “a” | “b” | “c” | “d” |
| --- | --- | --- | --- | --- |
| Formation energies | 3.76 | 3.54 | 5.82 | 5.50 |

**Part 5.** The comparison of the gap, charge transfer, adsorption energies of the D_5d_ and C_260_ fullerene adsorbed with CH_4_, H_2_O and NH_3_ molecules.


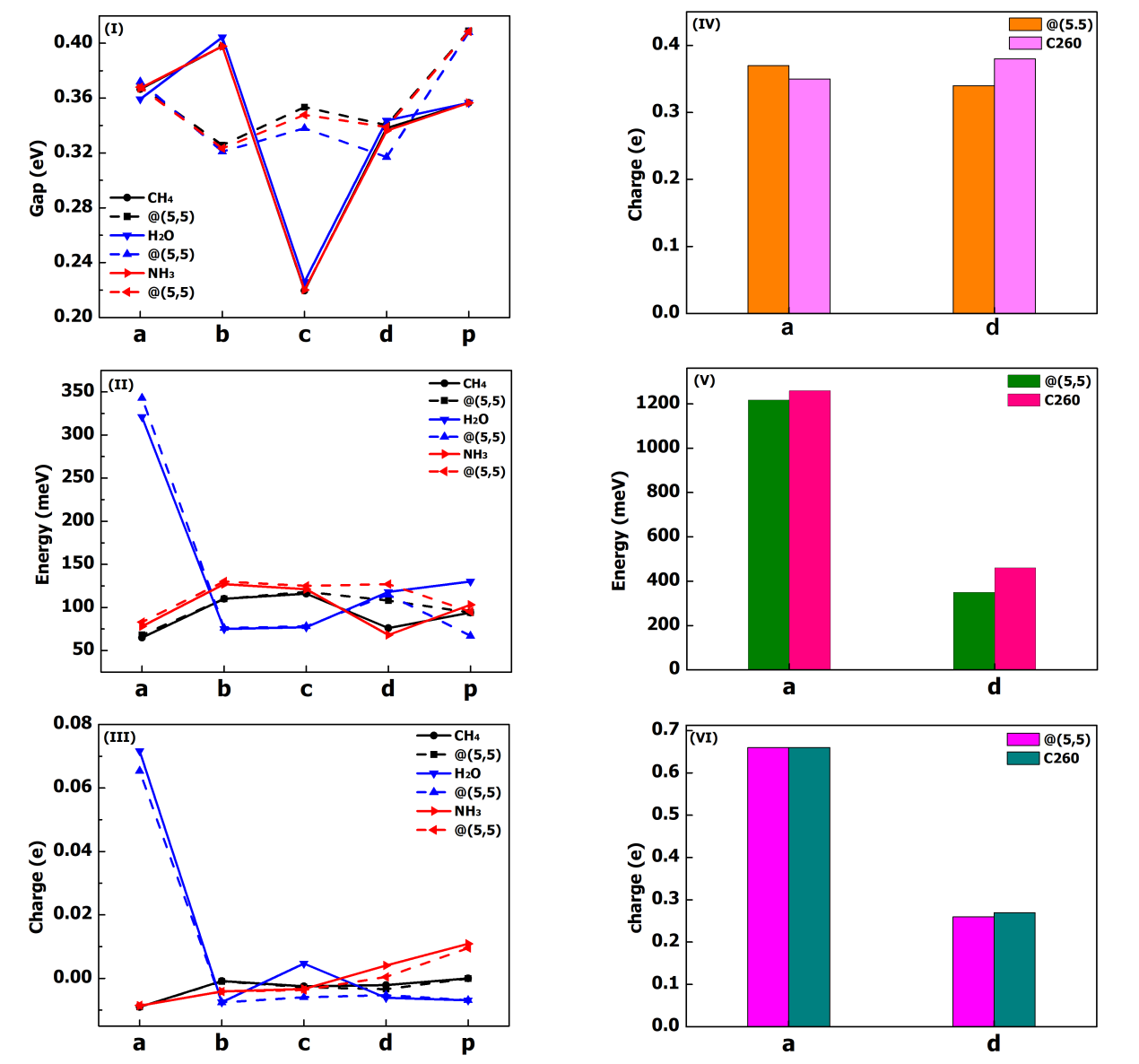


Figure S5. Comparison of the gap, adsorption energies, charge transfer results calculated for (5, 5) and C_260_ defective and perfect nanotubes adsorbed with CH_4_, H_2_O and NH_3_ on the physical and chemical case. (I) The gaps of the (5, 5) defective and perfect nanotubes (dotted line) and C_260_ fullerenes (solid line) for the PA case. (II) The adsorption energies of the (5, 5) defective and perfect nanotubes (dotted line) and C_260_ fullerenes (solid line) adsorbed with CH_4_, H_2_O, NH_3_ for the PA case. (III) The charge transfer of the (5, 5) defective and perfect nanotubes (dotted line) and C_260_ fullerenes (solid line) adsorbed with CH_4_, H_2_O, NH_3_ for the PA case. The positive values represent the charge transfer is from defective nanotubes to small molecules and the negative values represent the opposite direction. (IV) The gaps of the (5, 5) “a” and “d” defective nanotubes (dotted line) and C_260_ fullerenes (solid line) for the CA case. (V) The adsorption energies of the (5, 5) “a” and “d” defective nanotubes (dotted line) and C_260_ fullerenes (solid line) adsorbed with CH_4_, H_2_O, NH_3_ for the CA case. (VI) The charge transfer of the (5, 5) “a” and “d” defective nanotubes (dotted line) and C_260_ fullerenes (solid line) adsorbed with CH_4_, H_2_O, NH_3_ for the CA case.

Table S5. Comparison of the gap, adsorption energies, charge transfer values calculated for (5, 5) and C_260_ defective and perfect nanotubes adsorbed with CH_4_, H_2_O and NH_3_ on the chemical and physical case.

| Molecules | Defective  nanotubes | | D_5d_ C_260_  fullerene | | | (5, 5)  nanotube | | |
| --- | --- | --- | --- | --- | --- | --- | --- | --- |
|  |  |  | Gap | Charge  transfer | Adsorption  energies | Gap | Charge  transfer | Adsorption  energies |
|  | CA | “a” | 0.35 | 0.66 | 1260 | 0.37 | 0.66 | 1218 |
|  |  | “b” | 0.38 | 0.27 | 461 | 0.34 | 0.26 | 350 |
|  |  | “a” | 0.36 | -0.01 | 78 | 0.37 | -0.01 | 83 |
| NH_3_ |  | “b” | 0.40 | 0 | 127 | 0.32 | 0 | 130 |
|  | PA | “c” | 0.21 | 0 | 121 | 0.35 | 0 | 125 |
|  |  | “d” | 0.34 | 0 | 68 | 0.34 | 0 | 127 |
|  |  | “p” | 0.36 | 0.01 | 103 | 0.40 | 0.01 | 95 |
|  |  | “a” | 0.36 | 0.07 | 321 | 0.37 | 0.07 | 343 |
|  |  | “b” | 0.40 | -0.01 | 75 | 0.32 | -0.01 | 76 |
| H_2_O | PA | “c” | 0.22 | 0 | 77 | 0.34 | -0.01 | 78 |
|  |  | “d” | 0.34 | -0.01 | 118 | 0.32 | -0.01 | 115 |
|  |  | “p” | 0.36 | -0.01 | 130 | 0.41 | -0.01 | 67 |
|  |  | “a” | 0.37 | -0.01 | 65 | 0.37 | -0.01 | 68 |
|  |  | “b” | 0.40 | 0 | 110 | 0.33 | 0 | 110 |
| CH_4_ | PA | “c” | 0.22 | 0 | 116 | 0.35 | 0 | 118 |
|  |  | “d” | 0.34 | 0 | 76 | 0.34 | 0 | 108 |
|  |  | “p” | 0.36 | 0 | 94 | 0.41 | 0 | 94 |

**Part 6.** The stable structure energies and adsorption energies comparison of the dissociative adsorption and coordination adsorption.


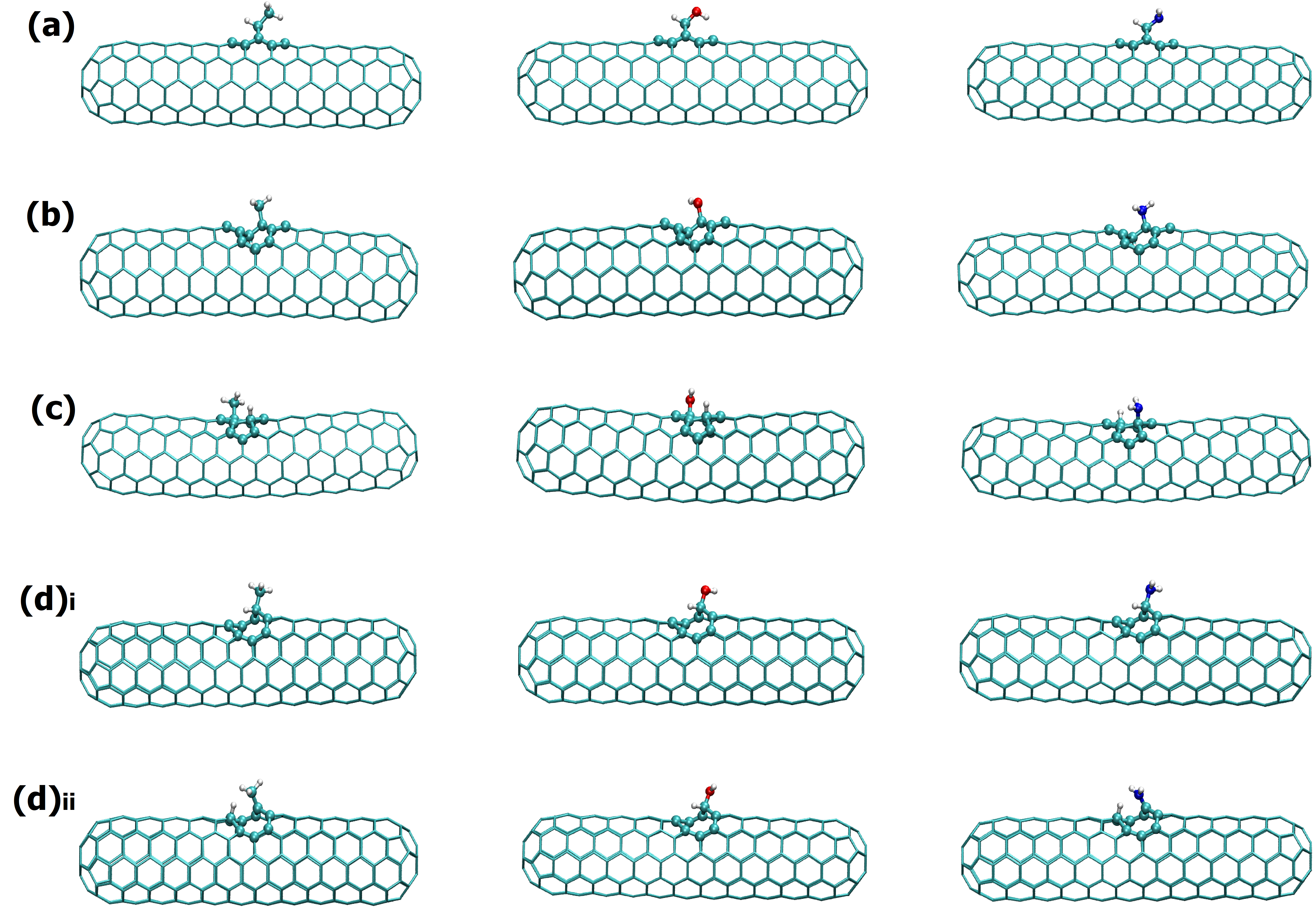


Figure S6. The stable structures of the dissociative adsorptions that “a”, “b”, “c”, “d” defective nanotubes adsorbed with CH_4_, H_2_O and NH_3_.

Table S6. The energies (Hartree) of the dissociative and corresponding coordination adsorptions stable structures.

| Energy (H) | CH_4_ | CH-CH_3_ | H_2_O | CH-OH | NH_3_ | CH-NH_2_ |
| --- | --- | --- | --- | --- | --- | --- |
| “a” | -454.10 | -454.22 | -454.96 | -455.06 | -454.41 | -454.49 |
| “b” | -452.39 | -452.38 | -453.24 | -453.21 | -452.66 | -452.64 |
| “c” | -448.85 | -448.83 | -449.69 | -449.67 | -449.12 | -449.09 |
| “d”(i) | -450.58 | -450.67 | -451.43 | -451.50 | -450.86 | -450.93 |
| “d”(ii) | -450.58 | -450.61 | -451.43 | -451.50 | -450.86 | -450.90 |


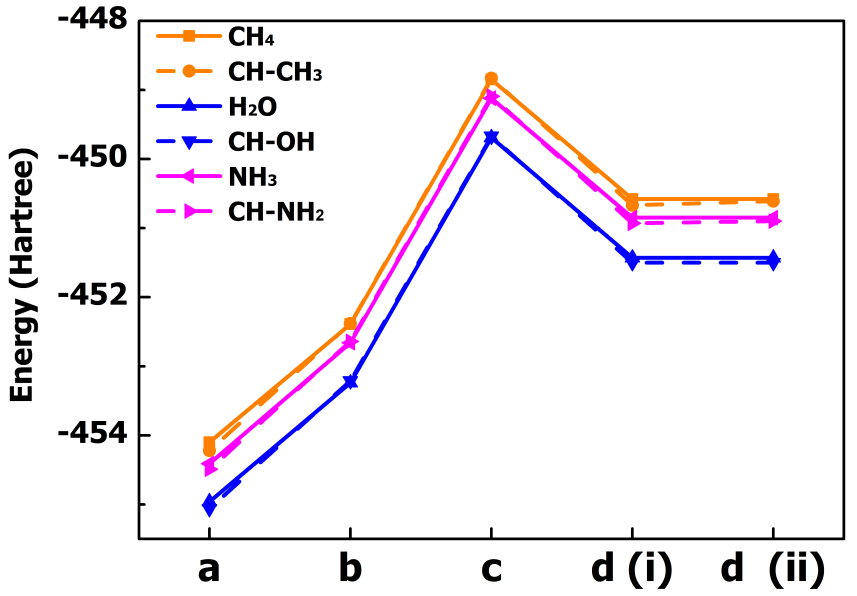


Figure S7. The comparison of the energies of the dissociative and corresponding coordination adsorptions.

Table S7. The adsorption energies (eV) of the possible dissociative adsorptions.

|  | CH-CH_3_ | | | CH-OH | | | CH-NH_2_ | | |
| --- | --- | --- | --- | --- | --- | --- | --- | --- | --- |
| Defects | “a” | “d”(i) | “d”(ii) | “a” | “d”(i) | “d”(ii) | “a” | “d”(i) | “d”(ii) |
| Energies | 3.33 | 2.52 | 0.89 | 3.04 | 2.02 | 2.02 | 3.44 | 2.36 | 1.55 |

**
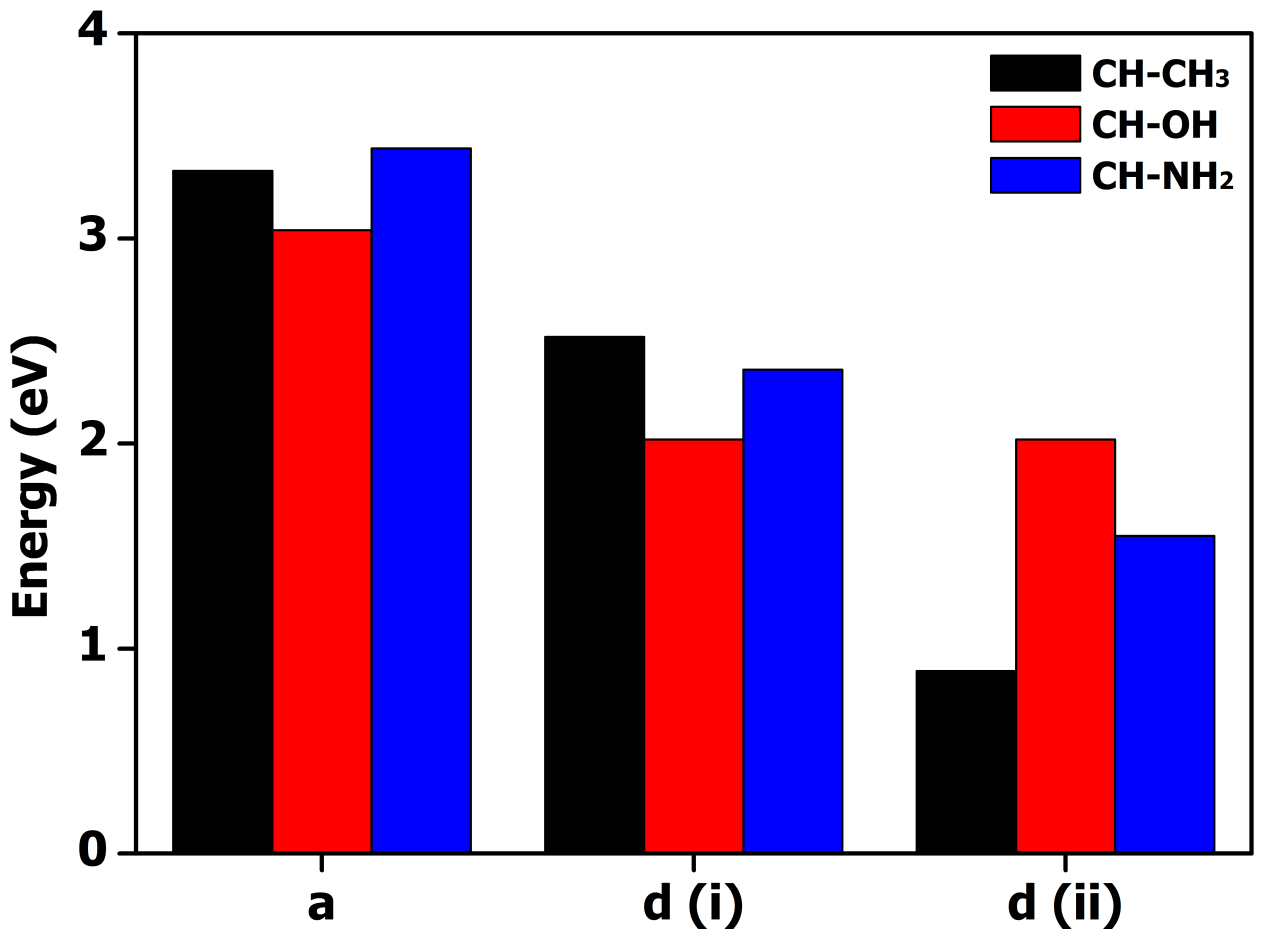
**

Figure S8. The columnar map of the adsorption energies about the possible dissociative adsorptions cases**.**

**Part 7.** The comparison of the local density of the states of the dissociative adsorption: “a” and “d” defective nanotubes adsorbed with the CH-CH_3_, CH-OH and CH-NH_2_ with the coordination adsorption.


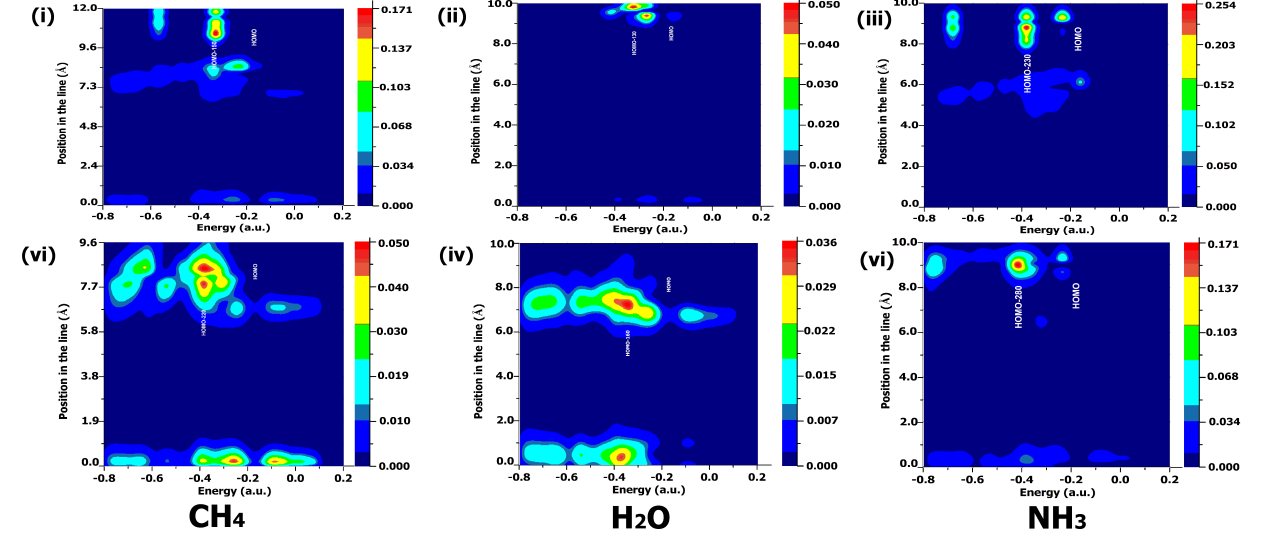


Figure S9. The comparison of the local density of the states about the possible dissociative adsorptions and corresponding coordination ones for “a” defective nanotube. (i) “a” adsorbed with CH_4_. (ii) “a” adsorbed with H_2_O. (iii) “a” adsorbed with NH_3_. (iv) “a” adsorbed with CH-CH_3._ (v) “a” adsorbed with CH-OH. (vi) “a” adsorbed with CH-NH_2_. The line connected the C atom which is located at the bottom of the defective nanotubes and N, O, C atoms in the small molecules. The highest occupied molecular orbital and the orbital which corresponds to the largest density are labeled. The HOMO is in the right and the others are HOMO-150, HOMO-130, HOMO-230, HOMO-220, HOMO-160, HOMO-280 respectively. We concluded that the distribution of the density of the states is more uniform in the dissociative adsorption than the coordination ones and the delocalization of the orbital is weaker.





Figure S10. The comparison of the local density of the states about the one of the possible dissociative adsorptions and corresponding coordination ones for “d” defective nanotube. (i). “d” adsorbed with CH_4_. (ii) “d” adsorbed with H_2_O. (iii) “d” adsorbed with NH_3_. (iv) “d” adsorbed with CH-CH_3._ (v) “d” adsorbed with CH-OH. (vi) “d” adsorbed with CH-NH_2_. The line connected the C atom which is located at the bottom of the defective nanotubes and N, O, C atoms in the small molecules. The highest occupied molecular orbital and the orbital which corresponds to the largest density are labeled. The HOMO is in the right and the others are HOMO-130, HOMO-80, HOMO-30, HOMO-160, HOMO-150, HOMO-270 respectively. We concluded that the distribution of the density of the states is more uniform in the dissociative adsorption than the coordination ones and the delocalization of the orbital is weaker.

**Part 8.** DFT Electronic density difference for adsorption structures.

For structures a and d in Figure S11 (a), the CA structures, the electronic density differences are still obvious, consistent with the large Milliken charge transfer for “ca” and “cd” shown in Figure 4 (b) in the manuscript. Whereas, for the PA structures shown in Figure S11 (b), it is obvious that the electronic density bubble is nearly negligible, except for H_2_O absorbed on type “a” defective CNT. Structure “a” shows a relatively larger electronic density difference among the pa, consistent with the Milliken charge transfer shown in Figure 4 (b) in the manuscript. This is possibly due to its hydrogen bonding like structure of O-H…C, indicating a higher polarization effect.





Figure S11. (a) The electronic density difference of NH_3_ adsorbed on the “a” and “d” defective nanotubes for the CA. (b) The electronic density difference of the small molecules (CH_4_, NH_3_, H_2_O) adsorbed on the “a”, “b”, “c” and “d” defective as well as perfect nanotubes for the PA. The isovalue for all the structures is set to 0.004. The dark blue density bubbles denote the electronic density increase, while the red ones electronic density decrease.

**Part 9.** Electronic-based analysis of H_2_O, NH_3_ and CH_4_ adsorbed on the type “a” defected CNT.





Fig. S12 The line electronic density of the H_2_O, NH_3_ and CH_4_ adsorbed on the type “a” CNT. The horizontal axis “relative intermolecular distances” means the geometrical position between the proton of adsorbates and the dangling C atom on the CNT. Due to the fact that the distances between the proton and dangling C atom are different, we normalized the distances into the same scales for comparison.





Fig. S13 The LDOS of the H_2_O (a), NH_3_ (b) and CH_4_ (c) adsorbed on the type “a” CNT. The horizontal axis is the energy scale of the orbitals, and the vertical axis means the positions from the dangling C atom in the defect sites to the proton. The dangling C atom locates on the origin.

**Part 10.** The coordinate data of the structures for “a”, “d” defective nanotubes adsorbed with small molecules for chemical adsorption for fig. 2 (a) in the main text.

Table S8. The coordinate of the structures for “a” defective nanotube adsorbed with NH_3_ for chemical adsorption.

C 3.59048424 -0.13964688 10.43099720

C 3.53144882 -0.11038995 5.48308823

C 3.47005704 0.60301721 6.72686044

C 3.55970137 -0.11986718 7.95768478

C 2.91791202 1.89860347 9.20547277

C 3.49175455 0.59065809 9.19580920

C 2.44892884 2.44733059 10.44917893

C 3.49958151 -0.09210314 0.55090526

C 3.43142442 0.62808981 1.78447971

C 3.49950974 -0.09445595 3.01908011

C 2.90391482 1.93819470 4.26349345

C 3.45489348 0.61840224 4.25545263

C 2.42980459 2.47108508 5.50345524

C 2.90634256 1.91192177 6.73672884

C 1.20686811 3.17938870 7.98315905

C 2.43843422 2.44894547 7.97686658

C 0.50093519 3.31909638 9.21785344

C 1.21672551 3.16796850 10.45543657

C 3.53950705 -0.07852374 -4.37968678

C 3.45794593 0.64389596 -3.15070573

C 3.49890244 -0.07794065 -1.91504380

C 2.89793497 1.95750643 -0.67407234

C 3.43679244 0.63575893 -0.67474507

C 2.43033604 2.50801913 0.55859871

C 2.89332501 1.94601050 1.79147434

C 1.20880995 3.23256630 3.03809412

C 2.42831908 2.49154847 3.03402797

C 0.50474257 3.38215695 4.27406887

C 1.20837566 3.20420186 5.50863020

C -0.91567983 3.19645450 6.74753581

C 0.50137687 3.34492454 6.74992658

C -1.57612836 2.88747859 7.97824338

C -0.91924641 3.16994829 9.21535078

C -2.64871223 1.91228968 10.43979666

C -1.59295820 2.87295745 10.45023607

C 3.58809981 -0.07299427 -9.32499436

C 3.50547397 0.64304660 -8.08942542

C 3.56453480 -0.07635140 -6.85535991

C 2.91715725 1.95085271 -5.61803700

C 3.47627496 0.64193760 -5.61576815

C 2.44445392 2.50779758 -4.38528728

C 2.90926757 1.96170830 -3.15036521

C 1.21429691 3.24685862 -1.91434850

C 2.42995779 2.50726640 -1.91298325

C 0.50791022 3.41250469 -0.67580827

C 1.21050534 3.25273017 0.55980880

C -0.91277908 3.25191074 1.79637728

C 0.50330179 3.39934182 1.79854230

C -1.57248809 2.94257772 3.03373762

C -0.91834563 3.23365444 4.27185619

C -2.61708507 1.94306560 5.49510785

C -1.57405153 2.91323762 5.50406320

C -2.97158100 1.29605949 6.72648245

C -2.62938790 1.91759666 7.96795275

C -3.27912044 -0.11954942 9.18348654

C -2.98886583 1.27887711 9.19470578

C -3.22913168 -0.85512079 10.41839888

C 2.96207739 1.96232073 -10.56599761

C 3.53821522 0.65627070 -10.56416217

C 2.45900815 2.49488089 -9.32817682

C 2.93270577 1.95486109 -8.09166213

C 1.22437256 3.22617967 -6.86403016

C 2.44828669 2.49580811 -6.86008039

C 0.51467388 3.38936299 -5.62656434

C 1.21966832 3.24563583 -4.38786230

C -0.90845973 3.25963664 -3.15770209

C 0.51200984 3.40745422 -3.15565673

C -1.56605585 2.95739521 -1.91840709

C -0.91255511 3.26471100 -0.67790766

C -2.61025369 1.98227471 0.55100456

C -1.57083041 2.96307978 0.55558285

C -2.95012305 1.33573402 1.78239757

C -2.61209085 1.96524201 3.02615228

C -3.23100824 -0.08119190 4.24443672

C -2.96642389 1.32424501 4.25392067

C -3.15884312 -0.81111824 5.47153088

C -3.25142327 -0.10126234 6.71510590

C -2.62150556 -2.14088113 7.94085957

C -3.19368090 -0.82800720 7.94546874

C -2.15049970 -2.68842500 9.17409612

C -2.65312067 -2.16131362 10.41377557

C 0.52238333 3.38817607 -10.57323591

C 1.22977776 3.21857657 -9.33233297

C -0.90082585 3.23118813 -8.10189656

C 0.52270000 3.38019104 -8.10006645

C -1.55833796 2.93527200 -6.86758479

C -0.90069380 3.24162672 -5.62852665

C -2.60971619 1.98003396 -4.39225912

C -1.56416524 2.95538295 -4.39169883

C -2.95457360 1.34923071 -3.15860250

C -2.60234611 1.98232944 -1.92033668

C -3.20159049 -0.05784435 -0.68459042

C -2.94971289 1.34705758 -0.68275486

C -3.11514839 -0.78334024 0.54076044

C -3.20198634 -0.06524959 1.77413495

C -2.54246920 -2.08661003 3.00256617

C -3.12211090 -0.78696523 3.00832601

C -2.10763319 -2.66108303 4.23367949

C -2.59180212 -2.11886881 5.46533306

C -0.91329824 -3.40719079 6.70916052

C -2.13390146 -2.67379417 6.70677257

C -0.21242132 -3.56927219 7.94475393

C -0.92026000 -3.41353908 9.17589318

C 1.20655288 -3.43847344 10.41812425

C -0.21291772 -3.58757157 10.41552004

C -0.89700197 3.23930395 -10.57493542

C -2.60899063 1.96393425 -9.33427257

C -1.55646770 2.92665785 -9.33560339

C -2.96335663 1.33744979 -8.09880519

C -2.60452502 1.96727235 -6.86659221

C -3.22829466 -0.05956956 -5.62447724

C -2.95332848 1.33691369 -5.62571453

C -3.14331819 -0.77753908 -4.38858833

C -3.21698788 -0.05386982 -3.16003694

C -2.52632673 -2.06104554 -1.92729913

C -3.11022287 -0.76864885 -1.92449015

C -2.07264257 -2.63144422 -0.69761650

C -2.55203429 -2.10155838 0.53707203

C -0.98460012 -3.56254882 1.76803443

C -2.08374913 -2.65185544 1.77017856

C -0.19883447 -3.65113747 2.99639229

C -0.89429078 -3.42219318 4.23011658

C 1.21164029 -3.42387003 5.47531349

C -0.20209110 -3.57185694 5.47281444

C 1.86855616 -3.11573085 6.71424683

C 1.21176447 -3.41986999 7.94732424

C 2.92013613 -2.15639063 9.18339576

C 1.86710560 -3.12099582 9.18109164

C 3.29779832 -1.53687603 10.42477976

C -3.28112358 -0.05841472 -10.57194027

C -2.98822718 1.33868987 -10.57283232

C -3.18106891 -0.78219515 -9.33282955

C -3.25148623 -0.06461448 -8.09753246

C -2.59516475 -2.08838375 -6.86610439

C -3.16267629 -0.78070154 -6.86360498

C -2.11208602 -2.63534265 -5.63031981

C -2.57281421 -2.08946761 -4.38903861

C -0.88039139 -3.40506867 -3.15848846

C -2.08520489 -2.63439546 -3.16227858

C -0.18728074 -3.64631910 -1.92017551

C -0.97969810 -3.54622100 -0.69987496

C 1.64444555 -3.85393952 0.53134331

C -0.52045016 -4.07967652 0.52777615

C 1.98862437 -3.25313142 1.77309516

C 1.23390637 -3.50192286 2.99880235

C 2.89191096 -2.13823036 4.24225574

C 1.86243448 -3.13408199 4.23489524

C 3.24833331 -1.50743288 5.47552330

C 2.91068003 -2.14521299 6.71589978

C 3.27219337 -1.52284951 7.95160213

C -2.13977055 -2.64547808 -10.57660880

C -2.60756466 -2.08834185 -9.33546731

C -0.89714494 -3.36279077 -8.10682257

C -2.12808731 -2.63254460 -8.10294263

C -0.19101968 -3.52453314 -6.87467509

C -0.89677527 -3.37380511 -5.63432258

C 1.22776862 -3.40988502 -4.39881797

C -0.19095647 -3.55796137 -4.40067945

C 1.86775409 -3.11853220 -3.15481508

C 1.23626599 -3.49817051 -1.91813408

C 2.86755460 -2.11608615 -0.69029461

C 1.98739475 -3.23703123 -0.69543780

C 3.22283135 -1.49866355 0.54600002

C 2.87543633 -2.13438418 1.77818612

C 3.20261444 -1.48630739 3.01196897

C -0.90755731 -3.36602269 -10.57980001

C 1.22788542 -3.35980945 -9.33983954

C -0.19058068 -3.50841713 -9.34147522

C 1.88650691 -3.07148330 -8.10363824

C 1.22659631 -3.37628030 -6.87296294

C 2.91916639 -2.10918058 -5.62401380

C 1.88303250 -3.08318885 -5.63088306

C 3.25389376 -1.48037898 -4.38139786

C 2.88702245 -2.11517004 -3.15554466

C 3.19642928 -1.46351647 -1.91911614

C 2.95820174 -2.11117950 -10.57090576

C 1.90211647 -3.07143351 -10.57673878

C 3.29800620 -1.46969866 -9.32870658

C 2.93938734 -2.10200808 -8.09697140

C 3.28085531 -1.47336811 -6.85896509

C -2.66694382 -2.12126610 -11.80677965

C -3.25207030 -0.79843418 -11.80421004

C -0.19275838 -3.56104036 -11.81154993

C 1.24575130 -3.41010346 -11.80992944

C 3.36757481 -1.48869158 -11.80017940

C 3.66558476 -0.07332950 -11.79651686

C 2.50680960 2.54488459 -11.79910070

C 1.25685514 3.27264796 -11.80325512

C -1.58923374 2.97417946 -11.80638819

C -2.66121051 2.00317181 -11.80500458

C -2.14073433 -2.63160342 -13.02046137

C -0.89842699 -3.35247353 -13.02298642

C 1.89524241 -3.05948293 -13.01997444

C 2.96098501 -2.09662852 -13.01491541

C 3.54249917 0.65158905 -13.00869364

C 2.96040120 1.96456062 -13.01022577

C -0.89767978 3.22414514 -13.01832029

C 0.53085125 3.37390153 -13.01672656

C -3.27959923 -0.06353488 -13.01624081

C -2.98238621 1.34164375 -13.01683202

C -0.21936572 -3.06615604 -14.26681931

C 1.17407734 -2.91994813 -14.26528278

C -2.80185275 -0.62013062 -14.26224046

C -2.23322166 -1.90039783 -14.26422203

C -2.31879009 1.65621693 -14.26236263

C -1.27822657 2.59438058 -14.26286507

C 1.03615534 2.83697173 -14.26034654

C 2.24859760 2.13498822 -14.25727977

C 2.90231384 -1.36190083 -14.25864070

C 3.19315883 0.00842148 -14.25567634

C -1.04289118 -2.16489718 -15.04578492

C -2.20207519 0.44197385 -15.04299816

C -0.08199625 2.35119752 -15.04217984

C 2.38781278 0.92319390 -15.03796057

C 1.79438034 -1.86728411 -15.04265778

C 0.04305731 1.16556592 -15.79390363

C 1.30002226 0.43942436 -15.79256645

C 0.99777501 -0.98032326 -15.79436409

C -0.44593648 -1.13174606 -15.79590632

C -1.03599333 0.19450247 -15.79510065

C -0.94819418 -3.47824624 11.64583419

C -2.19844925 -2.75042594 11.64455409

C 1.89843607 -3.17928944 11.65115216

C 2.97047011 -2.20784835 11.65414037

C 3.56106894 0.59353808 11.66789790

C 2.97559695 1.91644881 11.67739982

C 0.50147423 3.35530476 11.68877592

C -0.93714587 3.20423566 11.68615654

C -3.05887656 1.28321182 11.66606919

C -3.35735001 -0.13234688 11.65503664

C -0.22280659 -3.58686639 12.85824527

C 1.20694552 -3.43664335 12.86085480

C 3.29154961 -1.55358188 12.86910639

C 3.58894219 -0.14730952 12.87581648

C 2.44995087 2.42035573 12.89324208

C 1.20632921 3.14146845 12.89907130

C -2.65397188 1.88481191 12.88359805

C -1.58709503 2.84823405 12.89374543

C -2.65297856 -2.17742732 12.85795609

C -3.23571428 -0.86341425 12.86304013

C 1.58654574 -2.81305858 14.10800286

C 2.62718054 -1.87439316 14.11204838

C -1.94112527 -2.35387245 14.10352707

C -0.72829000 -3.05612489 14.10370143

C -2.88654798 -0.22703296 14.11290125

C -2.59552350 1.14368626 14.12295652

C -0.86671697 2.70195536 14.13799081

C 0.52710416 2.84821143 14.14058519

C 3.11067525 0.40221702 14.12414553

C 2.54161208 1.68272005 14.13263059

C 0.38977978 -2.57376885 14.88791683

C -2.08040292 -1.14578111 14.88998424

C -1.48717738 1.64476067 14.90938624

C 1.35039449 1.94241231 14.91469804

C 2.50975570 -0.66401813 14.89859836

C -0.69068371 0.75401740 15.65637661

C 0.75319121 0.90552602 15.65906005

C 1.34336405 -0.42070584 15.65134382

C 0.26436284 -1.39188331 15.64507379

C -0.99277913 -0.66582754 15.64696276

C 0.67072756 -5.00680090 0.43861200

H 0.84124960 -6.85136438 1.40850074

N 0.75728828 -5.85525062 1.67316137

H -0.08302087 -5.75629485 2.26657359

H 1.58047619 -5.61025104 2.24720274

Table S9. The coordinate of the structures for “d” defective nanotube adsorbed with NH_3_ for chemical adsorption.

C 5.19512677 -1.20957223 9.13899367

C 5.19565717 -1.23517264 4.18867650

C 5.15458424 -0.50526824 5.42850070

C 5.18878684 -1.21999965 6.66406933

C 4.61515384 0.83499270 7.88595221

C 5.13742723 -0.49216430 7.89464628

C 4.14852958 1.41728792 9.11385887

C 4.90713713 -1.18420664 -0.74879624

C 5.01920619 -0.50730643 0.48468425

C 5.17367670 -1.23044070 1.71845261

C 4.72786168 0.84752303 2.97166549

C 5.18784646 -0.51409853 2.95570588

C 4.22591387 1.40852402 4.18793633

C 4.65166312 0.82919698 5.42715930

C 2.96408401 2.15449934 6.61734938

C 4.17607624 1.39297578 6.64783105

C 2.24216092 2.32288731 7.83932622

C 2.93888502 2.17368317 9.08974526

C 4.92440026 -1.05149986 -5.61833671

C 4.73794795 -0.35583940 -4.38350760

C 4.77598782 -1.08638360 -3.15975100

C 3.93282230 0.86541325 -1.83162412

C 4.62228500 -0.39843294 -1.91082302

C 3.58169746 1.30935028 -0.49391165

C 4.47806378 0.79738245 0.50599414

C 3.05522529 2.15281876 1.67714751

C 4.26289772 1.41332267 1.76805768

C 2.33334374 2.33959348 2.88662408

C 3.02038591 2.16613470 4.14301057

C 0.86573483 2.17171067 5.34114570

C 2.28425578 2.31670989 5.36846412

C 0.18114189 1.90338543 6.57018459

C 0.82134217 2.20135085 7.81579602

C -0.95759280 1.01802765 9.04728928

C 0.12492154 1.94831066 9.05017961

C 5.12688306 -1.03579709 -10.56721174

C 5.01483927 -0.32127350 -9.33100844

C 5.04011770 -1.03942450 -8.09429117

C 4.36509017 0.98861611 -6.85434714

C 4.90666557 -0.32649825 -6.85211476

C 3.86051443 1.54529800 -5.62749445

C 4.18605396 0.95709091 -4.35859385

C 2.61838800 2.45125786 -3.23737350

C 3.64239465 1.49213994 -3.12438856

C 1.92785500 2.97349207 -2.06954389

C 1.07704336 1.87995245 0.34855226

C 2.50168039 2.00877686 0.33607456

C 0.35847479 1.72506229 1.60240468

C 0.92422407 2.11070040 2.85591010

C -0.82521891 0.89893872 4.09914305

C 0.23768593 1.84330812 4.08817009

C -1.24328521 0.31797126 5.34372996

C -0.89709585 0.96048736 6.57204912

C -1.64889636 -1.01563139 7.83020906

C -1.30013804 0.36820238 7.81057055

C -1.64137350 -1.72727676 9.07982582

C 4.51056712 0.99219737 -11.82222193

C 5.09636790 -0.30926622 -11.80956606

C 3.98573284 1.52247601 -10.59161796

C 4.44344255 0.99151355 -9.34192441

C 2.70818179 2.25474670 -8.16499642

C 3.93802338 1.53539289 -8.11764531

C 1.97256351 2.42799707 -6.94953944

C 2.66747368 2.32692627 -5.70256171

C 0.52991398 2.32998887 -4.51030846

C 1.94501898 2.56960676 -4.50587398

C -0.04578223 1.92152236 -3.25543167

C 0.65476856 2.25231612 -2.05633326

C -0.81956100 0.76912497 -0.80839314

C 0.25958605 1.69236544 -0.83464018

C -1.17017706 0.17000285 0.43134426

C -0.75198008 0.81863127 1.63516688

C -1.59496000 -1.11346906 2.89821261

C -1.18819026 0.25985846 2.87289960

C -1.56960095 -1.81029254 4.14529345

C -1.60846611 -1.05942092 5.36623977

C -1.08296826 -3.09516371 6.63710704

C -1.59470306 -1.75839959 6.61141330

C -0.62817388 -3.62777448 7.88164253

C -1.11588899 -3.05406156 9.10595856

C 2.05208333 2.38124613 -11.87398528

C 2.74731463 2.22863465 -10.62524733

C 0.59877505 2.20397190 -9.42688877

C 2.01855706 2.38451692 -9.40787208

C -0.06852720 1.89651521 -8.20184247

C 0.56424527 2.24336060 -6.96382044

C -1.07779161 0.89456759 -5.72322131

C -0.09666507 1.94205199 -5.72698790

C -1.37547618 0.23798175 -4.49056499

C -0.99271579 0.85816859 -3.25506656

C -1.65352545 -1.18239491 -2.02053708

C -1.29348112 0.20176169 -2.02510662

C -1.60250660 -1.90469098 -0.79016568

C -1.60711264 -1.17539394 0.44441811

C -1.09212443 -3.21248750 1.70302149

C -1.58531509 -1.87417058 1.68998072

C -0.62856102 -3.74326941 2.94508718

C -1.07701860 -3.14562886 4.16789853

C 0.59274881 -4.42915664 5.43276395

C -0.61811513 -3.67833396 5.41768483

C 1.29069923 -4.57379711 6.67223483

C 0.58271419 -4.38255201 7.89955941

C 2.70277302 -4.43239199 9.15119659

C 1.28034448 -4.54849320 9.14562714

C 0.63584752 2.20644337 -11.89415274

C -1.06990805 0.89889514 -10.67075369

C -0.03451146 1.87969198 -10.66472948

C -1.42285056 0.26384353 -9.43717469

C -1.08394091 0.89631045 -8.20160641

C -1.67911680 -1.14655021 -6.96208086

C -1.41147847 0.25271980 -6.95793346

C -1.61085337 -1.87327195 -5.72933197

C -1.66384626 -1.16153183 -4.49238293

C -1.08011221 -3.21993938 -3.25530906

C -1.60499809 -1.89536454 -3.25933312

C -0.62676820 -3.78784987 -2.01513427

C -1.09377003 -3.23745215 -0.77975788

C 0.58930087 -4.51634175 0.47875259

C -0.62879532 -3.78014315 0.46693625

C 1.29682197 -4.65791024 1.72344107

C 0.59313972 -4.48642554 2.95695224

C 2.71644330 -4.46184116 4.19926974

C 1.30057818 -4.61256528 4.19655826

C 3.37730103 -4.16020396 5.43885769

C 2.71640638 -4.44229789 6.67600968

C 4.45468641 -3.21315486 7.90694118

C 3.37415824 -4.14630035 7.91081053

C 4.85029525 -2.59447008 9.14567717

C -1.70207481 -1.12908207 -11.91940491

C -1.42563582 0.27129491 -11.91488481

C -1.60916658 -1.85419932 -10.67869899

C -1.69465795 -1.14249997 -9.44097133

C -1.04830165 -3.17076970 -8.20860965

C -1.61057952 -1.86102083 -8.20612109

C -0.58570445 -3.73007728 -6.96879681

C -1.06898859 -3.19783202 -5.73064270

C 0.60478838 -4.51132591 -4.49038869

C -0.61106533 -3.76303479 -4.49756237

C 1.30005322 -4.68161328 -3.24411086

C 0.59105905 -4.53585410 -2.00355272

C 2.71257377 -4.53313749 -0.75034344

C 1.29225304 -4.68925620 -0.76101162

C 3.37064910 -4.21104765 0.48635891

C 2.71647528 -4.49945778 1.72954243

C 4.44828093 -3.23328111 2.95683925

C 3.37804799 -4.18547779 2.95997239

C 4.82721886 -2.61191793 4.19116146

C 4.45028394 -3.22255040 5.43493342

C 4.83548318 -2.60841021 6.66911966

C -0.53833695 -3.70487947 -11.91749135

C -1.02945959 -3.15845733 -10.67995246

C 0.67188984 -4.42706221 -9.43521012

C -0.56522185 -3.70671115 -9.44369115

C 1.36140356 -4.58868453 -8.19409593

C 0.63418205 -4.46390431 -6.96234210

C 2.74257421 -4.46648673 -5.70388080

C 1.32388986 -4.64590549 -5.72024249

C 3.37497425 -4.17044657 -4.45969578

C 2.71273082 -4.51764509 -3.23362088

C 4.35385326 -3.20829089 -1.98116691

C 3.35967939 -4.22633909 -1.98884172

C 4.71306605 -2.59369795 -0.75401803

C 4.41352192 -3.23960905 0.48797756

C 4.80724260 -2.61404796 1.71930405

C 0.70223181 -4.41033167 -11.90653181

C 2.81625452 -4.37086394 -10.63490591

C 1.40111204 -4.55000766 -10.65841944

C 3.44651466 -4.07091710 -9.38870938

C 2.77426954 -4.40492854 -8.17323500

C 4.40720298 -3.09557018 -6.89432321

C 3.40485094 -4.10558080 -6.92159148

C 4.70272056 -2.46367465 -5.64660149

C 4.36114317 -3.13823374 -4.43834344

C 4.64324547 -2.50074346 -3.19135448

C 4.54340058 -3.08291168 -11.83037801

C 3.50537696 -4.06244971 -11.85737921

C 4.84753638 -2.43512788 -10.58193136

C 4.47383782 -3.07435647 -9.36006366

C 4.77385840 -2.43993085 -8.11498563

C -1.05114787 -3.18128538 -13.15412213

C -1.65026710 -1.86458925 -13.15429999

C 1.44091712 -4.58800565 -13.12671014

C 2.87648488 -4.41245964 -13.10134976

C 4.96770238 -2.45640486 -13.05182468

C 5.24972161 -1.03776693 -13.04087584

C 4.06563131 1.56674987 -13.06431423

C 2.80587206 2.27694305 -13.09170555

C -0.03646849 1.93132412 -13.13270765

C -1.09335292 0.94364694 -13.14273759

C -0.49962877 -3.68052308 -14.36119645

C 0.75184061 -4.38622207 -14.34819607

C 3.54058238 -4.04931827 -14.29869742

C 4.59226674 -3.07090494 -14.27354530

C 5.13835629 -0.31574266 -14.25613585

C 4.54241182 0.99090427 -14.26857008

C 0.66738212 2.19508692 -14.33468843

C 2.09367539 2.36561560 -14.31381020

C -1.66978888 -1.12752045 -14.36571068

C -1.38932072 0.28101352 -14.35972092

C 1.44697351 -4.08577758 -15.57948992

C 2.83830606 -3.91968707 -15.55564671

C -1.16742158 -1.67445462 -15.60644430

C -0.58245416 -2.94718705 -15.60435844

C -0.71386136 0.60766158 -15.59632019

C 0.31322003 1.56057981 -15.58383593

C 2.62410708 1.83636581 -15.54967658

C 3.84587644 1.15091745 -15.52594330

C 4.54430897 -2.33699440 -15.51772716

C 4.81708142 -0.96311473 -15.50886242

C 0.62296154 -3.19376118 -16.36856809

C -0.57004046 -0.60318354 -16.37686090

C 1.52370047 1.33536502 -16.34694044

C 4.01210672 -0.05921140 -16.30389088

C 3.45650297 -2.85709522 -16.32075726

C 1.67570761 0.15175456 -17.09615606

C 2.94249614 -0.55682746 -17.07550356

C 2.65936723 -1.98041021 -17.08363961

C 1.21786154 -2.15146549 -17.10751444

C 0.60982719 -0.83353557 -17.11315833

C 0.54114038 -4.39685926 10.36971655

C -0.68676802 -3.63238687 10.34950677

C 3.39372736 -4.17466917 10.38578411

C 4.49474084 -3.23631754 10.38220210

C 5.18290760 -0.45699286 10.36469898

C 4.64424259 0.88565379 10.35235868

C 2.21516381 2.39936590 10.31028644

C 0.77326228 2.28669514 10.28927484

C -1.40413901 0.42915365 10.28147831

C -1.75235859 -0.97482668 10.29863337

C 1.25548079 -4.50231025 11.58871152

C 2.68810991 -4.39194596 11.59596390

C 4.82962493 -2.57525800 11.59119495

C 5.17343026 -1.18055067 11.58241439

C 4.12136111 1.42491975 11.55382578

C 2.90061804 2.18229219 11.53214653

C -0.99329453 1.04037156 11.49242866

C 0.10082875 1.97104251 11.49664992

C -1.12934957 -3.01851760 11.54715930

C -1.66810751 -1.68657899 11.52167963

C 3.07827358 -3.76071582 12.83681500

C 4.14704991 -2.85536520 12.83436007

C -0.43273719 -3.19459359 12.80134175

C 0.75781242 -3.93359300 12.82106090

C -1.30814047 -1.03810536 12.76195196

C -0.97285162 0.32204618 12.74735701

C 0.80266948 1.82586680 12.75262372

C 2.19959414 1.93006255 12.77084529

C 4.70362759 -0.59585137 12.81887233

C 4.17663390 0.70241203 12.80419899

C 1.88475961 -3.47200051 13.60478854

C -0.53965512 -1.96806627 13.56382842

C 0.14216621 0.80246677 13.53638462

C 2.98640173 1.01249570 13.56896730

C 4.06258446 -1.63042077 13.60294103

C 0.90262639 -0.09937717 14.30798482

C 2.35029838 0.00648227 14.32316784

C 2.89867210 -1.33759952 14.34194870

C 1.78996881 -2.27441093 14.34181245

C 0.55647360 -1.50907757 14.32099851

H 0.65874569 4.62176968 -3.61208863

N 1.28534569 4.80763856 -2.82624772

H 0.80920743 5.25939251 -2.04329889

H 2.13204038 5.30491664 -3.10770537

**Part 11.** The coordinate date of the structures for “a”, “b”, “c”, “d” defective and perfect nanotubes adsorbed with small molecules for physical adsorption for fig. 2 (b) in the main text.

Table S10. The coordinate of the structures for “a” defective nanotube adsorbed with CH_4_ for physical adsorption.

C 3.57775223 -0.22549552 10.43783416

C 3.50980452 -0.21111445 5.49047265

C 3.45187262 0.50609202 6.73209586

C 3.54220779 -0.21356619 7.96436799

C 2.90521968 1.80927516 9.20683810

C 3.47713208 0.50079767 9.20038307

C 2.43887675 2.36291008 10.44946730

C 3.48625029 -0.20143365 0.55869012

C 3.41529728 0.52086940 1.79121134

C 3.48283419 -0.19900971 3.02798887

C 2.88742671 1.83686337 4.26562440

C 3.43730051 0.51657923 4.26177048

C 2.41388880 2.37291023 5.50424584

C 2.89116133 1.81624721 6.73855290

C 1.19466466 3.08910250 7.98241183

C 2.42542808 2.35744421 7.97731086

C 0.49043284 3.23275585 9.21756625

C 1.20777520 3.08515350 10.45492443

C 3.51715758 -0.20479399 -4.37176320

C 3.43797395 0.52274374 -3.14583609

C 3.48485573 -0.19445213 -1.90803885

C 2.88044093 1.84283061 -0.67246134

C 3.42374456 0.52225317 -0.66971798

C 2.41183139 2.39530233 0.55890725

C 2.87584087 1.83772741 1.79391583

C 1.19167335 3.12796649 3.03742530

C 2.41149597 2.38723585 3.03499613

C 0.48861218 3.28139123 4.27333815

C 1.19327706 3.10670679 5.50800084

C -0.92926918 3.10424882 6.74907174

C 0.48777953 3.25173717 6.74958713

C -1.58829634 2.79946577 7.98146412

C -0.92953966 3.08495685 9.21703181

C -2.65813540 1.83271431 10.44759955

C -1.60195464 2.79280629 10.45391910

C 3.56214182 -0.21734329 -9.31535621

C 3.48088384 0.50346985 -8.08299054

C 3.53948560 -0.21146535 -6.84638306

C 2.89463765 1.82081100 -5.61595206

C 3.45354225 0.51139774 -5.60964352

C 2.42229048 2.38145261 -4.38516807

C 2.88855594 1.83970855 -3.14885165

C 1.19322153 3.12571089 -1.91526751

C 2.41013735 2.38792716 -1.91238739

C 0.48780574 3.29442968 -0.67693853

C 1.19132808 3.13855415 0.55863198

C -0.93089080 3.14191350 1.79722655

C 0.48503150 3.28926999 1.79772275

C -1.59006292 2.83848234 3.03642011

C -0.93450749 3.13328088 4.27283014

C -2.63240075 1.84787756 5.50245875

C -1.58892682 2.81715540 5.50701723

C -2.98580529 1.20484602 6.73646348

C -2.64218083 1.83021859 7.97547154

C -3.29043561 -0.20307059 9.19796544

C -2.99979382 1.19505027 9.20474219

C -3.24037687 -0.93455843 10.43544668

C 2.93679549 1.81330259 -10.56318212

C 3.51253751 0.50704665 -10.55709086

C 2.43438922 2.35093220 -9.32738789

C 2.90867379 1.81567157 -8.08945366

C 1.19983289 3.08985195 -6.86498965

C 2.42444599 2.36079100 -6.85949141

C 0.49088270 3.25727683 -5.62775030

C 1.19641938 3.11778123 -4.38919862

C -0.93052536 3.13397443 -3.15714798

C 0.48978269 3.28182627 -3.15664292

C -1.58761177 2.83626893 -1.91626328

C -0.93293596 3.14655805 -0.67746528

C -2.63148958 1.87048400 0.55708785

C -1.59014985 2.84908856 0.55765388

C -2.97166043 1.22924971 1.79182598

C -2.63128382 1.86252819 3.03319205

C -3.25105613 -0.17927704 4.25934855

C -2.98462809 1.22590266 4.26351748

C -3.17326596 -0.90635114 5.48806127

C -3.26505264 -0.19262639 6.72970938

C -2.63584531 -2.22913287 7.96042871

C -3.20634271 -0.91549013 7.96198156

C -2.16480709 -2.77313342 9.19538557

C -2.66580983 -2.24128555 10.43406150

C 0.49663126 3.23872786 -10.57418725

C 1.20434333 3.07403765 -9.33312431

C -0.92598494 3.09128658 -8.10185338

C 0.49772304 3.23948347 -8.10135440

C -1.58314588 2.80017990 -6.86598444

C -0.92479186 3.10989981 -5.62823014

C -2.63540737 1.85510717 -4.38692480

C -1.58734523 2.82800930 -4.39018261

C -2.98103251 1.22889851 -3.15089650

C -2.62657241 1.86377224 -1.91415940

C -3.23520945 -0.17069968 -0.67207270

C -2.97543506 1.23344540 -0.67452531

C -3.14830718 -0.89184018 0.55637332

C -3.22847411 -0.17045070 1.78881795

C -2.56777654 -2.18889884 3.02436388

C -3.14736977 -0.88888422 3.02561101

C -2.12474335 -2.76123567 4.25377534

C -2.60621558 -2.21385765 5.48469564

C -0.93102687 -3.50358831 6.73103176

C -2.14963842 -2.76685580 6.72791892

C -0.22976408 -3.66192756 7.96656326

C -0.93593706 -3.49979352 9.19801395

C 1.19208333 -3.52282752 10.43807727

C -0.22740427 -3.67047369 10.43760390

C -0.92291242 3.09101435 -10.57467926

C -2.63744808 1.82329065 -9.32917635

C -1.58249584 2.78402698 -9.33412281

C -2.99236062 1.20174825 -8.09155305

C -2.63151872 1.83469103 -6.86127112

C -3.25901414 -0.18702004 -5.61198931

C -2.98143916 1.20938414 -5.61802075

C -3.17468640 -0.90108592 -4.37408464

C -3.24763663 -0.17293772 -3.14813738

C -2.57003263 -2.18048115 -1.91127248

C -3.14668186 -0.88454941 -1.91035909

C -2.12629021 -2.75063475 -0.68006838

C -2.58865013 -2.21097548 0.55626563

C -0.99763980 -3.64274196 1.79467023

C -2.10913236 -2.75182927 1.79114033

C -0.21950258 -3.76349116 3.01965430

C -0.91525261 -3.53086074 4.25054661

C 1.19133135 -3.52831017 5.49479953

C -0.22180811 -3.67536085 5.49432428

C 1.84905441 -3.21433610 6.73202214

C 1.19447214 -3.51374818 7.96703569

C 2.90427561 -2.24586899 9.19714821

C 1.85121456 -3.20985473 9.19899209

C 3.28426391 -1.62245889 10.43615408

C -3.31234836 -0.20274591 -10.55952753

C -3.01759304 1.19398127 -10.56531374

C -3.21281449 -0.92203706 -9.31780188

C -3.28245455 -0.20007987 -8.08539508

C -2.62595187 -2.21945519 -6.84682299

C -3.19359130 -0.91192982 -6.84876608

C -2.14228417 -2.76196094 -5.60926155

C -2.60323771 -2.21299695 -4.37049426

C -0.90916231 -3.52234648 -3.13390277

C -2.11693815 -2.75524920 -3.14164105

C -0.21859072 -3.76552100 -1.89147675

C -1.04401452 -3.67856109 -0.68864943

C 1.62215788 -3.93343177 0.55183053

C -0.55628126 -4.16078103 0.55123802

C 1.94648950 -3.33596211 1.79566220

C 1.20906879 -3.61469239 3.02010000

C 2.86628820 -2.24186542 4.25552339

C 1.84110737 -3.24395899 4.25152476

C 3.22388515 -1.60731755 5.48678523

C 2.88993767 -2.24253914 6.72969347

C 3.25416895 -1.61645968 7.96252281

C -2.17175903 -2.78996452 -10.55499523

C -2.63937701 -2.22870805 -9.31581893

C -0.92808961 -3.49834651 -8.08300398

C -2.15926479 -2.76815474 -8.08179119

C -0.22161654 -3.65581245 -6.85025495

C -0.92676922 -3.50085812 -5.61005614

C 1.19810653 -3.53096183 -4.37554819

C -0.22071871 -3.67863605 -4.37603027

C 1.83882430 -3.23627646 -3.13298072

C 1.21233848 -3.61648860 -1.89100337

C 2.86945203 -2.23054411 -0.67837446

C 2.00127677 -3.36125439 -0.68767946

C 3.20992494 -1.60747091 0.55831259

C 2.85095951 -2.23549508 1.79285170

C 3.18306015 -1.59044247 3.02645059

C -0.93955267 -3.51051242 -10.55565593

C 1.19669873 -3.50111056 -9.31661904

C -0.22233322 -3.64876128 -9.31713759

C 1.85554912 -3.20871378 -8.08202766

C 1.19582550 -3.50830739 -6.84974249

C 2.89057526 -2.23826583 -5.60749107

C 1.85320140 -3.21155451 -5.60910312

C 3.22790325 -1.60621550 -4.36848286

C 2.86283607 -2.23696869 -3.13989164

C 3.18721736 -1.58130302 -1.90930903

C 2.92855858 -2.25948119 -10.55314121

C 1.87099032 -3.21814042 -10.55462220

C 3.26979808 -1.61403249 -9.31369072

C 2.91009273 -2.24077532 -8.07996849

C 3.25302448 -1.60779815 -6.84476879

C -2.69898539 -2.27037730 -11.78725706

C -3.28366587 -0.94725313 -11.78936164

C -0.22497221 -3.71054769 -11.78679550

C 1.21375282 -3.56090748 -11.78628889

C 3.33855894 -1.64250523 -11.78504128

C 3.63854129 -0.22740108 -11.78686903

C 2.48094145 2.39101920 -11.79825009

C 1.23099126 3.11883869 -11.80401403

C -1.61596136 2.82269721 -11.80501956

C -2.68944039 1.85330466 -11.80008563

C -2.17285059 -2.78521931 -12.99880081

C -0.93052763 -3.50668922 -12.99877146

C 1.86317620 -3.21613404 -12.99771713

C 2.93048811 -2.25452351 -12.99693063

C 3.51542171 0.49282731 -13.00146707

C 2.93376377 1.80630801 -13.00725268

C -0.92435606 3.06784384 -13.01766118

C 0.50462480 3.21647504 -13.01715455

C -3.31042906 -0.21695226 -13.00394576

C -3.01142335 1.18808670 -13.00937818

C -0.25166411 -3.22574419 -14.24378271

C 1.14194801 -3.08081544 -14.24326255

C -2.83311049 -0.77820291 -14.24791350

C -2.26516788 -2.05877896 -14.24524944

C -2.34754442 1.49760338 -14.25584327

C -1.30573871 2.43438380 -14.25974370

C 1.00909548 2.67512820 -14.25893329

C 2.22134887 1.97268601 -14.25423266

C 2.87232039 -1.52458983 -14.24337117

C 3.16469762 -0.15456264 -14.24572569

C -1.07485447 -2.32678791 -15.02569824

C -2.23197720 0.28058759 -15.03222001

C -0.10986473 2.18764936 -15.03858734

C 2.35918706 0.75794581 -15.03058799

C 1.76318017 -2.03170214 -15.02466351

C 0.01393919 0.99945065 -15.78594664

C 1.27045277 0.27240832 -15.78249959

C 0.96690511 -1.14704145 -15.77917315

C -0.47704494 -1.29716970 -15.77969443

C -1.06612030 0.02946004 -15.78332553

C -0.96106944 -3.55599401 11.66836003

C -2.21053790 -2.82672786 11.66620709

C 1.88562833 -3.25991879 11.66934102

C 2.95832890 -2.28919850 11.66797237

C 3.55086451 0.51189188 11.67218409

C 2.96655107 1.83530762 11.67843359

C 0.49446382 3.27749186 11.68848167

C -0.94432365 3.12778233 11.68791986

C -3.06687759 1.20786582 11.67627862

C -3.36654778 -0.20743490 11.66971653

C -0.23441148 -3.66066778 12.88034298

C 1.19521848 -3.51198548 12.88082290

C 3.28115341 -1.63082443 12.88033103

C 3.57966817 -0.22501895 12.88236928

C 2.44291174 2.34344198 12.89333808

C 1.20053090 3.06602379 12.89853732

C -2.65979440 1.81281697 12.89146543

C -1.59262373 2.77552061 12.89750264

C -2.66270462 -2.24888332 12.87827929

C -3.24400766 -0.93451053 12.87999289

C 1.57706287 -2.88483867 14.12568133

C 2.61852187 -1.94736994 14.12549450

C -1.94997572 -2.42236050 14.12393192

C -0.73794577 -3.12555124 14.12488901

C -2.89277527 -0.29454662 14.12758835

C -2.60078068 1.07575977 14.13334647

C -0.87102937 2.63290055 14.14169789

C 0.52280894 2.77787723 14.14222344

C 3.10347609 0.32891159 14.12970416

C 2.53588386 1.60984716 14.13518390

C 0.38151063 -2.64189045 14.90636188

C -2.08699971 -1.21166155 14.90675786

C -1.49138246 1.57856190 14.91710970

C 1.34624158 1.87359693 14.91814238

C 2.50303993 -0.73443792 14.90836375

C -0.69485676 0.68923734 15.66577762

C 0.74912950 0.83937617 15.66630060

C 1.33803951 -0.48746133 15.66190661

C 0.25813318 -1.45763988 15.65994863

C -0.99825005 -0.73035037 15.66107501

C 0.63103369 -4.98536063 0.35248663

H 1.12174282 -7.21905041 0.28984978

C 1.31004049 -8.29549566 0.24188541

H 1.84528073 -8.61203058 1.13585148

H 1.90957732 -8.52128164 -0.63881955

H 0.36144414 -8.82685564 0.18023417

Table S11. The coordinate of the structures for “a” defective nanotube adsorbed with NH_3_ for physical adsorption.

C 3.32836751 0.94398730 10.51019394

C 3.25905314 0.95704364 5.56326526

C 3.19879909 1.67414609 6.80476957

C 3.29177337 0.95506031 8.03711906

C 2.64970016 2.97691488 9.27914772

C 3.22457943 1.66967990 9.27276923

C 2.18281859 3.53021319 10.52161305

C 3.23363105 0.96673105 0.63162905

C 3.16098413 1.68866208 1.86420816

C 3.23115061 0.96894422 3.10087748

C 2.63127856 3.00353504 4.33842194

C 3.18377618 1.68437217 4.33463272

C 2.15715253 3.53897130 5.57696147

C 2.63521156 2.98303409 6.81113690

C 0.93724411 4.25421641 8.05510824

C 2.16883440 3.52393624 8.04968802

C 0.23263535 4.39648782 9.29019898

C 0.95039982 4.25026437 10.52741995

C 3.26297418 0.96365597 -4.29881203

C 3.18182716 1.69077148 -3.07279556

C 3.23147995 0.97373411 -1.83504870

C 2.62345014 3.01007996 -0.59938028

C 3.16861353 1.69023024 -0.59673657

C 2.15460008 3.56197499 0.63212410

C 2.61942730 3.00463927 1.86695919

C 0.93397698 4.29283275 3.11050398

C 2.15468921 3.55356584 3.10806184

C 0.23059681 4.44459576 4.34640481

C 0.93569520 4.27132781 5.58088084

C -1.18674459 4.26326076 6.82197994

C 0.22989386 4.41524855 6.82243281

C -1.84455551 3.95573063 8.05424224

C -1.18694839 4.24413103 9.28975948

C -2.90958812 2.98364113 10.52001885

C -1.85808644 3.94883228 10.52656555

C 3.30896739 0.95285888 -9.24172251

C 3.22406033 1.67274614 -8.00926507

C 3.28465627 0.95736645 -6.77311711

C 2.63599055 2.98836652 -5.54250272

C 3.19653772 1.67975287 -5.53650407

C 2.16385948 3.54874662 -4.31166072

C 2.63055297 3.00693569 -3.07560869

C 0.93526517 4.29233877 -1.84168671

C 2.15252241 3.55516012 -1.83903551

C 0.22952461 4.45946071 -0.60338679

C 0.93339626 4.30414523 0.63197044

C -1.18860786 4.30136649 1.87051523

C 0.22687919 4.45326146 1.87101593

C -1.84641296 3.99452880 3.10954878

C -1.19203953 4.29196638 4.34591194

C -2.88342657 2.99811934 5.57531973

C -1.84513001 3.97298795 5.57998021

C -3.23335532 2.35329077 6.80925194

C -2.89315334 2.98075514 8.04806704

C -3.53185421 0.94452290 9.27061816

C -3.24764123 2.34400016 9.27727915

C -3.47994958 0.21320208 10.50802571

C 2.68032228 2.98324852 -10.48910687

C 3.25847853 1.67802817 -10.48295944

C 2.17665023 3.51989774 -9.25358346

C 2.65037303 2.98426788 -8.01563414

C 0.94158186 4.25840894 -6.79146124

C 2.16589658 3.52894725 -6.78576256

C 0.23249826 4.42460531 -5.55409388

C 0.93822718 4.28537430 -4.31554680

C -1.18838615 4.29555181 -3.08343479

C 0.23142728 4.44796388 -3.08286149

C -1.84407605 3.99402014 -1.84273437

C -1.19075670 4.30703591 -0.60388823

C -2.88247233 3.02144738 0.63033943

C -1.84654394 4.00581111 0.63099373

C -3.21929744 2.37813551 1.86490487

C -2.88251724 3.01311305 3.10629787

C -3.49213365 0.96798589 4.33238934

C -3.23228866 2.37437615 4.33647241

C -3.41198541 0.24118942 5.56108511

C -3.50614134 0.95460181 6.80259923

C -2.87166119 -1.08036764 8.03378573

C -3.44502033 0.23206371 8.03493554

C -2.40015866 -1.62356677 9.26895030

C -2.90234070 -1.09222829 10.50731431

C 0.23838188 4.40535110 -10.50110047

C 0.94626028 4.24251582 -9.25978986

C -1.18388764 4.25432934 -8.02853940

C 0.23938809 4.40728508 -8.02799602

C -1.83938082 3.95968784 -6.79252545

C -1.18268305 4.27260416 -5.55464994

C -2.88500966 3.00664424 -4.31358167

C -1.84365239 3.98665298 -4.31663456

C -3.22702186 2.37811785 -3.07780056

C -2.87721541 3.01529575 -1.84089202

C -3.47518073 0.97717346 -0.59914680

C -3.22263641 2.38265576 -0.60146882

C -3.38600814 0.25637617 0.62929816

C -3.46910817 0.97718912 1.86190037

C -2.80309083 -1.03976704 3.09764474

C -3.38567760 0.25891489 3.09860634

C -2.35974136 -1.61169682 4.32716435

C -2.84205402 -1.06507339 5.55801260

C -1.16516119 -2.35245006 6.80496106

C -2.38462318 -1.61722979 6.80135700

C -0.46363536 -2.50908334 8.04076660

C -1.17003186 -2.34813588 9.27227589

C 0.95778041 -2.36387147 10.51267228

C -0.46123576 -2.51615111 10.51220531

C -1.18067593 4.25284765 -10.50164860

C -2.88760342 2.97585159 -9.25553973

C -1.83872957 3.94327660 -9.26085460

C -3.23771509 2.35178233 -8.01790684

C -2.88088808 2.98689303 -6.78768789

C -3.49642218 0.96104202 -5.53905325

C -3.22647002 2.35885905 -5.54474051

C -3.41026224 0.24720114 -4.30130129

C -3.48634227 0.97491926 -3.07528955

C -2.80445502 -1.03110739 -1.83837111

C -3.38358279 0.26372107 -1.83745640

C -2.36059934 -1.60064382 -0.60700129

C -2.82323243 -1.06137682 0.62934595

C -1.23092574 -2.49146128 1.86806725

C -2.34331698 -1.60175576 1.86430311

C -0.45324016 -2.61122011 3.09345959

C -1.14945497 -2.38001121 4.32435171

C 0.95684999 -2.37165104 5.56869371

C -0.45572380 -2.52315364 5.56820174

C 1.61320896 -2.05437521 6.80588895

C 0.96010447 -2.35632441 8.04125928

C 2.66389142 -1.08010352 9.27062915

C 1.61554409 -2.04919448 9.27318277

C 3.04110319 -0.45429741 10.50919187

C -3.55267696 0.94626990 -10.48556539

C -3.26487478 2.34449227 -10.49138333

C -3.44898311 0.22691982 -9.24432329

C -3.51985400 0.94837484 -8.01187503

C -2.85915558 -1.07040717 -6.77416097

C -3.42816778 0.23641775 -6.77567016

C -2.37564803 -1.61279230 -5.53658069

C -2.83695337 -1.06389843 -4.29784720

C -1.14315897 -2.37283153 -3.06099615

C -2.35101956 -1.60582697 -3.06870522

C -0.45242782 -2.61445568 -1.81814646

C -1.27809010 -2.52823756 -0.61534694

C 1.38910726 -2.77579127 0.62532592

C -0.78910310 -3.00922368 0.62458585

C 1.71014239 -2.17605386 1.86906588

C 0.97470484 -2.45812526 3.09395090

C 2.62497373 -1.07694393 4.32884582

C 1.60524927 -2.08454991 4.32527818

C 2.97953004 -0.44042815 5.55990734

C 2.64892074 -1.07716537 6.80301712

C 3.01015715 -0.44918577 8.03570526

C -2.40619333 -1.63895905 -10.48276278

C -2.87373827 -1.07901924 -9.24299857

C -1.16186483 -2.34939622 -8.01121717

C -2.39296100 -1.61893653 -8.00942587

C -0.45518358 -2.50630708 -6.77834723

C -1.16038620 -2.35209364 -5.53778178

C 0.96393306 -2.37658544 -4.30288225

C -0.45455678 -2.52889972 -4.30337890

C 1.60277852 -2.07815409 -3.05999315

C 0.97808440 -2.46101206 -1.81765064

C 2.62679638 -1.06554771 -0.60524488

C 1.76580124 -2.20173641 -0.61427400

C 2.96351872 -0.44047007 0.63135831

C 2.60838490 -1.07055284 1.86599343

C 2.93762769 -0.42385617 3.09958216

C -1.17328725 -2.35813983 -10.48429968

C 0.96264605 -2.34479393 -9.24521709

C -0.45594893 -2.49724570 -9.24575424

C 1.62001824 -2.05050461 -8.01012497

C 0.96175941 -2.35408194 -6.77783327

C 2.64775659 -1.07338460 -5.53473978

C 1.61712815 -2.05381730 -5.53674811

C 2.98104894 -0.43926962 -4.29568898

C 2.62034083 -1.07224482 -3.06690563

C 2.94050809 -0.41454197 -1.83627869

C 2.68713662 -1.09173264 -10.48080184

C 1.63509651 -2.05636347 -10.48320224

C 3.02411156 -0.44545294 -9.24071189

C 2.66796120 -1.07530198 -8.00749297

C 3.00606232 -0.44046879 -6.77190697

C -2.93499501 -1.11929136 -11.71426694

C -3.52309306 0.20227640 -11.71552403

C -0.45863713 -2.55450313 -11.71612964

C 0.97951974 -2.39994720 -11.71555063

C 3.09455127 -0.47148208 -11.71192679

C 3.38855746 0.94485204 -11.71286927

C 2.22443176 3.56087776 -11.72433095

C 0.97302856 4.28634780 -11.73088490

C -1.87239031 3.98056258 -11.73197507

C -2.94117697 3.00588360 -11.72630506

C -2.40897305 -1.63227221 -12.92660762

C -1.16496486 -2.35076019 -12.92771841

C 1.62741203 -2.05071233 -12.92661290

C 2.69046116 -1.08438315 -12.92460892

C 3.26471858 1.66546685 -12.92719894

C 2.67930271 2.97731870 -12.93309566

C -1.18185920 4.22771330 -12.94483681

C 0.24664417 4.38125699 -12.94431417

C -3.55422121 0.93284030 -12.92983247

C -3.26080234 2.33912186 -12.93538799

C -0.48728754 -2.06647811 -14.17269537

C 0.90581914 -1.91680530 -14.17216560

C -3.07605885 0.37346331 -14.17432350

C -2.50410415 -0.90540886 -14.17254905

C -2.59884247 2.65119264 -14.18222868

C -1.56084560 3.59216216 -14.18663739

C 0.75290374 3.84076834 -14.18576520

C 1.96709271 3.14177161 -14.18047730

C 2.62997969 -0.35377377 -14.17053910

C 2.91724107 1.01740475 -14.17199887

C -1.31339428 -1.16934703 -14.95375407

C -2.47914675 1.43459095 -14.95869046

C -0.36424655 3.34927928 -14.96550368

C 2.10917417 1.92757665 -14.95693295

C 1.52313950 -0.86459223 -14.95264429

C -0.23632094 2.16147084 -15.71288252

C 1.02251518 1.43850682 -15.70946810

C 0.72400483 0.01792034 -15.70674475

C -0.71938693 -0.13714929 -15.70729424

C -1.31284319 1.18759011 -15.71035566

C -1.19519858 -2.40246354 11.74292094

C -2.44633841 -1.67611833 11.73994766

C 1.65014487 -2.09702902 11.74382388

C 2.71864870 -1.12170384 11.74160509

C 3.29997133 1.68194741 11.74405927

C 2.71228268 3.00390419 11.75023877

C 0.23664457 4.44071513 11.76100342

C -1.20172832 4.28631015 11.76054123

C -3.31598477 2.35680886 11.74835291

C -3.60963737 0.94022065 11.74189629

C -0.46845194 -2.50374568 12.95515417

C 0.96068735 -2.35034182 12.95561814

C 3.03929213 -0.46111803 12.95333374

C 3.33252706 0.94578940 12.95445453

C 2.18805202 3.51134650 12.96510619

C 0.94367479 4.23078802 12.97072995

C -2.91214750 2.96382775 12.96351600

C -1.84883166 3.93104633 12.96986772

C -2.90063121 -1.09874246 12.95141763

C -3.48587911 0.21382132 12.95228990

C 1.34031975 -1.72047000 14.19984307

C 2.37841428 -0.77922532 14.19896331

C -2.18808291 -1.26946247 14.19750034

C -0.97387843 -1.96891760 14.19910956

C -3.13780694 0.85545475 14.19983999

C -2.85064375 2.22683002 14.20524534

C -1.12645382 3.79033241 14.21382474

C 0.26689384 3.93991646 14.21424574

C 2.85540721 1.49889595 14.20174752

C 2.28364537 2.77805798 14.20686819

C 0.14392385 -1.48084874 14.98026511

C -2.32933928 -0.05875062 14.97962410

C -1.74310907 2.73362077 14.98893166

C 1.09330667 3.03813584 14.98980614

C 2.25895251 0.43383144 14.98111009

C -0.94361670 1.84716242 15.73769907

C 0.49975111 2.00215963 15.73816068

C 1.09313006 0.67734800 15.73442545

C 0.01653459 -0.29648127 15.73300198

C -1.24227011 0.42660868 15.73368832

C 0.40048243 -3.82985756 0.42265991

H 1.69563995 -7.21022793 0.56864617

N 0.70954226 -7.00057656 0.73470058

H 0.57501965 -5.98296792 0.76923398

H 0.14373684 -7.39472405 -0.01934162

Table S12. The coordinate of the structures for “a” defective nanotube adsorbed with H_2_O for physical adsorption.

C 3.57350525 -0.25442097 10.47174230

C 3.50667309 -0.25362379 5.52566436

C 3.45004905 0.46718419 6.76495351

C 3.53744553 -0.24940528 7.99907854

C 2.90875842 1.78007156 9.23524970

C 3.47452274 0.46882266 9.23272757

C 2.44576868 2.33977724 10.47618881

C 3.48460260 -0.26224533 0.59355987

C 3.41827427 0.46559926 1.82357522

C 3.48363758 -0.24757620 3.06410493

C 2.89376740 1.79400502 4.29461582

C 3.43788689 0.47167703 4.29546666

C 2.42211673 2.33604538 5.53128242

C 2.89542591 1.77995601 6.76722922

C 1.20623991 3.06711993 8.00707891

C 2.43221805 2.32733275 8.00416365

C 0.50258652 3.21792637 9.24183636

C 1.21882497 3.06912166 10.47977262

C 3.51943614 -0.28087893 -4.33709475

C 3.43982856 0.44797812 -3.11190772

C 3.48013987 -0.26656086 -1.87177078

C 2.88620912 1.78008153 -0.64390678

C 3.42127368 0.45647150 -0.63693009

C 2.42147543 2.33985357 0.58559348

C 2.88327410 1.78471632 1.82221785

C 1.20493759 3.08788504 3.06115886

C 2.42125341 2.34135568 3.06130365

C 0.50225261 3.24964453 4.29652002

C 1.20543625 3.07650505 5.53224513

C -0.91719840 3.08807203 6.77294124

C 0.50053603 3.22959457 6.77332798

C -1.57743065 2.78898205 8.00633136

C -0.91817974 3.07595433 9.24148102

C -2.64877810 1.82928259 10.47482815

C -1.59124499 2.78785186 10.47906440

C 3.56569734 -0.30349014 -9.28171172

C 3.48568997 0.41970499 -8.05042249

C 3.54288998 -0.29302176 -6.81245224

C 2.90318716 1.74353186 -5.58684219

C 3.45774851 0.43257390 -5.57714740

C 2.43304754 2.30882138 -4.35732499

C 2.89570847 1.76767320 -3.11943672

C 1.20758275 3.06912828 -1.89098716

C 2.41946937 2.32338325 -1.88562606

C 0.50279762 3.24602094 -0.65343793

C 1.20517521 3.09023619 0.58267410

C -0.91730368 3.10754689 1.82036908

C 0.49933708 3.24864645 1.82088347

C -1.57789486 2.81036061 3.06028195

C -0.92165967 3.10767484 4.29605745

C -2.62298360 1.83168680 5.52967804

C -1.57729971 2.79871375 5.53145404

C -2.97662938 1.19228767 6.76542515

C -2.63222884 1.82062677 8.00270989

C -3.28302718 -0.20874256 9.23048681

C -2.99061262 1.18905961 9.23350369

C -3.23619610 -0.93757548 10.46927380

C 2.94778905 1.72715607 -10.53398097

C 3.51889559 0.41895645 -10.52487115

C 2.44683548 2.26893703 -9.29946733

C 2.91808644 1.73381034 -8.06009189

C 1.21594871 3.01994960 -6.83964644

C 2.43635850 2.28379433 -6.83159350

C 0.50737974 3.19345589 -5.60315424

C 1.21207579 3.05306610 -4.36408861

C -0.91538064 3.08391819 -3.13346776

C 0.50560464 3.22551741 -3.13285300

C -1.57354845 2.79182340 -1.89211964

C -0.91853660 3.10449167 -0.65402165

C -2.62050464 1.83622305 0.58366493

C -1.57715363 2.81290343 0.58164179

C -2.96314226 1.20008064 1.82010763

C -2.62201388 1.83745437 3.05956130

C -3.24540468 -0.19756513 4.29309512

C -2.97665630 1.20681796 4.29265752

C -3.16809567 -0.92211960 5.52325143

C -3.25759727 -0.20486527 6.76267908

C -2.63556556 -2.24063355 7.99896419

C -3.20036452 -0.92470398 7.99668749

C -2.16869083 -2.78479749 9.23511877

C -2.66759908 -2.24698789 10.47185862

C 0.51451691 3.16452436 -10.54954347

C 1.22106032 2.99873768 -9.30768847

C -0.90942561 3.02828206 -8.07715567

C 0.51488407 3.17064798 -8.07640666

C -1.56786770 2.74173548 -6.84100915

C -0.90878350 3.05215008 -5.60387309

C -2.62171799 1.80330859 -4.35958624

C -1.57275824 2.77511374 -4.36534770

C -2.96809921 1.18077611 -3.12190753

C -2.61334715 1.82040384 -1.88771958

C -3.22243882 -0.20900409 -0.63968291

C -2.96332441 1.19497636 -0.64621629

C -3.14114951 -0.92582197 0.59091073

C -3.22321497 -0.19945950 1.82103321

C -2.57212536 -2.21310880 3.06613803

C -3.14526225 -0.91122553 3.06169964

C -2.12624881 -2.78504209 4.29454919

C -2.60488428 -2.23148237 5.52384665

C -0.93852407 -3.52885914 6.77239569

C -2.15196268 -2.78372813 6.76788592

C -0.23882755 -3.68878124 8.00907190

C -0.94434260 -3.51951457 9.24015265

C 1.18267999 -3.54759767 10.48152120

C -0.23740165 -3.68975849 10.48084770

C -0.90553945 3.02208904 -10.55032065

C -2.62236402 1.76044923 -9.30201161

C -1.56626056 2.71951977 -9.30909224

C -2.97859029 1.14239453 -8.06281998

C -2.61765490 1.77773394 -6.83399419

C -3.24890142 -0.24011746 -5.58002746

C -2.96842654 1.15539752 -5.58945387

C -3.16724559 -0.95123018 -4.33986024

C -3.23678629 -0.22119309 -3.11466304

C -2.55942860 -2.22695772 -1.86928087

C -3.13402231 -0.92928125 -1.87445159

C -2.11003304 -2.79318652 -0.63524976

C -2.58543782 -2.24704059 0.59502793

C -1.04443802 -3.71136094 1.84667826

C -2.12418422 -2.78563669 1.83569589

C -0.23055278 -3.80910025 3.05539271

C -0.91965513 -3.55943900 4.29050296

C 1.18332875 -3.56595047 5.53650697

C -0.23045887 -3.70644846 5.53583899

C 1.84134373 -3.25172820 6.77359344

C 1.18598813 -3.54666170 8.00970859

C 2.89701166 -2.27770431 9.23728687

C 1.84289421 -3.24083886 9.24135556

C 3.27763167 -1.65094206 10.47425509

C -3.30134027 -0.26686579 -10.52783221

C -3.00363048 1.12913734 -10.53683012

C -3.20347206 -0.98367858 -9.28442164

C -3.27129045 -0.25881931 -8.05331632

C -2.62294705 -2.27807347 -6.81030297

C -3.18537994 -0.96823276 -6.81514874

C -2.14260256 -2.81982718 -5.57115459

C -2.60269864 -2.26575543 -4.33297665

C -0.91726605 -3.58049104 -3.09725786

C -2.11949092 -2.80642099 -3.10253064

C -0.22283513 -3.80935662 -1.85916333

C -1.00206250 -3.69530288 -0.63472765

C 1.61573964 -3.99866440 0.60740533

C -0.56363946 -4.21225507 0.60650375

C 1.98583095 -3.41093111 1.84790347

C 1.20584048 -3.66701321 3.05602899

C 2.85989936 -2.28746830 4.29657808

C 1.83110290 -3.28605824 4.29168092

C 3.21732901 -1.64928420 5.52610914

C 2.88276990 -2.28060952 6.76996779

C 3.24725746 -1.65171767 8.00127528

C -2.17029849 -2.85845629 -10.51788212

C -2.63537091 -2.29234983 -9.27973020

C -0.93069504 -3.56728957 -8.04436065

C -2.15815288 -2.83127726 -8.04413431

C -0.22541707 -3.72498681 -6.81125471

C -0.93050144 -3.56392758 -5.57173608

C 1.19402753 -3.60209284 -4.33705788

C -0.22560701 -3.74291816 -4.33709868

C 1.83941223 -3.30663958 -3.09671255

C 1.20171740 -3.67072998 -1.85908644

C 2.84894982 -2.29745302 -0.63308923

C 1.94297765 -3.40234035 -0.63308592

C 3.20499310 -1.66805660 0.59759991

C 2.85962780 -2.28867532 1.83766658

C 3.18322671 -1.63798008 3.06833479

C -0.94139656 -3.58453639 -10.51740241

C 1.19421757 -3.58123236 -9.27795948

C -0.22508684 -3.72320547 -9.27832018

C 1.85394483 -3.28879211 -8.04348415

C 1.19276816 -3.58350431 -6.81097207

C 2.89124683 -2.31644206 -5.56934865

C 1.85095554 -3.28626941 -5.57092550

C 3.23021674 -1.68199748 -4.33067977

C 2.86345372 -2.30851047 -3.10068038

C 3.17722279 -1.65299413 -1.86696194

C 2.92927323 -2.34631417 -10.51624947

C 1.86955226 -3.30254713 -10.51655570

C 3.27125141 -1.69939411 -9.27759135

C 2.91060477 -2.32345623 -8.04242367

C 3.25471038 -1.68911737 -6.80808009

C -2.69558971 -2.33930825 -11.75104063

C -3.27540729 -1.01410781 -11.75584859

C -0.22763157 -3.78973635 -11.74817976

C 1.21157447 -3.64514845 -11.74781551

C 3.34087515 -1.73213544 -11.74899261

C 3.64403344 -0.31776739 -11.75312502

C 2.49539643 2.30496684 -11.77029243

C 1.24865908 3.03834903 -11.77874701

C -1.59867076 2.75228479 -11.78025421

C -2.67427329 1.78519890 -11.77293410

C -2.17164732 -2.85852549 -12.96179204

C -0.93242709 -3.58483377 -12.96062780

C 1.86206454 -3.30361903 -12.95984152

C 2.93170366 -2.34496385 -12.96023780

C 3.52400360 0.40090482 -12.96915611

C 2.94684235 1.71608874 -12.97786494

C -0.90595548 2.99204292 -12.99350263

C 0.52330350 3.13577079 -12.99269607

C -3.30052442 -0.28626672 -12.97206544

C -2.99766737 1.11769638 -12.98070398

C -0.25252919 -3.30839399 -14.20625901

C 1.14147881 -3.16800793 -14.20586596

C -2.82536347 -0.85183247 -14.21501285

C -2.26167514 -2.13427843 -14.20972462

C -2.33310127 1.42250805 -14.22810146

C -1.28887358 2.35644885 -14.23425991

C 1.02647164 2.58953312 -14.23302376

C 2.23599527 1.88262483 -14.22585544

C 2.87607270 -1.61681368 -14.20792145

C 3.17240110 -0.24765953 -14.21263530

C -1.07260488 -2.40802616 -14.98996484

C -2.22121060 0.20341340 -15.00173370

C -0.09362987 2.10389889 -15.01217843

C 2.37038892 0.66597930 -14.99974488

C 1.76602384 -2.12206507 -14.98899640

C 0.02665115 0.91355464 -15.75692976

C 1.28067465 0.18241468 -15.75146853

C 0.97283869 -1.23612743 -15.74539333

C -0.47157746 -1.38166621 -15.74592644

C -1.05628581 -0.05307312 -15.75243567

C -0.97088494 -3.56699068 11.71110913

C -2.21659471 -2.83144184 11.70598650

C 1.87612106 -3.28155800 11.71243262

C 2.95080880 -2.31311076 11.70823463

C 3.55013064 0.48687264 11.70366428

C 2.97143088 1.81279950 11.70624082

C 0.50642625 3.26741737 11.71299380

C -0.93290758 3.12320427 11.71268298

C -3.05934313 1.20774063 11.70455199

C -3.36200801 -0.20692746 11.70133849

C -0.24560699 -3.67022097 12.92407925

C 1.18465861 -3.52672386 12.92475465

C 3.27545330 -1.65148408 12.91832242

C 3.57805338 -0.24644171 12.91585165

C 2.45090630 2.32696763 12.91985157

C 1.21159439 3.05508809 12.92347575

C -2.65206791 1.81478220 12.91854885

C -1.58228647 2.77477348 12.92287161

C -2.66756967 -2.24816464 12.91585306

C -3.24394641 -0.93150618 12.91335858

C 1.56802924 -2.89615314 14.16746703

C 2.61237999 -1.96183847 14.16461666

C -1.95703360 -2.42077360 14.16265767

C -0.74772134 -3.12869908 14.16644116

C -2.89229957 -0.28936535 14.15960465

C -2.59588193 1.08011277 14.16188690

C -0.86142410 2.63198848 14.16747282

C 0.53291292 2.77200935 14.16781125

C 3.10459589 0.31304185 14.16163916

C 2.54140118 1.59607436 14.16338883

C 0.37305167 -2.64633164 14.94685542

C -2.09041053 -1.20726439 14.94185175

C -1.48531485 1.58119833 14.94493184

C 1.35290685 1.86630118 14.94574656

C 2.50089097 -0.74595942 14.94357625

C -0.69215302 0.69102154 15.69605796

C 0.75229642 0.83613551 15.69653919

C 1.33662302 -0.49273079 15.69607000

C 0.25345925 -1.45922876 15.69659773

C -1.00037194 -0.72754358 15.69522102

C 0.61924189 -5.06079175 0.80495924

H 1.42701794 -5.89305416 -1.57963439

O 0.79578832 -6.47169229 -1.11389043

H 0.66390873 -6.07460598 -0.19451587

Table S13. The coordinate of the structures for “b” defective nanotube adsorbed with CH_4_ for physical adsorption.

C 4.13443059 0.42634783 10.50215573

C 4.00374162 0.27779060 5.55413349

C 3.96265399 1.03119164 6.77687315

C 4.06713321 0.35135547 8.02976637

C 3.43747993 2.41340617 9.21841944

C 4.01776156 1.10778728 9.24278186

C 2.98697486 3.01147443 10.44424071

C 3.88634243 0.20493479 0.61396805

C 3.86677680 0.93611614 1.84433587

C 3.93594952 0.22441220 3.08406369

C 3.36400894 2.28846959 4.29730413

C 3.91584943 0.96860802 4.30699906

C 2.89430991 2.85399640 5.52141108

C 3.39130056 2.33631244 6.75914332

C 1.69819819 3.63876256 7.98212587

C 2.93349810 2.91495061 7.98236134

C 1.01362334 3.83350006 9.21780820

C 1.75495293 3.73324727 10.44399983

C 3.94664067 0.23694414 -4.31849192

C 3.87522326 0.94331381 -3.08124209

C 3.88257685 0.20735412 -1.85161211

C 3.34296574 2.26182428 -0.61403470

C 3.84977682 0.92886060 -0.61555624

C 2.87552168 2.81775425 0.61237463

C 3.34473400 2.26110373 1.84040062

C 1.64712256 3.54515063 3.06922493

C 2.87740313 2.82339094 3.06945186

C 0.94235479 3.70832089 4.29691914

C 1.66498536 3.57455881 5.52120238

C -0.44779483 3.61539509 6.77598626

C 0.97006926 3.75516413 6.75867745

C -1.09244438 3.37393501 8.02856679

C -0.40900011 3.70112050 9.24171100

C -2.13149259 2.52624165 10.54775050

C -1.06102289 3.46970643 10.50082897

C 4.08370106 0.37740326 -9.26436255

C 3.98315605 1.05362344 -8.01061945

C 4.01421730 0.29769167 -6.79465937

C 3.37052990 2.29879292 -5.52822120

C 3.91913158 0.98486212 -5.54107400

C 2.88816085 2.83663934 -4.29763035

C 3.35197894 2.27114381 -3.07421209

C 1.64585653 3.53785567 -1.84406183

C 2.87234320 2.81788061 -1.84376928

C 0.93050378 3.67761360 -0.61453770

C 1.64356347 3.54081362 0.61211585

C -0.48269901 3.48682758 1.84353721

C 0.92848633 3.67868479 1.83999355

C -1.13757504 3.19881954 3.08307650

C -0.47886304 3.54483206 4.30622501

C -2.18894372 2.33553130 5.58514326

C -1.12473122 3.28313404 5.55306736

C -2.54448597 1.76408478 6.85595929

C -2.16766493 2.42909658 8.07004764

C -2.81197657 0.45629241 9.38695912

C -2.51139088 1.85252906 9.33367923

C -2.72662809 -0.23098209 10.64921901

C 3.47876841 2.45405963 -10.45255308

C 4.05534538 1.14827473 -10.47988773

C 2.95280208 2.94517046 -9.21102231

C 3.41096712 2.36550803 -7.98967327

C 1.67870517 3.59375249 -6.75602698

C 2.90801114 2.87333105 -6.75559526

C 0.95089332 3.71745546 -5.52903871

C 1.65527122 3.55993721 -4.29800742

C -0.47932171 3.49718579 -3.08251238

C 0.93495230 3.68931422 -3.07487594

C -1.12514587 3.14412286 -1.85283694

C -0.48021739 3.46872742 -0.61650434

C -2.13744694 2.13411866 0.61799556

C -1.12978805 3.14645761 0.61297485

C -2.50961991 1.54392254 1.87133846

C -2.18405621 2.22711666 3.10096698

C -2.88088120 0.27633597 4.41996317

C -2.55622435 1.66777810 4.36709999

C -2.80070270 -0.38977617 5.69388648

C -2.84958687 0.37312423 6.91020266

C -2.17007806 -1.60291330 8.22410355

C -2.75894002 -0.29881641 8.17516108

C -1.66316635 -2.10218812 9.46060402

C -2.14385892 -1.53383125 10.68676988

C 1.03590457 3.88456819 -10.45327579

C 1.72088769 3.66657746 -9.21140475

C -0.43360413 3.64111829 -8.01203716

C 0.99051673 3.78347701 -7.99051407

C -1.10881303 3.29959593 -6.79620552

C -0.46357677 3.55385878 -5.54248639

C -2.17792477 2.23860310 -4.34329074

C -1.12988373 3.21309442 -4.31992338

C -2.50510637 1.55283638 -3.12109788

C -2.12787447 2.13570815 -1.86315802

C -2.65778148 0.05945144 -0.62408418

C -2.43631629 1.46841654 -0.62196421

C -2.64733496 -0.60688535 0.67305687

C -2.82293713 0.15023589 1.90488111

C -2.33041581 -1.82512669 3.33403796

C -2.82564363 -0.49087149 3.20786202

C -1.79162799 -2.31586211 4.56376426

C -2.22492491 -1.69425163 5.76540253

C -0.47702423 -2.90129879 7.00762559

C -1.70575048 -2.18108370 7.00722659

C 0.25388010 -3.02318889 8.22487475

C -0.42997089 -2.82470250 9.46103252

C 1.72178209 -2.83672632 10.65054954

C 0.30036382 -2.96579157 10.68755982

C -0.38503076 3.74883385 -10.48113623

C -2.14371281 2.45440792 -9.31241472

C -1.07201346 3.39727602 -9.26584716

C -2.52948704 1.78964294 -8.10302526

C -2.17271126 2.35234730 -6.83376259

C -2.86520747 0.29348460 -5.67096478

C -2.54002941 1.67839520 -5.61373375

C -2.82081585 -0.47739853 -4.46004437

C -2.82082484 0.15748160 -3.15957323

C -2.08793033 -1.89785951 -2.12598140

C -2.64000033 -0.60318538 -1.92720396

C -2.09453024 -1.90757724 0.87059968

C -0.62335632 -3.16514651 2.15789548

C -1.86192541 -2.43807431 2.15761747

C 0.14003317 -3.27477301 3.33471159

C -0.55155052 -3.04337768 4.56414631

C 1.62142760 -2.98164005 5.69523513

C 0.20195953 -3.11720967 5.76624323

C 2.31021187 -2.65011200 6.91163271

C 1.67941296 -2.89914278 8.17652705

C 3.43412929 -1.63007833 9.33531200

C 2.36350256 -2.57539264 9.38849311

C 3.83535572 -0.96885311 10.54939828

C -2.80465659 0.48955623 -10.64375476

C -2.50198382 1.88308578 -10.58532111

C -2.73879967 -0.27557701 -9.42768866

C -2.83831784 0.39245903 -8.16254826

C -2.21114156 -1.67443381 -7.02715223

C -2.78618381 -0.37090489 -6.95260505

C -1.77359331 -2.29540037 -5.82098866

C -2.32296436 -1.81414276 -4.59003500

C -0.61635278 -3.15197498 -3.41957818

C -1.85359269 -2.42618280 -3.41961452

C -0.04115301 -3.09863218 -2.12580888

C 1.35836637 -2.95714827 0.67395542

C -0.04672806 -3.11012794 0.87083582

C 2.10420099 -2.73916246 1.90608014

C 1.54623601 -3.05473926 3.20911283

C 3.29596139 -1.76184848 4.36853068

C 2.24196601 -2.72662116 4.42133548

C 3.69818519 -1.11404638 5.58645479

C 3.37397956 -1.70331226 6.85752152

C 3.76906694 -1.04865964 8.07154071

C -1.65267978 -2.09153409 -10.72330695

C -2.15339806 -1.57632965 -9.47973969

C -0.46160730 -2.88559091 -8.26490050

C -1.69647109 -2.16210845 -8.26509670

C 0.21509963 -3.09652908 -7.02691336

C -0.54072077 -3.01844569 -5.82087624

C 1.55679113 -3.04417402 -4.45937347

C 0.14725431 -3.26303342 -4.58984871

C 2.11027183 -2.73404986 -3.15870967

C 1.35815236 -2.94876963 -1.92641739

C 3.06439669 -1.75677125 -0.62074607

C 1.94478125 -2.64043140 -0.62311033

C 3.49768847 -1.16955779 0.61924165

C 3.16544448 -1.78293826 1.87268541

C 3.60130078 -1.16387489 3.10218805

C -0.42064049 -2.81292629 -10.72300334

C 1.69401708 -2.87202806 -9.42672392

C 0.27321692 -2.99754452 -9.47929376

C 2.32501400 -2.63242084 -8.16149532

C 1.63331027 -2.96074563 -6.95193760

C 3.29960593 -1.74387849 -5.61251723

C 2.25107302 -2.70527898 -5.67003926

C 3.61015005 -1.15400447 -4.34189586

C 3.17220402 -1.77536698 -3.11989373

C 3.49502863 -1.16083908 -1.86184237

C 3.46202625 -1.61029052 -10.58377166

C 2.39415542 -2.55535055 -10.64237229

C 3.78482246 -1.01837400 -9.31082710

C 3.39272704 -1.67978297 -8.10167678

C 3.70827680 -1.09352919 -6.83222107

C -2.15786592 -1.53053896 -11.94366010

C -2.74335363 -0.20827295 -11.90212469

C 0.31632352 -2.97942142 -11.94295899

C 1.75599667 -2.84342538 -11.90088764

C 3.89476875 -0.94973574 -11.78762750

C 4.20072508 0.46303684 -11.73482899

C 3.04531139 3.08275307 -11.67003838

C 1.79682867 3.81383349 -11.67038899

C -1.05342526 3.54045988 -11.73620886

C -2.13645689 2.58311833 -11.78916619

C -1.60721196 -2.00543911 -13.15967585

C -0.36669243 -2.73206557 -13.15930480

C 2.42910063 -2.46196935 -13.08760434

C 3.50334198 -1.50926246 -13.03042005

C 4.10035152 1.23116764 -12.92258339

C 3.52237361 2.54698751 -12.89058778

C -0.33381042 3.82844661 -12.92364183

C 1.09657823 3.96770235 -12.89113375

C -2.73885489 0.56518419 -13.08904648

C -2.43263870 1.96797076 -13.03195400

C 0.33785198 -2.41159380 -14.37882889

C 1.73213444 -2.27543215 -14.34110037

C -2.23468219 0.04820268 -14.34221239

C -1.67153994 -1.23455109 -14.37943239

C -1.73848940 2.31934616 -14.25081718

C -0.69166183 3.24875486 -14.19793116

C 1.62462766 3.47453435 -14.14253152

C 2.83421012 2.76604097 -14.14225473

C 3.47092563 -0.73239325 -14.24957657

C 3.76992282 0.63517189 -14.19687395

C -0.46583781 -1.48032283 -15.14371435

C -1.61108314 1.13248838 -15.07143060

C 0.51887388 3.02475166 -14.96193426

C 2.98273232 1.58136697 -14.96135502

C 2.37354729 -1.20169234 -15.07042569

C 0.65337578 1.86578691 -15.75178367

C 1.90616683 1.13185292 -15.75138585

C 1.59654574 -0.28509310 -15.80735141

C 0.15212030 -0.42601018 -15.84427715

C -0.43130806 0.90285605 -15.80792007

C -0.41274187 -2.81168731 11.92612942

C -1.66127576 -2.08023474 11.92566853

C 2.43732958 -2.54388984 11.86317602

C 3.51964800 -1.58565068 11.81120838

C 4.12330700 1.20842308 11.71035417

C 3.53848243 2.53147520 11.68087981

C 1.06601208 3.97999719 11.68026431

C -0.37398642 3.84297298 11.70918213

C -2.51555959 1.94944471 11.80948497

C -2.82189732 0.53667778 11.86159025

C 0.33347077 -2.88692952 13.12655956

C 1.76483174 -2.74879618 13.09427306

C 3.86339477 -0.88729465 12.99578022

C 4.16928719 0.51695293 12.94544878

C 3.03696736 3.08769961 12.88319257

C 1.79591534 3.81481485 12.88289621

C -2.07515428 2.59119756 12.99407484

C -1.00007560 3.54502957 12.94404331

C -2.09241434 -1.46584426 13.12568456

C -2.67209162 -0.14989387 13.09280393

C 2.17108126 -2.08411856 14.31099788

C 3.21837242 -1.15440357 14.26128860

C -1.35529309 -1.60101633 14.36084385

C -0.14550884 -2.30964813 14.36127283

C -2.29172444 0.52986574 14.30955878

C -1.99316971 1.89807595 14.25972351

C -0.25378851 3.44193863 14.17754159

C 1.14101173 3.57814423 14.14828081

C 3.71387807 1.11787083 14.17858019

C 3.15033180 2.40102974 14.14879474

C 0.99081561 -1.80677795 15.10442769

C -1.47293199 -0.36372684 15.10360091

C -0.86421390 2.42013038 15.00230986

C 1.97531728 2.69907838 14.94161171

C 3.12104250 0.08589925 15.00344283

C -0.05721997 1.55396923 15.76700641

C 1.38733602 1.69548235 15.73633781

C 1.97055405 0.36633253 15.76762425

C 0.88592726 -0.59709635 15.81908062

C -0.36694530 0.13674405 15.81870250

C -1.05840230 -2.48716599 -1.30307834

C -1.05824584 -2.48733851 0.04814371

H -1.91666038 -6.80143847 -0.77548328

C -1.69704968 -5.73579876 -0.73677322

H -1.14520578 -5.44321945 -1.62981381

H -2.62831847 -5.17351051 -0.68767363

H -1.09683583 -5.51759014 0.14598695

Table S14. The coordinate of the structures for “b” defective nanotube adsorbed with NH_3_ for physical adsorption.

C 4.41425014 0.77830252 10.80892282

C 4.28552118 0.62852171 5.86040083

C 4.24609870 1.38209446 7.08312319

C 4.34822637 0.70228572 8.33628215

C 3.72363024 2.76653559 9.52419807

C 4.30062152 1.45942838 9.54901333

C 3.27460281 3.36673572 10.74952230

C 4.16828784 0.55791312 0.91973231

C 4.15141433 1.28849942 2.15046089

C 4.21837290 0.57600348 3.39001014

C 3.65118805 2.64085903 4.60354564

C 4.20014862 1.31980531 4.61325172

C 3.18224487 3.20718841 5.82739617

C 3.67788457 2.68861309 7.06514081

C 1.98796923 3.99610649 8.28715700

C 3.22120178 3.26884784 8.28794875

C 1.30393338 4.19420965 9.52237835

C 2.04489401 4.09248353 10.74854354

C 4.22820493 0.59227598 -4.01254427

C 4.15945958 1.29838265 -2.77509441

C 4.16391356 0.56181334 -1.54580915

C 3.63158959 2.61729499 -0.30744707

C 4.13424886 1.28271848 -0.30942049

C 3.16501313 3.17333294 0.91912282

C 3.63269834 2.61480673 2.14681961

C 1.93737057 3.90173372 3.37534311

C 3.16606016 3.17739348 3.37588738

C 1.23276199 4.06690807 4.60256827

C 1.95482337 3.93081099 5.82679833

C -0.15815171 3.98074977 7.08055695

C 1.26027301 4.11440315 7.06377005

C -0.80401221 3.74293156 8.33294488

C -0.11922871 4.06797991 9.54582459

C -1.84650524 2.90078652 10.85183759

C -0.77241814 3.84013369 10.80483068

C 4.36478938 0.73495147 -8.95784440

C 4.26660705 1.41082549 -7.70392973

C 4.29526522 0.65408404 -6.48850554

C 3.65862377 2.65661806 -5.22126985

C 4.20318038 1.34096470 -5.23469388

C 3.17785160 3.19507937 -3.99049220

C 3.64052466 2.62788279 -2.76746338

C 1.93709804 3.89741662 -1.53701521

C 3.16227594 3.17521135 -1.53683237

C 1.22187227 4.03792778 -0.30769275

C 1.93449665 3.89890733 0.91876974

C -0.19234573 3.84992289 2.14925624

C 1.21929144 4.03775754 2.14621051

C -0.84876020 3.56464130 3.38832352

C -0.18896770 3.90858079 4.61132788

C -1.90470084 2.70748113 5.88976333

C -0.83634158 3.65040882 5.85784467

C -2.26253788 2.13811642 7.16067927

C -1.88301829 2.80241624 8.37435252

C -2.53365111 0.83257275 9.69165993

C -2.22870798 2.22774860 9.63811964

C -2.45060610 0.14566197 10.95409158

C 3.76730553 2.81445482 -10.14452665

C 4.33948716 1.50675794 -10.17269521

C 3.24244551 3.30658160 -8.90302644

C 3.69867763 2.72452227 -7.68224798

C 1.97004678 3.95701583 -6.44807773

C 3.19726600 3.23309122 -6.44805762

C 1.24240769 4.08150660 -5.22126881

C 1.94654702 3.92097577 -3.99061832

C -0.18827224 3.86263422 -2.77546792

C 1.22650053 4.05113877 -2.76756663

C -0.83528343 3.51040394 -1.54633563

C -0.18925985 3.83232988 -0.31013567

C -1.85196792 2.50360529 0.92337151

C -0.84017208 3.51174391 0.91875383

C -2.22727917 1.91519542 2.17645375

C -1.89956713 2.59767678 3.40586847

C -2.60405215 0.65022968 4.72504150

C -2.27437893 2.04047133 4.67200662

C -2.52568408 -0.01552632 5.99911387

C -2.57214990 0.74815772 7.21512599

C -1.89789057 -1.22910951 8.52949779

C -2.48318175 0.07657514 8.48022912

C -1.39283171 -1.72944968 9.76616642

C -1.87226285 -1.15908947 10.99202088

C 1.32907568 4.25337355 -10.14484197

C 2.01297857 4.03214046 -8.90305213

C -0.14200509 4.01259728 -7.70416543

C 1.28258972 4.15024183 -7.68233662

C -0.81855873 3.67182962 -6.48883428

C -0.17254588 3.92224790 -5.23493922

C -1.89145048 2.61122826 -4.03701850

C -0.83994054 3.58190563 -4.01288515

C -2.22131050 1.92554088 -2.81539215

C -1.84190737 2.50595161 -1.55718487

C -2.38062937 0.43113718 -0.31903036

C -2.15326183 1.83917186 -0.31654741

C -2.37265740 -0.23524109 0.97821365

C -2.54585628 0.52268444 2.20989241

C -2.05865098 -1.45303076 3.63984298

C -2.55075218 -0.11771031 3.51326231

C -1.52097229 -1.94452732 4.86963257

C -1.95300955 -1.32136998 6.07101008

C -0.20797877 -2.53251411 7.31344034

C -1.43479762 -1.80891623 7.31287998

C 0.52192980 -2.65670194 8.53096618

C -0.16192160 -2.45579486 9.76687117

C 1.98915722 -2.47524255 10.95714388

C 0.56724587 -2.59909716 10.99377895

C -0.09233997 4.12262178 -10.17318701

C -1.85558897 2.83286805 -9.00585110

C -0.78085843 3.77220116 -8.95836560

C -2.24383949 2.16815302 -7.79716067

C -1.88565789 2.72819016 -6.52722442

C -2.58573813 0.67027180 -5.36603915

C -2.25555447 2.05397664 -5.30801665

C -2.54435501 -0.10198601 -4.15566741

C -2.54237333 0.53140993 -2.85444867

C -1.81812621 -1.52805826 -1.82168007

C -2.36492308 -0.23100150 -1.62245310

C -1.82392542 -1.53771452 1.17608907

C -0.35500508 -2.79755082 2.46385939

C -1.59165141 -2.06744065 2.46356085

C 0.40839331 -2.90829549 3.64059324

C -0.28254448 -2.67483268 4.86996427

C 1.89062345 -2.62065540 6.00108840

C 0.47065432 -2.75069994 6.07212022

C 2.58038253 -2.29216213 7.21793772

C 1.94797485 -2.53823216 8.48281212

C 3.70705428 -1.27604932 9.64219718

C 2.63256303 -2.21694536 9.69519246

C 4.10992868 -0.61574032 10.85636932

C -2.52248942 0.87151505 -10.33878921

C -2.21526758 2.26403140 -10.27939093

C -2.45962595 0.10501150 -9.12337803

C -2.55734324 0.77203885 -7.85766708

C -1.93808759 -1.29831361 -6.72354968

C -2.50850675 0.00720202 -6.64833730

C -1.50309197 -1.92193695 -5.51764485

C -2.05104112 -1.44022974 -4.28631213

C -0.34894921 -2.78412125 -3.11602307

C -1.58416473 -2.05492896 -3.11597981

C 0.22553025 -2.73564388 -1.82119644

C 1.62656510 -2.59248894 0.97921837

C 0.22083615 -2.74666267 1.17582092

C 2.37336563 -2.37838930 2.21116523

C 1.81511632 -2.69271259 3.51456528

C 3.57064194 -1.40809455 4.67430055

C 2.51224779 -2.36808980 4.72700421

C 3.97534081 -0.76230549 5.89265278

C 3.64826204 -1.34997555 7.16397180

C 4.04522917 -0.69667444 8.37823997

C -1.37899605 -1.71324237 -10.41980636

C -1.87854371 -1.19760513 -9.17610010

C -0.19166243 -2.51350239 -7.96148693

C -1.42419463 -1.78612582 -7.96173176

C 0.48394849 -2.72732989 -6.72327794

C -0.27209561 -2.64811035 -5.51748151

C 1.82474966 -2.67926548 -4.15504985

C 0.41488641 -2.89485981 -4.28626387

C 2.37827359 -2.37069979 -2.85392470

C 1.62560040 -2.58314357 -1.62188327

C 3.33661105 -1.39890579 -0.31556071

C 2.21310359 -2.27764991 -0.31826874

C 3.77339939 -0.81472617 0.92461397

C 3.43901591 -1.42711952 2.17799488

C 3.87828279 -0.81103265 3.40784913

C -0.14956500 -2.43900340 -10.41947250

C 1.96447812 -2.50629690 -9.12264219

C 0.54325488 -2.62705240 -9.17563840

C 2.59571846 -2.26948763 -7.85693209

C 1.90247820 -2.59586001 -6.64767066

C 3.57272954 -1.38526082 -5.30736479

C 2.52058276 -2.34277354 -5.36547681

C 3.88549965 -0.79718842 -4.03651924

C 3.44435180 -1.41673379 -2.81476140

C 3.77003809 -0.80459021 -1.55647084

C 3.73709606 -1.24990011 -10.27838554

C 2.66604977 -2.19131030 -10.33794079

C 4.06135582 -0.65979786 -9.00507830

C 3.66665870 -1.32055014 -7.79640837

C 3.98414713 -0.73604117 -6.52674022

C -1.88186038 -1.14951357 -11.63988627

C -2.46312118 0.17463680 -11.59778396

C 0.58720602 -2.60716240 -11.63932233

C 2.02739970 -2.47642246 -11.59687081

C 4.17253983 -0.58987992 -11.48150460

C 4.48327747 0.82182717 -11.42788901

C 3.33649040 3.44560435 -11.36154546

C 2.09047752 4.18093290 -11.36157696

C -0.76085487 3.91753849 -11.42869093

C -1.84716629 2.96394155 -11.48262477

C -1.33243959 -1.62535148 -12.85596732

C -0.09444706 -2.35626165 -12.85566884

C 2.70228930 -2.09619446 -12.78300534

C 3.77964301 -1.14706837 -12.72479280

C 4.38611075 1.59130128 -12.61501003

C 3.81233795 2.90903208 -12.58218986

C -0.03961152 4.20386309 -12.61555565

C 1.39126819 4.33803350 -12.58246531

C -2.45575588 0.94896804 -12.78411808

C -2.14472863 2.35067787 -12.72600128

C 0.61160869 -2.03719772 -14.07470959

C 2.00638784 -1.90617083 -14.03656216

C -1.95268070 0.43124813 -14.03740917

C -1.39373185 -0.85328351 -14.07511991

C -1.44881886 2.70052984 -13.94430425

C -0.39889415 3.62632799 -13.89042568

C 1.91821922 3.84407689 -13.83400250

C 3.12537277 3.13137425 -13.83387029

C 3.75036394 -0.36913610 -13.94336418

C 4.05405600 0.99735264 -13.88978579

C -0.18860364 -1.10250685 -14.83903949

C -1.32499477 1.51387332 -14.76560251

C 0.81119401 3.39877479 -14.65411275

C 3.27031273 1.94680580 -14.65375840

C 2.65164965 -0.83403414 -14.76492321

C 0.94220733 2.23992251 -15.44467546

C 2.19252991 1.50163883 -15.44447689

C 1.87803653 0.08580245 -15.50136966

C 0.43318277 -0.04993224 -15.53872557

C -0.14570233 1.28080936 -15.50169228

C -0.14598860 -2.44206801 12.23200964

C -1.39209313 -1.70649780 12.23123617

C 2.70506992 -2.18465877 12.17014351

C 3.79100969 -1.23051957 12.11835678

C 4.40498972 1.56113903 12.01672000

C 3.82445209 2.88597240 11.98650988

C 1.35654230 4.34276402 11.98429192

C -0.08400915 4.21140334 12.01296238

C -2.23267749 2.32589455 12.11368519

C -2.54372152 0.91423599 12.16609065

C 0.59938287 -2.51931013 13.43275264

C 2.03118033 -2.38633846 13.40095618

C 4.13660930 -0.53286792 13.30285553

C 4.44760511 0.87018419 13.25216569

C 3.32421357 3.44454398 13.18827999

C 2.08562862 4.17588950 13.18715038

C -1.79019606 2.96664517 13.29805427

C -0.71160453 3.91651194 13.24778243

C -1.82159314 -1.09006010 13.43096364

C -2.39671567 0.22785339 13.39764968

C 2.43927413 -1.72284088 14.61769911

C 3.49001714 -0.79713568 14.56823380

C -1.08541464 -1.22727165 14.66644428

C 0.12189682 -1.94001750 14.66721292

C -2.01442546 0.90680639 14.61428419

C -1.71089526 2.27387019 14.56398774

C 0.03415303 3.81124391 14.48137617

C 1.42948878 3.94246264 14.45247166

C 3.99382183 1.47319814 14.48485678

C 3.43479648 2.75828599 14.45429691

C 1.25972406 -1.44105042 15.41066043

C -1.19904063 0.01065823 15.40890830

C -0.58017206 2.79214993 15.30650120

C 2.26045634 3.06089250 15.24651725

C 3.39677273 0.44373753 15.30989539

C 0.22346125 1.92351275 16.07182012

C 1.66853152 2.05976287 16.04143589

C 2.24693523 0.72857742 16.07340058

C 1.15885455 -0.23087894 16.12501879

C -0.09140462 0.50744053 16.12405180

C -0.79174861 -2.12309044 -0.99839832

C -0.79090857 -2.12280655 0.35304779

H 0.14140289 -5.03502263 -1.17535500

H 0.12520837 -5.01463562 0.49544957

N 0.00723315 -5.60236690 -0.33426399

H -0.93141713 -6.00563481 -0.33835956

Table S15. The coordinate of the structures for “b” defective nanotube adsorbed with H_2_O for physical adsorption.

C 3.32009466 -0.39366068 10.49337809

C 3.19107185 -0.53979366 5.54518679

C 3.14927263 0.21291282 6.76834322

C 3.25333944 -0.46748166 8.02099440

C 2.62357855 1.59405914 9.21044024

C 3.20382888 0.28835480 9.23428201

C 2.17284076 2.19144010 10.43651843

C 3.07656406 -0.60969235 0.60473193

C 3.05636360 0.12067244 1.83549623

C 3.12471764 -0.59165623 3.07499232

C 2.55195948 1.47152859 4.28910095

C 3.10406164 0.15171383 4.29841135

C 2.08136241 2.03608715 5.51330449

C 2.57787757 1.51801306 6.75109212

C 0.88419117 2.81934615 7.97416214

C 2.11976663 2.09603097 7.97449820

C 0.19939515 3.01310345 9.20980594

C 0.94058092 2.91281922 10.43619695

C 3.13911663 -0.57559034 -4.32806559

C 3.06737534 0.13029185 -3.09051101

C 3.07368916 -0.60614694 -1.86102015

C 2.53379805 1.44775326 -0.62260021

C 3.04067775 0.11481755 -0.62452605

C 2.06539842 2.00268471 0.60396306

C 2.53418549 1.44560101 1.83196114

C 0.83483827 2.72743485 3.06077940

C 2.06571380 2.00679483 3.06117759

C 0.12954244 2.88976770 4.28833398

C 0.85172433 2.75609838 5.51292256

C -1.26126328 2.79467067 6.76702822

C 0.15642273 2.93565890 6.75038185

C -1.90591449 2.55231400 8.01945166

C -1.22319138 2.87961450 9.23304181

C -2.94513855 1.70297508 10.53805145

C -1.87517048 2.64705628 10.49195013

C 3.27726412 -0.43319102 -9.27413220

C 3.17659158 0.24255498 -8.02016084

C 3.20740851 -0.51378277 -6.80438083

C 2.56340013 1.48670119 -5.53737547

C 3.11218563 0.17282451 -5.55048006

C 2.08025405 2.02368208 -4.30662282

C 2.54389983 1.45809848 -3.08311449

C 0.83605611 2.72245810 -1.85302429

C 2.06335727 2.00392457 -1.85249785

C 0.12005195 2.86085792 -0.62355306

C 0.83262115 2.72448724 0.60348595

C -1.29416130 2.66728619 1.83395700

C 0.11675186 2.86090804 1.83111913

C -1.94918854 2.37815883 3.07329062

C -1.29151710 2.72464380 4.29699953

C -3.00102393 1.51368559 5.57490979

C -1.93741856 2.46204510 5.54363575

C -3.35664285 0.94188657 6.84561261

C -2.98034156 1.60655914 8.06013967

C -3.62467087 -0.36674027 9.37613359

C -3.32426806 1.02957817 9.32349148

C -3.53973495 -1.05451408 10.63815331

C 2.67282095 1.64374504 -10.46193252

C 3.24932397 0.33796828 -10.48948000

C 2.14666997 2.13461661 -9.22036503

C 2.60453679 1.55452052 -7.99899839

C 0.87133079 2.78128013 -6.76530861

C 2.10106311 2.06155830 -6.76476382

C 0.14278526 2.90349754 -5.53849862

C 0.84666961 2.74577253 -4.30717547

C -1.28836045 2.67995343 -3.09234852

C 0.12567881 2.87375322 -3.08415916

C -1.93432985 2.32536826 -1.86306947

C -1.29034548 2.64996177 -0.62626392

C -2.94730493 1.31365222 0.60719017

C -1.94016874 2.32663909 0.60290855

C -3.31960482 0.72287386 1.86020889

C -2.99494520 1.40567379 3.09039991

C -3.69254930 -0.54529395 4.40869714

C -3.36755029 0.84616513 4.35642993

C -3.61280426 -1.21163088 5.68250017

C -3.66159224 -0.44907981 6.89920344

C -2.98266570 -2.42560528 8.21275282

C -3.57131847 -1.12137522 8.16404210

C -2.47611104 -2.92530293 9.44917367

C -2.95714336 -2.35743453 10.67546528

C 0.22973023 3.07337938 -10.46311599

C 0.91453890 2.85566792 -9.22095299

C -1.24033894 2.82737359 -8.02227700

C 0.18363501 2.97131108 -8.00013786

C -1.91577689 2.48448351 -6.80680522

C -1.27150273 2.73848155 -5.55250013

C -2.98556178 1.42118959 -4.35451334

C -1.93804582 2.39622939 -4.33029472

C -3.31303589 0.73453361 -3.13271574

C -2.93652401 1.31654242 -1.87413646

C -3.46685347 -0.76056373 -0.63606513

C -3.24531961 0.64846497 -0.63327451

C -3.45726124 -1.42732821 0.66087967

C -3.63297225 -0.67085086 1.89302793

C -3.14127276 -2.64607111 3.32232638

C -3.63656946 -1.31202681 3.19622189

C -2.60362390 -3.13751540 4.55210440

C -3.03730102 -2.51627022 5.75385121

C -1.28898404 -3.72293558 6.99613878

C -2.51808953 -3.00333523 6.99572177

C -0.55840254 -3.84491697 8.21357752

C -1.24271269 -3.64746622 9.44961537

C 0.90876399 -3.65832030 10.63958474

C -0.51254173 -3.78850244 10.67625407

C -1.19104349 2.93637550 -10.49156601

C -2.94887039 1.63992421 -9.32389880

C -1.87794384 2.58355782 -9.27656122

C -3.33485498 0.97447396 -8.11496775

C -2.97905375 1.53655335 -6.84517000

C -3.67191464 -0.52306408 -5.68351527

C -3.34679611 0.86182654 -5.62558145

C -3.62827695 -1.29456642 -4.47298175

C -3.62862734 -0.66084492 -3.17193315

C -2.89717581 -2.71749498 -2.13924091

C -3.44829757 -1.42251587 -1.93980625

C -2.90483142 -2.72872991 0.85842293

C -1.43399544 -3.98613079 2.14614039

C -2.67213694 -3.25862760 2.14563629

C -0.67035790 -4.09387034 3.32334587

C -1.36280806 -3.86398190 4.55251375

C 0.80987337 -3.80058605 5.68392159

C -0.60943088 -3.93761157 5.75468338

C 1.49795434 -3.46912072 6.90082152

C 0.86702485 -3.71938538 8.16546056

C 2.62049782 -2.44973766 9.32528957

C 1.55049075 -3.39578238 9.37775754

C 3.02126594 -1.78891351 10.53975485

C -3.60875705 -0.32449905 -10.65631693

C -3.30654728 1.06903303 -10.59716902

C -3.54327585 -1.09013978 -9.44057419

C -3.64353070 -0.42272446 -8.17520936

C -3.01703662 -2.49002786 -7.04064289

C -3.59216361 -1.18665685 -6.96561788

C -2.57988071 -3.11133459 -5.83455362

C -3.13016898 -2.63115850 -4.60358929

C -1.42372234 -3.96910936 -3.43306764

C -2.66128075 -3.24412339 -3.43356763

C -0.85005509 -3.91750294 -2.13860576

C 0.54857729 -3.77202224 0.66232910

C -0.85796220 -3.93714419 0.85704901

C 1.29256689 -3.55372499 1.89464975

C 0.73519711 -3.87137579 3.19775185

C 2.48392369 -2.57858778 4.35825712

C 1.43046179 -3.54409309 4.41029338

C 2.88553551 -1.93168923 5.57669435

C 2.56091364 -2.52156938 6.84743638

C 2.95543522 -1.86750645 8.06192415

C -2.45648292 -2.90537221 -10.73656934

C -2.95796173 -2.39083218 -9.49303584

C -1.26637811 -3.69992430 -8.27787658

C -2.50158953 -2.97705409 -8.27842942

C -0.59010913 -3.91050808 -7.03962600

C -1.34655573 -3.83326781 -5.83397985

C 0.74981913 -3.85758821 -4.47120649

C -0.65926993 -4.07801544 -4.60269000

C 1.30151363 -3.54716425 -3.16990423

C 0.54854986 -3.76325925 -1.93835190

C 2.25352182 -2.57015560 -0.63112609

C 1.13428565 -3.45445605 -0.63426246

C 2.68683972 -1.98394616 0.60914829

C 2.35388223 -2.59757712 1.86212084

C 2.78951429 -1.97977299 3.09234907

C -1.22426340 -3.62642266 -10.73604324

C 0.88972893 -3.68414626 -9.43856171

C -0.53089240 -3.81105105 -9.49195576

C 1.51983696 -3.44428978 -8.17289466

C 0.82788019 -3.77346926 -6.96373326

C 2.49247097 -2.55578302 -5.62303028

C 1.44454600 -3.51770950 -5.68131083

C 2.80214971 -1.96644211 -4.35192685

C 2.36302057 -2.58802906 -3.13035609

C 2.68505649 -1.97398899 -1.87188347

C 2.65730660 -2.42094875 -10.59437901

C 1.59021657 -3.36677180 -10.65386156

C 2.97899686 -1.82902492 -9.32114924

C 2.58685462 -2.49096947 -8.11225923

C 2.90161857 -1.90502730 -6.84246782

C -2.96112862 -2.34386151 -11.95690269

C -3.54696283 -1.02171818 -11.91500026

C -0.48661595 -3.79185671 -11.95574232

C 0.95297555 -3.65498992 -11.91283316

C 3.09031705 -1.75981474 -11.79778744

C 3.39557589 -0.34692432 -11.74447481

C 2.23963915 2.27252550 -11.67944944

C 0.99095764 3.00325727 -11.68006515

C -1.85897566 2.72794445 -11.74694518

C -2.94135165 1.76986806 -11.80062502

C -2.40991654 -2.81803624 -13.17288489

C -1.16912478 -3.54414364 -13.17231867

C 1.62644831 -3.27260633 -13.09906232

C 2.69997624 -2.31914187 -13.04095215

C 3.29563847 0.42143699 -12.93207643

C 2.71711365 1.73699140 -12.89995848

C -1.13930897 3.01674860 -12.93411188

C 0.29096018 3.15676428 -12.90108062

C -3.54247566 -0.24777315 -13.10152497

C -3.23696068 1.15513424 -13.04375434

C -0.46416529 -3.22276870 -14.39136730

C 0.93000099 -3.08595974 -14.35281241

C -3.03775026 -0.76401806 -14.35476003

C -2.47406990 -2.04650697 -14.39228255

C -2.54274299 1.50732564 -14.26233626

C -1.49650927 2.43731817 -14.20880247

C 0.81963178 2.66410083 -14.15246403

C 2.02949273 1.95609439 -14.15192479

C 2.66790412 -1.54194598 -14.25992223

C 2.96624288 -0.17430494 -14.20674066

C -1.26789383 -2.29142395 -15.15610418

C -2.41446955 0.32085958 -15.08332029

C -0.28561327 2.21409876 -14.97247338

C 2.17906610 0.77175892 -14.97134711

C 1.57117340 -2.01157217 -15.08145093

C -0.15014990 1.05542957 -15.76258274

C 1.10303882 0.32208841 -15.76199438

C 0.79416352 -1.09502511 -15.81846660

C -0.65020316 -1.23654041 -15.85597869

C -1.23429436 0.09202429 -15.81942713

C -1.22592535 -3.63516329 11.91477899

C -2.47471267 -2.90406777 11.91434436

C 1.62397004 -3.36550128 11.85245898

C 2.70575303 -2.40653671 11.80116409

C 3.30882240 0.38779800 11.70194678

C 2.72412245 1.71088479 11.67308157

C 0.25115861 3.15839304 11.67246357

C -1.18878148 3.02018479 11.70078437

C -3.32944088 1.12550378 11.79941006

C -3.63545976 -0.28734287 11.85083118

C -0.47985767 -3.71026872 13.11532764

C 0.95145254 -3.57126701 13.08336043

C 3.04913465 -1.70872035 12.98612158

C 3.35467918 -0.30434828 12.93662160

C 2.22211642 2.26619690 12.87559197

C 0.98070631 2.99278903 12.87531411

C -2.88981998 1.76691405 12.98447736

C -1.81511951 2.72126208 12.93524925

C -2.90615366 -2.29008851 13.11451806

C -3.48599757 -0.97419283 13.08193925

C 1.35725591 -2.90703608 14.30048489

C 2.40412131 -1.97685728 14.25140879

C -2.16929278 -2.42545938 14.34983649

C -0.95928187 -3.13365401 14.35024676

C -3.10630670 -0.29484430 14.29911212

C -2.80811751 1.07344516 14.24991666

C -1.06926446 2.61805980 14.16896875

C 0.32547761 2.75499322 14.14032837

C 2.89902625 0.29567744 14.17003644

C 2.33525953 1.57871344 14.14081631

C 0.17675864 -2.63066759 15.09388094

C -2.28754739 -1.18850270 15.09310239

C -1.67960556 1.59564597 14.99304777

C 1.15984111 1.87580780 14.93343462

C 2.30631112 -0.73700138 14.99415783

C -0.87256701 0.72944604 15.75761447

C 0.57191358 0.87154605 15.72738366

C 1.15556660 -0.45742759 15.75821248

C 0.07126970 -1.42130075 15.80901500

C -1.18186825 -0.68791895 15.80862122

C -1.86987851 -3.31060527 -1.31587290

C -1.87029795 -3.31081404 0.03563558

H -1.04484354 -5.99474868 0.21341417

O -1.30088614 -6.88646869 -0.07552783

H -2.13996080 -7.09175339 0.36073928

Table S16. The coordinate of the structures for “c” defective nanotube adsorbed with CH_4_ for physical adsorption.

C 3.65040953 -1.46173053 10.54767857

C 3.88155465 -1.73366884 5.61030584

C 3.74044574 -0.95410140 6.81165655

C 3.75808509 -1.60423056 8.08008687

C 3.01815357 0.47891292 9.17393483

C 3.60890069 -0.81513644 9.26653845

C 2.49549706 1.11888271 10.34763990

C 4.05016911 -1.84269605 0.63529363

C 4.00417324 -1.11283549 1.87856697

C 3.99769658 -1.81192379 3.13083498

C 3.32736638 0.24730496 4.29895498

C 3.89335616 -1.06117404 4.34867397

C 2.72497818 0.81945880 5.46072050

C 3.14321872 0.33801236 6.73409151

C 1.36993643 1.68380858 7.79178877

C 2.59109582 0.94512927 7.89759260

C 0.61272756 1.93498065 8.97175279

C 1.27623258 1.85513886 10.24559787

C 3.92431637 -1.78142856 -4.33645856

C 3.91752890 -1.09889782 -3.08161926

C 4.01576442 -1.83805907 -1.85138891

C 3.53784709 0.21841159 -0.60850260

C 4.02417392 -1.12752523 -0.60891944

C 3.12036394 0.79763738 0.62113479

C 3.52033099 0.22255825 1.86152954

C 1.70075225 1.46852042 2.97770300

C 2.94926188 0.78086676 3.04683592

C 0.85885098 1.63118071 4.08734099

C 1.49412701 1.52379061 5.35859231

C -0.69389184 1.69830470 6.39993962

C 0.72368464 1.77843144 6.52424276

C -1.41792926 1.51190426 7.62460612

C -0.80692704 1.84402921 8.88600181

C -2.60669920 0.70293637 10.11493825

C -1.53666309 1.64639064 10.11394920

C 3.69740183 -1.55299929 -9.26670746

C 3.66275963 -0.90272275 -8.00054343

C 3.80008327 -1.68116218 -6.80451646

C 3.18589088 0.27906413 -5.47595789

C 3.78482514 -1.01724883 -5.53862102

C 2.76866931 0.77557872 -4.21569346

C 3.35210329 0.20680137 -3.04684742

C 1.72497421 1.45227681 -1.74122827

C 2.97080861 0.75707888 -1.79677988

C 1.10360415 1.65631194 -0.50704848

C 1.93841234 1.56356401 0.61308337

C -0.28591129 1.46812529 1.37898316

C 1.08082089 1.63326615 1.73228426

C -1.11311006 1.17735393 2.55572604

C -0.55410326 1.51367587 3.87572745

C -2.34324174 0.40611606 5.11814870

C -1.28124780 1.34194736 5.11708380

C -2.76850191 -0.13053454 6.40215576

C -2.49103885 0.56575311 7.62566752

C -3.16328751 -1.39462284 8.97542784

C -2.89542384 0.00236137 8.88803331

C -3.16589273 -2.06164631 10.24983702

C 2.99425906 0.54125488 -10.37134071

C 3.57655936 -0.76075862 -10.45849266

C 2.54160639 1.00706104 -9.09484669

C 3.06602684 0.39717523 -7.92173981

C 1.41317594 1.60672744 -6.55579216

C 2.62885120 0.86948248 -6.64945644

C 0.76351760 1.72307111 -5.28706519

C 1.52725302 1.48341402 -4.12589362

C -0.52409369 1.46965284 -2.65319580

C 0.88405704 1.58867025 -2.85932288

C -1.08824924 1.15325343 -1.31727337

C -0.28431137 1.47444225 -0.15805549

C -2.59793044 -0.57065251 1.38120374

C -2.20524156 0.21633363 2.55652656

C -2.89936258 -1.68210039 4.09127412

C -2.60673150 -0.29507834 3.87782033

C -2.87041987 -2.32440104 5.36317828

C -3.02519065 -1.52684543 6.52812999

C -2.43024923 -3.48245842 7.90258145

C -3.01034598 -2.17856268 7.79619487

C -2.01995977 -3.96350122 9.17880476

C -2.58817054 -3.36353961 10.35246820

C 0.56898714 2.00401673 -10.19649832

C 1.31883543 1.75116153 -9.00303402

C -0.76408549 1.77116992 -7.66014622

C 0.66424907 1.85844511 -7.74131113

C -1.37638759 1.43949009 -6.39737190

C -0.66067715 1.64445506 -5.16510746

C -2.32404705 0.36068240 -3.89055130

C -1.25185427 1.30574694 -3.89156126

C -2.57619055 -0.33929572 -2.65100259

C -2.18966352 0.18252200 -1.31626772

C -2.96181869 -1.92832797 -0.50260976

C -2.60715137 -0.57380617 -0.15584169

C -2.97234453 -2.76658033 0.61871252

C -2.93227093 -1.90538514 1.73664859

C -2.31823017 -3.86383739 3.05267490

C -2.84453278 -2.53929065 2.98282517

C -1.83458332 -4.30430675 4.30456564

C -2.32535730 -3.63356740 5.46602178

C -0.69141457 -4.86206199 6.81610060

C -1.89839650 -4.10739580 6.73917448

C -0.04734418 -4.96018548 8.08381888

C -0.81034361 -4.71218633 9.27077898

C 1.24861341 -4.72126550 10.60999532

C -0.17300130 -4.83359151 10.55139306

C -0.85395847 1.89652840 -10.13458392

C -2.55043415 0.60819378 -8.87958168

C -1.47473326 1.55653452 -8.88059599

C -2.85138683 -0.06876871 -7.65814058

C -2.44423743 0.49830455 -6.39630498

C -2.99248190 -1.58800112 -5.28303132

C -2.73591572 -0.18476462 -5.16293909

C -2.84841802 -2.37437354 -4.12109144

C -2.87074717 -1.72169475 -2.85521847

C -2.30432824 -3.89459888 -1.79098183

C -2.83885653 -2.57187723 -1.73620642

C -1.83909646 -4.52300140 -0.60249307

C -2.36005233 -4.03493958 0.62731253

C -0.57297774 -5.14874898 1.88356781

C -1.83747821 -4.50186645 1.86750440

C 0.12301325 -5.22849585 3.13498498

C -0.60727686 -5.02973476 4.35343881

C 1.47779532 -4.96447822 5.64325812

C 0.06296482 -5.10099518 5.61420486

C 2.09026815 -4.62752579 6.90270447

C 1.37846838 -4.84961337 8.13055851

C 3.04595321 -3.55345874 9.38331587

C 1.97685711 -4.49667667 9.38437253

C 3.36089330 -2.85796659 10.60794672

C -3.25168099 -1.36452633 -10.19295828

C -2.96673503 0.03380909 -10.13266147

C -3.09373593 -2.13896444 -8.99878209

C -3.11720710 -1.47501717 -7.73751370

C -2.37981534 -3.54646584 -6.64425957

C -2.95986356 -2.24833774 -6.55124150

C -1.86178079 -4.17135644 -5.47062336

C -2.30082409 -3.69435089 -4.21023686

C -0.58235550 -5.06641378 -3.07676193

C -1.80738291 -4.34266707 -3.04125220

C 0.14029375 -5.25493561 -1.84717685

C -0.56421553 -5.17329119 -0.60390863

C 1.55493818 -5.07155941 0.64342737

C 0.14378489 -5.28689489 0.63954741

C 2.19927748 -4.75626516 1.89758727

C 1.53989861 -5.04342079 3.14843056

C 3.23945284 -3.77555949 4.39896219

C 2.16766849 -4.72067437 4.40001787

C 3.56889450 -3.12021682 5.64119089

C 3.15930457 -3.68453651 6.90164882

C 3.46967525 -3.00486616 8.12857154

C -2.10355442 -3.95343746 -10.36637359

C -2.50807940 -3.44509250 -9.08987881

C -0.75148323 -4.79410733 -7.99602646

C -1.96699520 -4.04021893 -7.91665042

C 0.00512732 -5.02621882 -6.80054388

C -0.65005656 -4.92686490 -5.53388338

C 1.51669377 -5.02240154 -4.35378344

C 0.09226764 -5.15947546 -4.33236857

C 2.19198688 -4.75559559 -3.10991587

C 1.55580343 -5.06798066 -1.85376458

C 3.28378128 -3.82889869 -0.60636384

C 2.20874602 -4.77660542 -0.60518181

C 3.65997759 -3.21583529 0.64112636

C 3.26790633 -3.81417918 1.89644780

C 3.63677016 -3.19451837 3.14620911

C -0.88436976 -4.69353283 -10.45404329

C 1.31633681 -4.80524568 -9.31404568

C -0.11210963 -4.91089981 -9.26286016

C 2.03447900 -4.58787875 -8.09557134

C 1.42515853 -4.91978089 -6.83938871

C 3.20601028 -3.75015118 -5.60660969

C 2.13389963 -4.69501502 -5.60551748

C 3.60981603 -3.17748204 -4.35594971

C 3.26182876 -3.81247801 -3.11100337

C 3.65306260 -3.21900179 -1.85604053

C 3.00044840 -3.52378454 -10.57028556

C 1.92906147 -4.46812895 -10.56917475

C 3.41351670 -2.95702454 -9.31623593

C 3.10918416 -3.64093173 -8.09672146

C 3.51654056 -3.07669659 -6.84156606

C -2.69075316 -3.37647452 -11.54476955

C -3.27229343 -2.05371894 -11.45657252

C -0.23753362 -4.84887010 -11.72881283

C 1.20295066 -4.72608521 -11.78746695

C 3.34593118 -2.83690110 -11.78966747

C 3.64831570 -1.42308618 -11.73274377

C 2.49376421 1.19491819 -11.54963840

C 1.25414895 1.93728594 -11.46071847

C -1.59487900 1.69997477 -11.35424574

C -2.68003327 0.74320197 -11.35324971

C -2.23538109 -3.84376787 -12.79917939

C -0.99906067 -4.57975592 -12.89129836

C 1.79426593 -4.32799535 -13.01035456

C 2.87535238 -3.37488633 -13.01147863

C 3.47496979 -0.63488797 -12.89568164

C 2.89929364 0.68376617 -12.80401064

C -0.94764330 1.99094143 -12.58024888

C 0.48719158 2.11913009 -12.63407718

C -3.35784774 -1.27111739 -12.63062603

C -3.05095240 0.13643235 -12.57832062

C -0.37980168 -4.24636734 -14.15096047

C 1.01560061 -4.11669074 -14.20789720

C -2.93791647 -1.77533314 -13.91648627

C -2.38026446 -3.05997931 -14.00181419

C -2.43560926 0.49722694 -13.83336284

C -1.38393072 1.42457143 -13.83437085

C 0.93363320 1.63847876 -13.91997813

C 2.13836996 0.92449482 -14.00589896

C 2.76187476 -2.57700416 -14.20962712

C 3.06524244 -1.20875703 -14.15427960

C -1.22849636 -3.29978071 -14.84670344

C -2.36075596 -0.68299517 -14.67146040

C -0.22313622 1.20189100 -14.67337718

C 2.23127989 -0.24904057 -14.84977689

C 1.60927996 -3.03073943 -14.96094558

C -0.14244458 0.05253324 -15.48346782

C 1.10531050 -0.68494205 -15.57391093

C 0.78868663 -2.10095730 -15.62900012

C -0.65578572 -2.23783647 -15.57227879

C -1.23130098 -0.90761989 -15.48245890

C -0.97101948 -4.63640315 11.73168384

C -2.20749661 -3.89220635 11.63014691

C 1.87598250 -4.39514692 11.86280822

C 2.96036103 -3.43872205 11.86164724

C 3.55639822 -0.64435509 11.72737578

C 2.97351902 0.67578901 11.62524783

C 0.51806229 2.14796662 11.42716755

C -0.92186085 2.02944588 11.35990551

C -3.06258963 0.14224272 11.36187695

C -3.36038307 -1.27147938 11.43070104

C -0.31373006 -4.68545002 12.98528983

C 1.11466703 -4.55733747 13.05025755

C 3.21788637 -2.70263151 13.04808567

C 3.52391958 -1.30166570 12.98151740

C 2.40151036 1.26956022 12.77749214

C 1.17070624 2.00217921 12.67814887

C -2.69980714 0.80484441 12.56284340

C -1.62351491 1.75366591 12.56185365

C -2.72374633 -3.24894512 12.78210304

C -3.29634458 -1.93610823 12.68207753

C 1.43738132 -3.86051088 14.27598720

C 2.48761554 -2.93447540 14.27487645

C -2.07856636 -3.36122825 14.07031964

C -0.87648282 -4.07439904 14.16970847

C -3.00417160 -1.23084245 13.90914174

C -2.70105968 0.13494836 13.84628042

C -0.95745252 1.67211418 13.84474431

C 0.43566136 1.80158442 13.90616611

C 2.98981261 -0.66579563 14.16608269

C 2.43325386 0.61628691 14.06622125

C 0.20684052 -3.55886064 14.97909903

C -2.24594934 -2.10871800 14.77714024

C -1.62352146 0.67095584 14.65136847

C 1.21241202 0.93996402 14.77409675

C 2.34352259 -1.67524604 14.97713030

C -0.87308616 -0.17943743 15.48969513

C 0.57051673 -0.04286427 15.55329908

C 1.14556190 -1.37202201 15.65575102

C 0.05701887 -2.33168517 15.65674837

C -1.18960092 -1.59448731 15.55483511

H -4.64026707 3.28294267 0.48726472

C -3.63357870 2.87020754 0.52423905

H -3.36598506 2.47312983 -0.45460166

H -2.92852961 3.65177841 0.80235469

H -3.59441287 2.06838531 1.26103176

Table S17. The coordinate of the structures for “c” defective nanotube adsorbed with NH_3_ for physical adsorption.

C 3.66861018 -0.38840944 10.59508133

C 3.90525228 -0.65395358 5.65716767

C 3.75519495 0.12280750 6.85934469

C 3.77918528 -0.52800774 8.12726238

C 3.01631461 1.54626184 9.22285210

C 3.62091968 0.25849677 9.31437279

C 2.48559721 2.17943418 10.39668228

C 4.07654799 -0.75417398 0.68149495

C 4.02257853 -0.02687019 1.92596474

C 4.02314495 -0.72773516 3.17731082

C 3.33069133 1.32252381 4.34796193

C 3.91043758 0.02004197 4.39630118

C 2.72142275 1.88673735 5.50999445

C 3.14415093 1.40850242 6.78292645

C 1.35538865 2.73330375 7.84097879

C 2.58492354 2.00865969 7.94668359

C 0.59442102 2.97442289 9.02050213

C 1.25792094 2.90157975 10.29459929

C 3.95045924 -0.68549931 -4.29085011

C 3.93662194 -0.00548138 -3.03471373

C 4.04249359 -0.74563637 -1.80553514

C 3.54341235 1.30365835 -0.55942225

C 4.04341434 -0.03727998 -0.56176663

C 3.11963445 1.87651465 0.67103309

C 3.52536591 1.30365574 1.91058667

C 1.69142820 2.52730793 3.02774675

C 2.94763359 1.85384656 3.09647122

C 0.84688336 2.67881501 4.13687922

C 1.48280528 2.57731009 5.40804475

C -0.70764966 2.72495572 6.44834609

C 0.70876127 2.82161764 6.57341989

C -1.43043966 2.52905703 7.67207565

C -0.82396048 2.86687383 8.93384798

C -2.61217845 1.70515572 10.16040298

C -1.55252391 2.66024159 10.16082727

C 3.72033346 -0.45078857 -9.22102027

C 3.67903003 0.19695723 -7.95376055

C 3.82477638 -0.58197188 -6.75885979

C 3.19048195 1.36936442 -5.42714444

C 3.80285428 0.07936122 -5.49172580

C 2.76824259 1.85908225 -4.16594162

C 3.35790145 1.29432307 -2.99805527

C 1.71738430 2.51953514 -1.69063955

C 2.97085805 1.83820848 -1.74700888

C 1.09364530 2.71426426 -0.45634943

C 1.92929567 2.62946703 0.66373110

C -0.29443556 2.50699297 1.42819252

C 1.07028632 2.68694899 1.78246067

C -1.11936774 2.20496902 2.60408076

C -0.56457243 2.54549187 3.92449863

C -2.34202803 1.41633051 5.16454926

C -1.29032749 2.36358355 5.16502320

C -2.76249112 0.87357524 6.44741452

C -2.49328264 1.57147619 7.67159617

C -3.14593512 -0.39721217 9.01882140

C -2.89256812 1.00251518 8.93294529

C -3.14219243 -1.06526860 10.29264902

C 2.99447818 1.63748791 -10.32238271

C 3.59068295 0.34193043 -10.41160531

C 2.53723564 2.09637253 -9.04501001

C 3.06857825 1.49035639 -7.87293840

C 1.40299848 2.67939020 -6.50475208

C 2.62679379 1.95578490 -6.59966057

C 0.75236912 2.78600366 -5.23567329

C 1.51902223 2.55300558 -4.07500572

C -0.53195052 2.51274547 -2.60248111

C 0.87476382 2.64845793 -2.80832126

C -1.09265809 2.18753646 -1.26745332

C -0.29239999 2.51628523 -0.10776967

C -2.58491245 0.44217787 1.42720627

C -2.20074643 1.23181237 2.60348833

C -2.87561783 -0.67624275 4.13512573

C -2.59756537 0.71410039 3.92350385

C -2.84045177 -1.31981596 5.40607177

C -3.00470559 -0.52539755 6.57176097

C -2.38932101 -2.47590477 7.94437254

C -2.98383263 -1.17839956 7.83901300

C -1.97437884 -2.95322402 9.22049915

C -2.55012857 -2.36075283 10.39439336

C 0.55331284 3.07281783 -10.14483044

C 1.30630445 2.82677996 -8.95188483

C -0.77637385 2.82067723 -7.60859773

C 0.65089728 2.92450776 -7.68977304

C -1.38440466 2.47974667 -6.34622846

C -0.67078926 2.69063827 -5.11383599

C -2.31944232 1.38595461 -3.84146541

C -1.25782941 2.34274234 -3.84102006

C -2.56429561 0.68122976 -2.60325328

C -2.18390842 1.20542308 -1.26773107

C -2.93304356 -0.91606084 -0.45809697

C -2.59343340 0.44186416 -0.10872659

C -2.93523271 -1.75650248 0.66152099

C -2.90497034 -0.89666753 1.78072320

C -2.26954555 -2.85001572 3.09411476

C -2.81077068 -1.53143269 3.02571242

C -1.78126389 -3.28630778 4.34563045

C -2.28049683 -2.62277178 5.50767246

C -0.63354296 -3.83392340 6.85732785

C -1.84918422 -3.09325749 6.78063145

C 0.01062970 -3.92578765 8.12555356

C -0.75594659 -3.68757425 9.31233353

C 1.30205009 -3.67388705 10.65270948

C -0.11812191 -3.80253591 10.59329601

C -0.86835016 2.94911239 -10.08276475

C -2.55004590 1.63972784 -8.82959757

C -1.48496878 2.60010958 -8.82922611

C -2.84300772 0.95738305 -7.60930927

C -2.44159375 1.52663302 -6.34664880

C -2.96718031 -0.56739519 -5.23704446

C -2.72556325 0.83827847 -5.11461426

C -2.81472117 -1.35433365 -4.07659874

C -2.84385092 -0.70400122 -2.80977279

C -2.25405614 -2.87290148 -1.74912018

C -2.80269202 -1.55596132 -1.69254363

C -1.78187620 -3.49799698 -0.56170131

C -2.30850152 -3.01779827 0.66869291

C -0.50939062 -4.11329915 1.92401329

C -1.78110902 -3.48081427 1.90826845

C 0.18694285 -4.18706047 3.17566501

C -0.54590185 -3.99785880 4.39424819

C 1.53751066 -3.91106200 5.68534478

C 0.12425643 -4.06302688 5.65549368

C 2.14524993 -3.56846895 6.94569941

C 1.43509803 -3.79913137 8.17311021

C 3.08720594 -2.48553550 9.42814903

C 2.02846657 -3.44033709 9.42768824

C 3.39383429 -1.78766497 10.65365605

C -3.23011209 -0.33854041 -10.14606359

C -2.96030622 1.06272065 -10.08343873

C -3.06335613 -1.11316790 -8.95328985

C -3.09350459 -0.45152844 -7.69105772

C -2.33370428 -2.51691967 -6.60137434

C -2.92752722 -1.22520255 -6.50622392

C -1.80880931 -3.13832543 -5.42890135

C -2.25293318 -2.66827309 -4.16780521

C -0.51925704 -4.02331436 -3.03649208

C -1.75233344 -3.31341620 -3.00005936

C 0.20538498 -4.20583410 -1.80713816

C -0.49990382 -4.13410971 -0.56370778

C 1.61775856 -4.01100817 0.68435139

C 0.20908053 -4.24188543 0.67982654

C 2.25820485 -3.69050610 1.93938651

C 1.60174431 -3.98664761 3.18989237

C 3.28634658 -2.70131014 4.44284286

C 2.22523362 -3.65849230 4.44240430

C 3.60778228 -2.04390657 5.68622384

C 3.20379434 -2.61370745 6.94614578

C 3.50586821 -1.93174249 8.17401790

C -2.05393279 -2.91457068 -10.32398800

C -2.46359161 -2.41272900 -9.04661656

C -0.69216637 -3.74440454 -7.95520560

C -1.91577199 -3.00391975 -7.87457644

C 0.06721386 -3.97017098 -6.76021184

C -0.58885940 -3.88054527 -5.49335397

C 1.57903703 -3.95347104 -4.31341605

C 0.15624321 -4.10679792 -4.29232815

C 2.25130865 -3.68129149 -3.06897399

C 1.61879583 -4.00308896 -1.81325580

C 3.33238131 -2.74661135 -0.56351173

C 2.26825078 -3.70660793 -0.56396645

C 3.70134653 -2.13147721 0.68526518

C 3.31600747 -2.73619806 1.93982600

C 3.67748863 -2.11426967 3.19077381

C -0.82656357 -3.64084733 -10.41293194

C 1.37544845 -3.72948646 -9.27331221

C -0.05170363 -3.85170124 -9.22223965

C 2.09107600 -3.50589050 -8.05456687

C 1.48589251 -3.84723403 -6.79887155

C 3.25346724 -2.65955314 -5.56423990

C 2.19217547 -3.61663982 -5.56467190

C 3.65095394 -2.08480784 -4.31254065

C 3.31032887 -2.72601888 -3.06852300

C 3.69480103 -2.13053658 -1.81235283

C 3.04487220 -2.42710797 -10.52754101

C 1.98412559 -3.38337819 -10.52785386

C 3.45159560 -1.85775451 -9.27266268

C 3.15487589 -2.54682850 -8.05419426

C 3.55625610 -1.98042085 -6.79810216

C -2.64770209 -2.34211550 -11.50133666

C -3.24361821 -1.02594095 -11.41077620

C -0.17816996 -3.78671686 -11.68799229

C 1.26081348 -3.64755030 -11.74650395

C 3.38267359 -1.73462848 -11.74593267

C 3.66938434 -0.31764031 -11.68687915

C 2.48655331 2.28728229 -11.49972702

C 1.23883280 3.01570827 -11.40934727

C -1.60726618 2.74598256 -11.30262585

C -2.68166334 1.77711010 -11.30294913

C -2.18735121 -2.80229134 -12.75655821

C -0.94282817 -3.52420313 -12.84995265

C 1.84758870 -3.24113672 -12.96877413

C 2.91809599 -2.27598694 -12.96850794

C 3.48721609 0.47024698 -12.84867795

C 2.89720028 1.78241255 -12.75499204

C -0.96371851 3.04613442 -12.52827992

C 0.46958783 3.19061889 -12.58228134

C -3.33787006 -0.24242825 -12.58348304

C -3.04619138 1.16824582 -12.52888187

C -0.32740701 -3.18192944 -14.10906370

C 1.06648240 -3.03669611 -14.16592414

C -2.91273798 -0.73989015 -13.87026034

C -2.34098031 -2.01813897 -13.95785246

C -2.43526577 1.53789973 -13.78347691

C -1.39397126 2.47690793 -13.78318124

C 0.92095859 2.71682639 -13.86904395

C 2.13347559 2.01639684 -13.95642518

C 2.79561035 -1.47770843 -14.16552244

C 3.08374886 -0.10625227 -14.10813119

C -1.18666533 -2.24372169 -14.80320885

C -2.34768623 0.35994313 -14.62351253

C -0.23103482 2.26849963 -14.62286277

C 2.23917055 0.84528889 -14.80214057

C 1.64800335 -1.94308192 -14.91738553

C -0.13787651 1.12136759 -15.43477517

C 1.11793590 0.39792506 -15.52661465

C 0.81702998 -1.02143103 -15.58386595

C -0.62584388 -1.17440930 -15.52717413

C -1.21602229 0.14919393 -15.43510947

C -0.91902496 -3.61560354 11.77337686

C -2.16412023 -2.88589829 11.67194516

C 1.92509121 -3.34195172 11.90613820

C 2.99889744 -2.37368910 11.90663382

C 3.56488294 0.42701602 11.77548854

C 2.96756054 1.74083129 11.67429199

C 0.49527122 3.18485322 11.47560323

C -0.94314545 3.04979997 11.40726289

C -3.06311409 1.13877113 11.40645973

C -3.34584230 -0.27810984 11.47400031

C -0.26182555 -3.65809518 13.02725430

C 1.16497819 -3.51375420 13.09300983

C 3.24764063 -1.63593974 13.09396217

C 3.53848623 -0.23162480 13.02903787

C 2.38759783 2.32705679 12.82650306

C 1.14859761 3.04574615 12.72684453

C -2.70850555 1.80473720 12.60795757

C -1.64278965 2.76552867 12.60832817

C -2.68806695 -2.24933374 12.82426935

C -3.27518143 -0.94283297 12.72497573

C 1.47918121 -2.81451462 14.31957551

C 2.51910341 -1.87693043 14.32002731

C -2.04213631 -2.35514064 14.11270542

C -0.83200700 -3.05453174 14.21197518

C -2.99131001 -0.23507980 13.95261851

C -2.70332885 1.13404292 13.89092702

C -0.97681152 2.69066535 13.89156805

C 0.41470153 2.83585411 13.95397488

C 2.99631258 0.39721125 14.21373030

C 2.42551790 1.67306133 14.11468970

C 0.24493516 -2.52745225 15.02239422

C -2.22380102 -1.10507153 14.82041412

C -1.63231953 1.68157129 14.69699040

C 1.20045397 1.98236425 14.82182721

C 2.36052451 -0.62000152 15.02330695

C -0.87298907 0.83902892 15.53508953

C 0.56896566 0.99176318 15.59970889

C 1.15882158 -0.33090643 15.70149288

C 0.08103403 -1.30266641 15.70109504

C -1.17370205 -0.57950275 15.59900960

H -3.01212868 4.50322686 0.58124126

N -3.68781642 3.74338946 0.67966011

H -3.59108363 3.09258339 -0.10296568

H -3.52306018 3.24810893 1.55891197

Table S18. The coordinate of the structures for “c” defective nanotube adsorbed with H_2_O for physical adsorption.

C 5.39607927 -1.71535792 10.49392624

C 5.63201007 -1.97766961 5.55666131

C 5.48174397 -1.20133721 6.75888915

C 5.50583106 -1.85294457 8.02639592

C 4.74452631 0.22108332 9.12303329

C 5.34814812 -1.06729102 9.21387633

C 4.21459915 0.85349151 10.29759034

C 5.80613038 -2.07774925 0.58199857

C 5.75077971 -1.35013730 1.82619346

C 5.75076504 -2.05109680 3.07734336

C 5.05759062 -0.00073168 4.24791907

C 5.63725562 -1.30330548 4.29614557

C 4.44904686 0.56382084 5.41008449

C 4.87149678 0.08473442 6.68290445

C 3.08436241 1.41066841 7.74194606

C 4.31325721 0.68474303 7.84723514

C 2.32377086 1.65097067 8.92190991

C 2.98732673 1.57646451 10.19607188

C 5.68540108 -2.01063762 -4.39012812

C 5.66911052 -1.33017981 -3.13417334

C 5.77447008 -2.06983856 -1.90476082

C 5.27286799 -0.02037276 -0.65937914

C 5.77350540 -1.36108004 -0.66128557

C 4.84834770 0.55281592 0.57074676

C 5.25316348 -0.01980154 1.81065873

C 3.41970724 1.20579419 2.92721408

C 4.67505043 0.53068173 2.99625644

C 2.57595091 1.35904020 4.03687414

C 3.21143971 1.25646837 5.30829627

C 1.02136190 1.40365067 6.34936204

C 2.43772709 1.50051423 6.47425477

C 0.29902230 1.20600051 7.57318252

C 0.90532613 1.54317819 8.83529775

C -0.88207560 0.37959793 10.06123229

C 0.17718373 1.33508814 10.06238430

C 5.45938920 -1.77737224 -9.32109031

C 5.41625598 -1.12972448 -8.05379084

C 5.56224175 -1.90812442 -6.85851204

C 4.92371135 0.04265614 -5.52738364

C 5.53805603 -1.24648238 -5.59142687

C 4.50013269 0.53280612 -4.26675931

C 5.08899276 -0.03096787 -3.09808211

C 3.44783523 1.19576366 -1.79184010

C 4.70091484 0.51354629 -1.84746393

C 2.82362803 1.39155248 -0.55771392

C 3.65861572 1.30671472 0.56279599

C 1.43447596 1.18745402 1.32619547

C 2.79936201 1.36556407 1.68132666

C 0.60921325 0.88619421 2.50376845

C 1.16422720 1.22641883 3.82489466

C -0.61132101 0.09359798 5.06460728

C 0.43883891 1.04268437 5.06547958

C -1.03173863 -0.44997479 6.34730932

C -0.76302179 0.24739098 7.57207809

C -1.41605230 -1.72207645 8.91803160

C -1.16238962 -0.32219203 8.83312241

C -1.41284527 -2.39095800 10.19151445

C 4.73178467 0.31005539 -10.42298580

C 5.32947478 -0.98483352 -10.51184506

C 4.27304461 0.76831958 -9.14596394

C 4.80381827 0.16275411 -7.97341946

C 3.13693670 1.35191514 -6.60675431

C 4.36062632 0.62811695 -6.70062712

C 2.48567952 1.46034312 -5.33817724

C 3.25124055 1.22749914 -4.17671563

C 1.19939170 1.19116129 -2.70532232

C 2.60632197 1.32504172 -2.91044766

C 0.63755080 0.86719455 -1.37038400

C 1.43704391 1.19605907 -0.20970791

C -0.85379857 -0.87885627 1.32505826

C -0.46895985 -0.09053388 2.50378257

C -1.14456202 -1.99901430 4.03353256

C -0.86631317 -0.60831572 3.82323062

C -1.11074767 -2.64288976 5.30418667

C -1.27446889 -1.84892243 6.47057523

C -0.66120310 -3.80075193 7.84233604

C -1.25446357 -2.50251697 7.73762039

C -0.24685916 -4.27922905 9.11818921

C -0.82183025 -3.68696068 10.29253714

C 2.29019320 1.74566373 -10.24733896

C 3.04203430 1.49870611 -9.05386439

C 0.95842717 1.49579631 -7.71213696

C 2.38587538 1.59729675 -7.79232915

C 0.34916070 1.15650569 -6.44992799

C 1.06238235 1.36707902 -5.21719713

C -0.58750101 0.06447785 -3.94503456

C 0.47434598 1.02084495 -3.94432152

C -0.83237951 -0.63986807 -2.70650413

C -0.45167620 -0.11553755 -1.37150721

C -1.19974002 -2.23731772 -0.56063524

C -0.86238801 -0.87841092 -0.21137240

C -1.20356304 -3.07787920 0.55878850

C -1.17227171 -2.21860039 1.67804765

C -0.53954244 -4.17250900 2.99128912

C -1.07905003 -2.85326084 2.92337668

C -0.05286203 -4.60998680 4.24308415

C -0.55192350 -3.94652458 5.40523028

C 1.09377466 -5.15919834 6.75476346

C -0.12146470 -4.41788869 6.67818056

C 1.73783834 -5.25181106 8.02298070

C 0.97135377 -5.01417239 9.20979507

C 3.02960696 -5.00137807 10.54998145

C 1.60944017 -5.13002913 10.49054285

C 0.86838195 1.62352522 -10.18643854

C -0.81549821 0.31635937 -8.93399356

C 0.25044519 1.27577074 -8.93322138

C -1.10947017 -0.36527735 -7.71345869

C -0.70895056 0.20441805 -6.45059840

C -1.23340873 -1.88943105 -5.34052743

C -0.99328033 -0.48348091 -5.21825773

C -1.08051497 -2.67589200 -4.17974521

C -1.11074766 -2.02544272 -2.91291278

C -0.52013545 -4.19376010 -1.85201968

C -1.06903303 -2.87700543 -1.79537720

C -0.04966722 -4.81974250 -0.66437358

C -0.57706261 -4.33968875 0.56566826

C 1.22032586 -5.43683533 1.82144279

C -0.05106481 -4.80351340 1.80539970

C 1.91574603 -5.51123440 3.07351613

C 1.18217604 -5.32239155 4.29176894

C 3.26505155 -5.23564219 5.58334008

C 1.85178118 -5.38818313 5.55309052

C 3.87248459 -4.89324951 6.84369680

C 3.16230205 -5.12489827 8.07075041

C 4.81439462 -3.81168524 9.32614648

C 3.75586145 -4.76686990 9.32524033

C 5.12123960 -3.11465344 10.55177633

C -1.49324402 -1.66260504 -10.25031601

C -1.22455169 -0.26116126 -10.18811264

C -1.32657796 -2.43646847 -9.05700347

C -1.35857190 -1.77443438 -7.79495226

C -0.59645760 -3.83822173 -6.70429643

C -1.19230506 -2.54741558 -6.60967566

C -0.07204208 -4.45911824 -5.53150318

C -0.51727341 -3.98921551 -4.27072504

C 1.21487335 -5.34512646 -3.13857404

C -0.01770986 -4.63437707 -3.10270044

C 1.93823728 -5.52885256 -1.90882941

C 1.23182898 -5.45684616 -0.66603585

C 3.34848568 -5.33523265 0.58325219

C 1.93973802 -5.56566730 0.57789918

C 3.98807076 -5.01476691 1.83853799

C 3.33055129 -5.31092933 3.08837042

C 5.01412929 -4.02514019 4.34205499

C 3.95334536 -4.98267247 4.34104568

C 5.33497150 -3.36778356 5.58532198

C 4.93078521 -3.93824729 6.84465350

C 5.23270714 -3.25674520 8.07261600

C -0.31418348 -4.23757177 -10.42628710

C -0.72491093 -3.73520096 -9.14953280

C 1.04638429 -5.06577208 -8.05687185

C -0.17724283 -4.32529837 -7.97701526

C 1.80479051 -5.29195130 -6.86149946

C 1.14778826 -5.20150510 -5.59521777

C 3.31455274 -5.27763537 -4.41406853

C 1.89153266 -5.42897067 -4.39377260

C 3.98586286 -5.00589590 -3.16911344

C 3.35177177 -5.32709864 -1.91405584

C 5.06435472 -4.07090906 -0.66324616

C 4.00027363 -5.03093751 -0.66435233

C 5.43184724 -3.45526120 0.58560991

C 5.04554137 -4.06011014 1.83962600

C 5.40587417 -3.43779355 3.09049462

C 0.91317834 -4.96400933 -10.51444277

C 3.11483703 -5.05464002 -9.37402299

C 1.68741415 -5.17462000 -9.32341862

C 3.83025391 -4.83175729 -8.15501919

C 3.22368380 -5.17163849 -6.89952221

C 4.99146762 -3.98564696 -5.66388723

C 3.92936095 -4.94190924 -5.66484580

C 5.38741076 -3.41032767 -4.41190658

C 5.04527342 -4.05110641 -3.16805431

C 5.42807347 -3.45504594 -1.91169587

C 4.78574009 -3.75399431 -10.62798995

C 3.72439749 -4.70963754 -10.62851841

C 5.19210725 -3.18466446 -9.37285289

C 4.89499594 -3.87363115 -8.15430478

C 5.29526987 -3.30689935 -6.89784066

C -0.90783627 -3.66634318 -11.60418555

C -1.50494705 -2.35061477 -11.51476021

C 1.56186074 -5.11148088 -11.78912632

C 3.00104445 -4.97344694 -11.84729010

C 5.12339832 -3.06135825 -11.84644325

C 5.40941223 -1.64421557 -11.78717995

C 4.22450386 0.95999414 -11.60055635

C 2.97670973 1.68846490 -11.51124313

C 0.13033861 1.42073059 -11.40690162

C -0.94462581 0.45245358 -11.40787098

C -0.44683787 -4.12729888 -12.85882331

C 0.79766780 -4.84931585 -12.95136586

C 3.58817602 -4.56752737 -13.06960925

C 4.65879420 -3.60251293 -13.06913791

C 5.22709808 -0.85630023 -12.94898679

C 4.63636787 0.45555180 -12.85554694

C 0.77520745 1.72006192 -12.63203780

C 2.20860060 1.86396490 -12.68480166

C -1.59883473 -1.56792861 -12.68803269

C -1.30783379 -0.15703132 -12.63394566

C 1.41347371 -4.50798879 -14.21049677

C 2.80737783 -4.36307132 -14.26698852

C -1.17252793 -2.06601815 -13.97421857

C -0.60025164 -3.34412509 -14.06071396

C -0.69563290 0.21193511 -13.88816246

C 0.34587223 1.15072036 -13.88720699

C 2.66094163 1.39016033 -13.97120084

C 3.87355616 0.68972033 -14.05758418

C 4.53649497 -2.80424275 -14.26626254

C 4.82430338 -1.43275194 -14.20864998

C 0.55447948 -3.57015247 -14.90540503

C -0.60716758 -0.96642996 -14.72765181

C 1.50945107 0.94192895 -14.72590222

C 3.97994288 -0.48137148 -14.90321325

C 3.38911792 -3.26963585 -15.01850537

C 1.60328364 -0.20532912 -15.53753389

C 2.85918257 -0.92875412 -15.62841079

C 2.55839046 -2.34814779 -15.68556343

C 1.11551662 -2.50114087 -15.62962414

C 0.52512017 -1.17751132 -15.53835562

C 0.80885946 -4.94355830 11.67088246

C -0.43582118 -4.21305005 11.56978355

C 3.65292654 -4.67021170 11.80342662

C 4.72666444 -3.70180713 11.80429101

C 5.29316057 -0.90096432 11.67498972

C 4.69644480 0.41318441 11.57479118

C 2.22504190 1.85843084 11.37768482

C 0.78659171 1.72350310 11.30927502

C -1.33289722 -0.18769152 11.30695052

C -1.61596558 -1.60453859 11.37349398

C 1.46623476 -4.98697651 12.92460763

C 2.89316125 -4.84284655 12.99026390

C 4.97599923 -2.96505423 12.99196481

C 5.26716857 -1.56067210 12.92795726

C 4.11726520 0.99849503 12.72773255

C 2.87843186 1.71767495 12.62877069

C -0.97841941 0.47726232 12.50900587

C 0.08729170 1.43804927 12.51020609

C -0.95908778 -3.57691084 12.72256370

C -1.54568175 -2.27008847 12.62405735

C 3.20783549 -4.14445905 14.21715465

C 4.24786799 -3.20692899 14.21799795

C -0.31328923 -3.68392162 14.01096903

C 0.89654525 -4.38393609 14.10977336

C -1.26167049 -1.56342759 13.85222037

C -0.97341854 -0.19431581 13.79153442

C 0.75326227 1.36197903 13.79337858

C 2.14478373 1.50686242 13.85587368

C 4.72568047 -0.93271182 14.11335393

C 4.15527097 0.34341631 14.01536918

C 1.97381031 -3.85758822 14.92034683

C -0.49446277 -2.43425621 14.71943252

C 0.09771501 0.35232428 14.59805432

C 2.93041128 0.65242792 14.72293987

C 4.08983825 -1.95047655 14.92227384

C 0.85684273 -0.49102994 15.43552711

C 2.29884831 -0.33869534 15.50005564

C 2.88832873 -1.66163579 15.60077136

C 1.81033556 -2.63315431 15.59970614

C 0.55578616 -1.90956048 15.49834011

H -1.24112559 2.14448857 1.38796415

O -1.97706311 2.12626697 0.75742448

H -2.16191950 1.19255241 0.57531260

Table S19. The coordinate of the structures for “d” defective nanotube adsorbed with CH_4_ for physical adsorption.

C 4.96496014 -0.22255157 11.21848395

C 5.02593133 -0.10618750 6.27370326

C 4.70947128 0.52525307 7.52679683

C 4.98684516 -0.16418129 8.74556986

C 3.70063305 1.51266664 10.00124630

C 4.66456932 0.46153005 9.99047835

C 3.04436212 1.85331984 11.23307575

C 4.82741970 -0.01923892 1.33997018

C 4.65534075 0.61415967 2.58884721

C 5.04074857 -0.04018133 3.80811531

C 3.84554980 1.69673478 5.09190074

C 4.77067977 0.59733715 5.05811759

C 3.16541102 2.00887933 6.30991631

C 3.76009460 1.58931090 7.54420476

C 1.69776402 2.18660540 8.73018739

C 3.10206215 1.91039321 8.76776236

C 0.95254560 2.04963621 9.94242076

C 1.64399109 2.12521223 11.20278601

C 4.85135034 0.26996068 -3.52240373

C 4.42962644 0.81876754 -2.27357399

C 4.71331294 0.10846842 -1.07155405

C 3.20356841 1.57374485 0.28145292

C 4.30515865 0.65077636 0.19036952

C 2.66450880 1.79474572 1.61375927

C 3.66566864 1.62264982 2.62588015

C 1.82939470 2.35567881 3.78408070

C 3.21877148 2.08631232 3.89131097

C 1.07940699 2.23495423 4.98832091

C 1.76916183 2.28547901 6.25552442

C -0.25432683 1.48536407 7.41740434

C 1.01723457 2.12876863 7.47168126

C -0.80810124 0.95420895 8.62656174

C -0.32933607 1.42656869 9.89014556

C -1.57598804 -0.35118601 11.05985818

C -0.89993973 0.90506356 11.10464078

C 5.07384380 0.52081450 -8.45958459

C 4.70204213 1.10723222 -7.20711979

C 4.97190814 0.40423749 -5.99147674

C 3.60698280 2.01710307 -4.71121923

C 4.58337443 0.98263112 -4.73484664

C 2.92751247 2.31854336 -3.47986422

C 3.45164357 1.85611390 -2.22427973

C 1.42509105 2.60930153 -1.08161168

C 2.75011286 2.12237793 -0.99260465

C 0.63015395 2.70446126 0.10147646

C 0.08948460 1.41695437 2.42862410

C 1.37930080 2.05283505 2.42288692

C -0.53401682 0.98543648 3.66651386

C -0.15334929 1.51632214 4.93482023

C -1.36853484 -0.27245349 6.11664607

C -0.71127923 0.98862586 6.14681631

C -1.55971964 -1.00092573 7.33808764

C -1.47610317 -0.31221684 8.58669362

C -1.48016696 -2.46212442 9.78210570

C -1.65056767 -1.04522408 9.80243388

C -1.22879073 -3.15926732 11.01425393

C 3.79040158 2.23990243 -9.66915198

C 4.80044308 1.23141722 -9.68081675

C 3.09750099 2.50659829 -8.43640878

C 3.70118855 2.13009580 -7.19274731

C 1.61797478 2.65417738 -6.01348767

C 3.02309436 2.41713198 -5.96510301

C 0.85950099 2.50731184 -4.80794480

C 1.53226680 2.61365643 -3.54683113

C -0.47882089 1.80836002 -2.40894868

C 0.75743539 2.52805295 -2.35587386

C -0.89711318 1.18235548 -1.19301741

C -0.37725622 1.70151427 0.03852008

C -1.30940865 -0.23059519 1.21516676

C -0.62720939 1.01285141 1.24423486

C -1.42223420 -0.96216259 2.42813775

C -1.26248876 -0.24797012 3.65575408

C -1.36007049 -2.39085280 4.85181279

C -1.47485026 -0.96355422 4.87089161

C -1.09159823 -3.06805756 6.08088693

C -1.40770554 -2.41772620 7.31938003

C -0.19383756 -4.16388872 8.54953882

C -1.15250003 -3.10071436 8.54711259

C 0.41055811 -4.53333487 9.78977699

C -0.26228838 -4.20954923 11.01836713

C 0.99962238 2.66758959 -9.73477269

C 1.68977114 2.72863389 -8.47501327

C -0.32064638 1.90085329 -7.32631383

C 0.94092343 2.57335513 -7.26870082

C -0.84843730 1.33428739 -6.12615009

C -0.39219099 1.83632668 -4.86394956

C -1.47479473 -0.04340320 -3.70378187

C -0.91994217 1.27605143 -3.65537136

C -1.55072254 -0.80576217 -2.49811655

C -1.43541661 -0.13253291 -1.23779195

C -1.33593363 -2.31005637 -0.07111567

C -1.51118705 -0.89356568 -0.03113357

C -1.04069465 -3.00632894 1.14222516

C -1.33069201 -2.37428922 2.39563983

C -0.11208686 -4.11886118 3.61168264

C -1.06226842 -3.05548567 3.62291206

C 0.49977473 -4.48585401 4.84967365

C -0.14699137 -4.13309147 6.07909388

C 1.86141420 -4.76683414 7.34561938

C 0.46148341 -4.50224192 7.32451237

C 2.55246515 -4.68968760 8.59550580

C 1.81183826 -4.80246517 9.81341349

C 3.79608858 -4.12436723 11.10591408

C 2.51046598 -4.74305789 11.06892189

C -0.26167887 2.00223614 -9.79211733

C -1.40481390 0.13385815 -8.64568118

C -0.78425181 1.41625851 -8.58768779

C -1.52457804 -0.62738819 -7.43932748

C -1.44644714 0.04126296 -6.17883375

C -1.30072782 -2.12196634 -5.01173382

C -1.54362539 -0.71983356 -4.96422354

C -0.99374671 -2.81762199 -3.79661445

C -1.31976519 -2.21536133 -2.54288451

C -0.04221238 -3.95790247 -1.34613671

C -1.01364293 -2.91594249 -1.32782474

C 0.57208636 -4.36086848 -0.11025253

C -0.07763929 -4.05857057 1.12894221

C 1.94026006 -4.68685225 2.38967725

C 0.53882110 -4.43727297 2.37079479

C 2.63817625 -4.60928193 3.64546574

C 1.90688341 -4.73981101 4.86824537

C 3.86955845 -4.00250951 6.15588200

C 2.59972006 -4.64579271 6.11942694

C 4.36612220 -3.52204647 7.41578149

C 3.83627775 -4.05691262 8.63203319

C 5.00592661 -2.32277076 9.92662152

C 4.33190195 -3.58070666 9.88624474

C 5.14071015 -1.63855641 11.18621587

C -1.26312763 -1.94255664 -9.96435039

C -1.50175244 -0.53608431 -9.91449504

C -0.93273291 -2.62962242 -8.74291809

C -1.28157754 -2.03733862 -7.48798002

C 0.02915155 -3.74382858 -6.29649604

C -0.96485696 -2.72226710 -6.27226424

C 0.64702669 -4.13899725 -5.06120593

C -0.01008856 -3.85552468 -3.81983086

C 2.00888949 -4.52017532 -2.57652519

C 0.60552296 -4.25664214 -2.59103953

C 2.70483211 -4.47097639 -1.32059396

C 1.97613456 -4.62332896 -0.09181912

C 3.94761533 -3.91547294 1.20608525

C 2.67229296 -4.55825716 1.16151967

C 4.43763367 -3.42468372 2.46510787

C 3.91098034 -3.96207182 3.68597414

C 5.06703069 -2.20387623 4.98155839

C 4.40467351 -3.47312221 4.93815466

C 5.18050506 -1.52241179 6.23668787

C 5.03259194 -2.26320628 7.45696510

C 5.15704062 -1.58625594 8.71156276

C 0.73978684 -3.93615428 -10.00700619

C 0.07225022 -3.64180546 -8.76619680

C 2.09902719 -4.26254126 -7.51738358

C 0.68695149 -4.02986744 -7.53430912

C 2.78626756 -4.20971979 -6.26572876

C 2.04804845 -4.38892803 -5.04680004

C 4.00643111 -3.68642531 -3.74278284

C 2.74261334 -4.35255995 -3.79409390

C 4.48277999 -3.22834216 -2.47843891

C 3.97030241 -3.82272779 -1.27588344

C 5.04195567 -2.06544347 0.04406320

C 4.46102262 -3.36336233 -0.01046270

C 5.14840117 -1.40408396 1.29530059

C 5.07500429 -2.15083536 2.51395050

C 5.20063590 -1.46187094 3.76779163

C 2.14988856 -4.15498839 -9.98751187

C 4.09718047 -3.41083539 -8.66875052

C 2.83818819 -4.07798013 -8.72790125

C 4.56663090 -2.94954953 -7.40086530

C 4.04272130 -3.53942306 -6.20958315

C 5.09633310 -1.78210564 -4.86233666

C 4.51298125 -3.07773270 -4.93738003

C 5.14043131 -1.12775616 -3.59246046

C 5.04838535 -1.91863953 -2.40960090

C 5.08179447 -1.26352740 -1.14157702

C 5.26950545 -1.55578539 -9.79077803

C 4.64640255 -2.83785582 -9.86699437

C 5.30985789 -0.88473944 -8.51882935

C 5.17481568 -1.65636738 -7.32337319

C 5.21988914 -0.99875333 -6.05608092

C 0.08852219 -3.58689080 -11.23981881

C -0.93908756 -2.56926739 -11.21741809

C 2.91709440 -4.01773010 -11.19532327

C 4.19646078 -3.34562523 -11.13362862

C 5.45691316 -0.77821439 -10.98427631

C 5.21642597 0.64685299 -10.92764314

C 3.18387811 2.66103817 -10.90381535

C 1.75453011 2.87859852 -10.93816406

C -0.77875515 1.54902538 -11.05276249

C -1.41601535 0.25204259 -11.11473070

C 0.79535990 -3.81609897 -12.44773632

C 2.21504265 -4.03229691 -12.42629328

C 4.70116700 -2.72961472 -12.30561960

C 5.33712653 -1.44414505 -12.23066318

C 4.86829793 1.32336204 -12.12421530

C 3.84756723 2.33307654 -12.11286854

C -0.20138626 2.08615474 -12.23093803

C 1.07084884 2.74994246 -12.17314231

C -1.20562011 -1.84597819 -12.40790833

C -1.44321968 -0.43092734 -12.35611143

C 2.77162992 -3.46317212 -13.63324255

C 4.01268208 -2.81538949 -13.57417947

C -0.52795430 -2.13673652 -13.65183857

C 0.47082138 -3.11838697 -13.67198916

C -0.91467718 0.15613770 -13.56786661

C -0.29313612 1.41003005 -13.50503447

C 1.76829555 2.48624895 -13.41153382

C 3.15334673 2.27844168 -13.38047395

C 5.04493551 -0.73296689 -13.45468294

C 4.81199499 0.64708141 -13.40153173

C 1.69335099 -2.89538738 -14.41576674

C -0.34186720 -0.89766858 -14.37864973

C 0.92659826 1.65501289 -14.24688977

C 3.74713665 1.23327664 -14.18838329

C 4.22163167 -1.57758880 -14.29569287

C 1.49698645 0.62869293 -15.02578417

C 2.93254169 0.41508279 -14.99705059

C 3.17352313 -1.01523332 -15.05146045

C 1.88718291 -1.68548780 -15.11208354

C 0.85085383 -0.66941264 -15.09444132

C 1.75493789 -4.90255199 12.28210997

C 0.33446751 -4.63026792 12.25623658

C 4.33695179 -3.67136537 12.35920351

C 5.02756547 -2.40079862 12.39976997

C 4.67123012 0.44010814 12.46050145

C 3.68624334 1.49970926 12.46837634

C 0.87605204 2.04056833 12.41434520

C -0.42873051 1.41826317 12.36334294

C -1.79325138 -1.09638690 12.27073044

C -1.61395363 -2.53174305 12.24801792

C 2.44784951 -4.77890412 13.51184217

C 3.74506456 -4.16215300 13.54996040

C 5.09097082 -1.69836115 13.62984207

C 4.91110270 -0.27335670 13.66046624

C 2.99297460 1.78015665 13.67175922

C 1.58187519 2.04915341 13.64393977

C -1.64122793 -0.41321746 13.50305301

C -0.95420934 0.84745334 13.54962294

C -0.31008453 -4.25065739 13.45952199

C -1.29091630 -3.20054189 13.45515048

C 3.87061298 -3.46895640 14.81256104

C 4.54299180 -2.24074423 14.85270047

C 0.39136429 -4.20037612 14.72228624

C 1.76760255 -4.46299711 14.74772354

C -1.20005340 -2.50186755 14.71695135

C -1.37578711 -1.11217244 14.74078535

C -0.25929257 0.92807022 14.81519931

C 1.00652531 1.52634456 14.86259013

C 4.25053085 0.06781148 14.90081157

C 3.29231573 1.09017507 14.90597709

C 2.64578558 -3.65037392 15.56377461

C -0.15634835 -3.11648784 15.51126367

C -0.51532323 -0.28633614 15.56186504

C 2.06279064 0.92995427 15.65435138

C 4.01662529 -1.15028565 15.64782826

C 0.51091195 -0.87689086 16.32643405

C 1.82349903 -0.25876262 16.37200893

C 2.81798038 -1.31637330 16.37031170

C 2.12009272 -2.58837535 16.32668699

C 0.69426602 -2.31662082 16.29995387

H -0.15947920 4.67606499 0.23711649

C -0.52668452 5.70749125 0.20014940

H -0.25023553 6.15587047 -0.75315933

H -1.61091238 5.71146040 0.30340021

H -0.08140158 6.27921802 1.01323627

Table S20. The coordinate of the structures for “d” defective nanotube adsorbed with NH_3_ for physical adsorption.

C 4.03716082 -2.36334550 10.36588549

C 4.03643711 -2.38865494 5.41792150

C 3.99956777 -1.65747790 6.65648733

C 4.02886111 -2.37271534 7.89173896

C 3.47454581 -0.31247920 9.11330402

C 3.98398813 -1.64465202 9.12206852

C 3.01443613 0.27314527 10.34224544

C 3.77871714 -2.34537990 0.48338726

C 3.87944441 -1.66545927 1.71589228

C 4.02255392 -2.38782706 2.95006324

C 3.57770386 -0.30505160 4.19765648

C 4.03271254 -1.66836390 4.18496236

C 3.08486214 0.26238152 5.41415243

C 3.50825669 -0.31866796 6.65394347

C 1.83458124 1.02232426 7.84518095

C 3.03950885 0.24963734 7.87517926

C 1.11571397 1.19603642 9.06893033

C 1.81161946 1.04035406 10.31947524

C 3.79761113 -2.20133717 -4.38846855

C 3.63582256 -1.50609801 -3.15131567

C 3.66581803 -2.24133442 -1.93074652

C 2.83214238 -0.29092278 -0.60326738

C 3.51460511 -1.55703429 -0.68019257

C 2.44383559 0.14463856 0.72919015

C 3.33250530 -0.36215792 1.73504043

C 1.91763637 1.01791247 2.89399240

C 3.11394456 0.26063961 2.99267210

C 1.20101243 1.20879448 4.10946160

C 1.88706832 1.03176685 5.36750159

C -0.26458623 1.05642878 6.57387614

C 1.15506388 1.18993658 6.59594638

C -0.94974226 0.79286421 7.80373449

C -0.30607931 1.08541253 9.04837108

C -2.09311402 -0.08544771 10.28159926

C -1.00347436 0.83644365 10.28324555

C 3.98033460 -2.17008734 -9.33521057

C 3.87924755 -1.45795501 -8.09682979

C 3.90123476 -2.18103013 -6.86312443

C 3.25277000 -0.14970658 -5.61623091

C 3.78155781 -1.47035443 -5.61970553

C 2.75998426 0.40907297 -4.38578997

C 3.10720828 -0.18147511 -3.12210818

C 1.52308909 1.28814812 -1.98004965

C 2.58070467 0.35372522 -1.88961455

C 0.84300758 1.70714744 -0.79413903

C -0.07236853 0.75190377 1.58405214

C 1.35978612 0.87141059 1.54737403

C -0.78416064 0.60665451 2.84134429

C -0.20991620 0.99060843 4.08973648

C -1.96994883 -0.20131409 5.33669805

C -0.89700902 0.73226914 5.32283298

C -2.38994459 -0.78048143 6.58054906

C -2.03524430 -0.14167681 7.80766666

C -2.80281969 -2.11270465 9.06425321

C -2.44184846 -0.73194538 9.04539885

C -2.80032211 -2.82564330 10.31273348

C 3.38740024 -0.13020313 -10.58165667

C 3.95745932 -1.43874433 -10.57454641

C 2.86924557 0.40146791 -9.34882640

C 3.32268010 -0.13944099 -8.10172210

C 1.60323821 1.13829251 -6.91327167

C 2.82563780 0.40538995 -6.87473465

C 0.87207116 1.30782336 -5.69405382

C 1.56697208 1.19081741 -4.44583077

C -0.57208864 1.20430272 -3.25830966

C 0.84276259 1.42151198 -3.24320070

C -1.15994785 0.80288691 -2.01781715

C -0.45719177 1.12597307 -0.81031508

C -2.00200251 -0.30121955 0.43645983

C -0.91225494 0.60705803 0.41977420

C -2.34863141 -0.91083043 1.67204217

C -1.91212548 -0.27572474 2.87539689

C -2.76416028 -2.20413090 4.13507263

C -2.34676330 -0.83456939 4.11310040

C -2.73810623 -2.90395953 5.37988637

C -2.76796224 -2.15427956 6.60155541

C -2.25605272 -4.19604629 7.86816559

C -2.75686982 -2.85516330 7.84523755

C -1.80473701 -4.73381942 9.11160072

C -2.28692570 -4.15720138 10.33692916

C 0.94610150 1.28841313 -10.62521535

C 1.63976309 1.12255182 -9.37670251

C -0.50679517 1.11629263 -8.17737699

C 0.91431081 1.28085977 -8.15660692

C -1.17719577 0.81173612 -6.95362535

C -0.53923430 1.14129700 -5.71347574

C -2.20815898 -0.18212524 -4.48246821

C -1.20723495 0.84242527 -4.48165121

C -2.52971707 -0.83452216 -3.25318275

C -2.14433179 -0.22200063 -2.01570637

C -2.82399419 -2.25444072 -0.78496478

C -2.46439661 -0.87266775 -0.78540907

C -2.77723540 -2.98181190 0.44440473

C -2.78450529 -2.25739906 1.68087906

C -2.26848365 -4.29977828 2.93333077

C -2.75892936 -2.96075581 2.92400215

C -1.80745766 -4.83737577 4.17381848

C -2.25260756 -4.24198010 5.39882702

C -0.59167194 -5.54106790 6.66007887

C -1.79633930 -4.78031790 6.64712306

C 0.10510753 -5.69442209 7.89937814

C -0.60045336 -5.49919087 9.12766729

C 1.51962673 -5.56751220 10.37830460

C 0.09628675 -5.67208907 10.37338025

C -0.47212622 1.13003795 -10.64588005

C -2.19290787 -0.16116542 -9.42781473

C -1.14476066 0.80543771 -9.41725072

C -2.55520802 -0.79616651 -8.19713623

C -2.20833471 -0.17264912 -6.95904715

C -2.83871099 -2.20989461 -5.72817486

C -2.54949498 -0.81577218 -5.72052664

C -2.78023943 -2.94058799 -4.49645500

C -2.83333779 -2.23090475 -3.25794196

C -2.25612507 -4.29199036 -2.02514846

C -2.77697440 -2.96598437 -2.02631611

C -1.80393376 -4.86337621 -0.78603623

C -2.26962802 -4.31481090 0.45099094

C -0.59488585 -5.61031280 1.70505081

C -1.80757406 -4.86471031 1.69545350

C 0.11055217 -5.76414058 2.94931596

C -0.59159707 -5.58986846 4.18350742

C 1.53168034 -5.59261110 5.42602071

C 0.11410332 -5.72749895 5.42303000

C 2.19536745 -5.29895872 6.66609115

C 1.53192840 -5.57557326 7.90302115

C 3.28012819 -4.36089243 9.13415831

C 2.19247901 -5.28573270 9.13779344

C 3.68113723 -3.74543075 10.37244145

C -2.85208944 -2.17621462 -10.68338149

C -2.55751979 -0.77950765 -10.67395596

C -2.76831700 -2.90690831 -9.44566464

C -2.84675341 -2.19774019 -8.20551847

C -2.22508891 -4.23685604 -6.97869767

C -2.77514051 -2.92174705 -6.97351084

C -1.76609966 -4.80111165 -5.74000597

C -2.24562353 -4.26707796 -4.50019554

C -0.58085846 -5.59443488 -3.26257957

C -1.79106421 -4.83663271 -3.26799473

C 0.11350962 -5.77375034 -2.01788139

C -0.59305788 -5.62104946 -0.77667179

C 1.52904655 -5.65189890 0.47555664

C 0.10662027 -5.78628852 0.46521049

C 2.19182147 -5.34500157 1.71346145

C 1.53218922 -5.62524580 2.95575546

C 3.27829572 -4.38308011 4.18605255

C 2.19703278 -5.32261392 4.18735122

C 3.65984018 -3.76320856 5.42019931

C 3.27641318 -4.37056029 6.66312597

C 3.66538309 -3.75853652 7.89682580

C -1.72014897 -4.76591809 -10.68949898

C -2.20494266 -4.21755833 -9.45022938

C -0.51693162 -5.50670208 -8.20832717

C -1.74638419 -4.77399653 -8.21492274

C 0.17126849 -5.67812280 -6.96785505

C -0.55308366 -5.54547977 -5.73513190

C 1.55629804 -5.57623805 -4.47752038

C 0.13576870 -5.73532051 -4.49326943

C 2.19579914 -5.29404771 -3.23376554

C 1.52881478 -5.63135285 -2.00780901

C 3.19727727 -4.35683016 -0.75391056

C 2.18139260 -5.35331623 -0.76280788

C 3.56604140 -3.75177696 0.47597419

C 3.25124120 -4.39159303 1.71720128

C 3.64784276 -3.76917194 2.94935021

C -0.48769141 -5.48551935 -10.68071686

C 1.62707632 -5.47383740 -9.40988475

C 0.21033205 -5.63509242 -9.43295823

C 2.26219599 -5.18543772 -8.16309266

C 1.58668075 -5.51288216 -6.94761929

C 3.24163331 -4.23182490 -5.66809312

C 2.22259894 -5.22447327 -5.69640586

C 3.55157611 -3.60877304 -4.41934088

C 3.20284624 -4.28139870 -3.21133944

C 3.50205486 -3.65393791 -1.96392284

C 3.37016258 -4.20542149 -10.60350144

C 2.31962458 -5.17151101 -10.63225373

C 3.68335755 -3.56512318 -9.35370866

C 3.30323685 -4.20382600 -8.13302698

C 3.61580548 -3.57809441 -6.88730014

C -2.22668246 -4.23206862 -11.92419219

C -2.80927075 -2.90818711 -11.92040474

C 0.24829397 -5.66832410 -11.90166943

C 1.68583938 -5.51017780 -11.87661884

C 3.80153018 -3.58014033 -11.82285754

C 4.10126962 -2.16528273 -11.80769959

C 2.94845616 0.45370436 -11.82113933

C 1.69754563 1.17915961 -11.84424207

C -1.14819072 0.86729374 -11.88533132

C -2.21705474 -0.10707580 -11.89904026

C -1.68073092 -4.73341279 -13.13313714

C -0.43857615 -5.45432100 -13.12267017

C 2.35375113 -5.15062971 -13.07333489

C 3.41747186 -4.18610570 -13.04625914

C 3.99709599 -1.43778117 -13.02029775

C 3.41755694 -0.12420518 -13.02772865

C -0.44204193 1.12688064 -13.08703581

C 0.98583784 1.27974452 -13.06605040

C -2.81993746 -2.16656238 -13.12922430

C -2.52144461 -0.76218090 -13.11822724

C 0.25974350 -5.15821634 -14.35346638

C 1.65275099 -5.00911306 -14.33001164

C -2.32473917 -2.71546365 -14.37200685

C -1.75542355 -3.99511406 -14.37419384

C -1.84309249 -0.43916224 -14.35429638

C -0.80444650 0.50078787 -14.33827715

C 1.50938321 0.74873940 -14.30450881

C 2.72247622 0.04839039 -14.28427100

C 3.37792692 -3.44757555 -14.28811400

C 3.66769741 -2.07737601 -14.27510953

C -0.55352752 -4.25380963 -15.13963313

C -1.71475760 -1.64888359 -15.13917371

C 0.40274026 0.26373911 -15.10291667

C 2.87364429 -1.16104894 -15.06658442

C 2.28358662 -3.95200135 -15.09224816

C 0.53976372 -0.91889833 -15.85660346

C 1.79774735 -1.64313340 -15.83926953

C 1.49706839 -3.06310394 -15.85199130

C 0.05364928 -3.21644208 -15.87550637

C -0.53808370 -1.89110728 -15.87658538

C -0.64062845 -5.51480629 11.59834115

C -1.86202516 -4.73990432 11.57977366

C 2.21355333 -5.31546658 11.61236402

C 3.32169130 -4.38548254 11.60858709

C 4.03269294 -1.61149782 11.59177692

C 3.50590934 -0.26415647 11.58027401

C 1.09069465 1.27039236 11.54132028

C -0.35208321 1.16911610 11.52205169

C -2.54408182 -0.67168263 11.51523962

C -2.90411347 -2.07277051 11.53178572

C 0.07385105 -5.62609085 12.81672106

C 1.50735453 -5.52757901 12.82307320

C 3.66305976 -3.72770898 12.81733686

C 4.01853135 -2.33594089 12.80894322

C 2.98867577 0.27804543 12.78281298

C 1.77475086 1.04618903 12.76277999

C -2.12799682 -0.06427228 12.72597120

C -1.02619038 0.85729913 12.72953517

C -2.29834148 -4.12277769 12.77815496

C -2.82533637 -2.78611361 12.75395481

C 1.90375073 -4.90005871 14.06384241

C 2.97993591 -4.00348245 14.06119581

C -1.60228073 -4.30510594 14.03167420

C -0.41805764 -5.05408147 14.04999666

C -2.45937679 -2.14117691 13.99432014

C -2.11273365 -0.78383461 13.98017979

C -0.32496407 0.70560452 13.98506734

C 1.07267316 0.79844334 14.00194831

C 3.55507956 -1.74843529 14.04630970

C 3.03909685 -0.44582583 14.03279904

C 0.71328891 -4.60206432 14.83302155

C -1.69828365 -3.07805327 14.79494491

C -0.99322365 -0.31304468 14.76854143

C 1.85226291 -0.12647013 14.79879309

C 2.90642969 -2.77834322 14.83044078

C -0.23989646 -1.22173792 15.53884776

C 1.20855862 -1.12781001 15.55277642

C 1.74561496 -2.47645648 15.57047945

C 0.62920985 -3.40414753 15.57069322

C -0.59783513 -2.62851275 15.55144932

H -0.63482965 3.87862651 -2.64075518

N -0.01215362 4.37747114 -2.00141253

H 0.24347244 3.75891024 -1.22406523

H 0.83276303 4.66097430 -2.50159442

Table S21. The coordinate of the structures for “d” defective nanotube adsorbed with H_2_O for physical adsorption.

C 3.43349874 -0.39396365 10.51053344

C 3.42378302 -0.42994097 5.56347908

C 3.37918110 0.30313546 6.80058446

C 3.42066813 -0.40919570 8.03672503

C 2.84020555 1.64579760 9.25480637

C 3.36813771 0.32101754 9.26534463

C 2.37523784 2.22883033 10.48279499

C 3.15095471 -0.39592286 0.63085520

C 3.24780164 0.28622581 1.86177517

C 3.40466181 -0.43276917 3.09622622

C 2.93682139 1.64602874 4.34169053

C 3.40886696 0.28839001 4.32986139

C 2.43672904 2.20883625 5.55729027

C 2.86932926 1.63494335 6.79660494

C 1.18110930 2.95783388 7.98740710

C 2.39517199 2.19964317 8.01689181

C 0.46239917 3.12582337 9.21154227

C 1.16300764 2.98100080 10.46051106

C 3.16988841 -0.26102192 -4.23799254

C 2.99292328 0.43428299 -3.00304259

C 3.03256918 -0.29776811 -1.78130566

C 2.17439388 1.64593794 -0.45702468

C 2.87115132 0.38741779 -0.53274141

C 1.79107094 2.08148102 0.87638019

C 2.68703429 1.58332584 1.88007903

C 1.25844961 2.94562395 3.04168357

C 2.46510979 2.20466223 3.13770151

C 0.54008875 3.13253369 4.25532992

C 1.22998587 2.96396926 5.51168868

C -0.92077376 2.96796098 6.71863842

C 0.49746643 3.11621170 6.73976075

C -1.60095566 2.69858388 7.94977682

C -0.95803536 2.99996761 9.19287798

C -2.72880223 1.81133308 10.43117144

C -1.64997143 2.74580762 10.42955877

C 3.36363250 -0.23835041 -9.18411884

C 3.25034323 0.47508521 -7.94751362

C 3.27960061 -0.24477220 -6.71215304

C 2.60132626 1.78077045 -5.47083466

C 3.14646037 0.46687805 -5.47061819

C 2.09814623 2.33576782 -4.24274496

C 2.44532332 1.75084895 -2.97698574

C 0.85209970 3.21572501 -1.84393940

C 1.90897056 2.28299583 -1.74612819

C 0.16966274 3.65064868 -0.65548604

C -0.72786466 2.66402515 1.72847895

C 0.70160847 2.79208433 1.69919086

C -1.43918209 2.51149601 2.98561427

C -0.86856890 2.90132454 4.23432545

C -2.61240179 1.68892740 5.48406745

C -1.55145827 2.63580479 5.46853675

C -3.02345314 1.10645811 6.72989820

C -2.67557709 1.75160185 7.95618058

C -3.41432312 -0.22689126 9.21802807

C -3.07210279 1.15853922 9.19621153

C -3.39990635 -0.93705853 10.46842645

C 2.74489352 1.79037530 -10.43628547

C 3.33253546 0.48962945 -10.42526952

C 2.21801438 2.31807055 -9.20539456

C 2.67656723 1.78623427 -7.95633929

C 0.93951605 3.04585411 -6.77416318

C 2.17053761 2.32785592 -6.73155243

C 0.20441788 3.21175326 -5.55697114

C 0.89895379 3.10730207 -4.30800651

C -1.24102312 3.10161540 -3.12075069

C 0.17166914 3.33796896 -3.10751968

C -1.81922087 2.69229906 -1.87660368

C -1.12353526 3.03205816 -0.67119068

C -2.63613794 1.57506205 0.57981598

C -1.56123485 2.50044720 0.55908026

C -2.97627305 0.96548512 1.81813355

C -2.55323760 1.61109347 3.02119659

C -3.37730130 -0.32716978 4.28577626

C -2.97986421 1.04839208 4.26051947

C -3.34214744 -1.02377723 5.53286906

C -3.38201674 -0.27262857 6.75387471

C -2.84110105 -2.30452912 8.02483797

C -3.35996344 -0.97052861 7.99959250

C -2.38125077 -2.83405383 9.26910378

C -2.86869020 -2.26159652 10.49444747

C 0.28511893 3.17696421 -10.48618808

C 0.97944238 3.02340622 -9.23678956

C -1.16863431 2.99383213 -8.03933474

C 0.25036090 3.17607442 -8.01829930

C -1.83641058 2.68430636 -6.81519455

C -1.20455442 3.02755219 -5.57575385

C -2.85256973 1.68206446 -4.34024441

C -1.86870383 2.72372892 -4.34241980

C -3.16101285 1.02719485 -3.10875810

C -2.78380152 1.64803801 -1.87257368

C -3.43562978 -0.39084627 -0.63650139

C -3.09215361 0.99537739 -0.64073912

C -3.37950584 -1.11377552 0.59515834

C -3.39495157 -0.38628188 1.83046509

C -2.85665003 -2.41958649 3.08823760

C -3.36223647 -1.08608279 3.07588279

C -2.38901049 -2.94865318 4.33017641

C -2.83974198 -2.35549195 5.55455661

C -1.16254706 -3.63223794 6.81765140

C -2.37599323 -2.88549374 6.80407167

C -0.46232119 -3.77490993 8.05641517

C -1.16789932 -3.58487185 9.28543026

C 0.95497024 -3.62701168 10.53346401

C -0.46710526 -3.74758540 10.53052670

C -1.13091346 3.00025850 -10.50747922

C -2.83626544 1.69020390 -9.28691030

C -1.80092216 2.67063909 -9.27864579

C -3.19042991 1.05363391 -8.05445184

C -2.85320846 1.68517552 -6.81771233

C -3.45352134 -0.35787095 -5.58096480

C -3.18420783 1.04047136 -5.57669007

C -3.38495823 -1.08466501 -4.34736613

C -3.44572886 -0.37308921 -3.11001942

C -2.84589902 -2.42504893 -1.87210877

C -3.38061100 -1.10449551 -1.87633650

C -2.38781497 -2.98842712 -0.63138011

C -2.85835643 -2.44133124 0.60494855

C -1.17004877 -3.71582608 1.86201048

C -2.39036056 -2.98286091 1.85125535

C -0.46215324 -3.85838101 3.10637801

C -1.16503151 -3.68793206 4.34083201

C 0.95945184 -3.66371622 5.58129835

C -0.45648882 -3.81432907 5.58008154

C 1.62155376 -3.35977095 6.81976423

C 0.96308611 -3.64075614 8.05820070

C 2.69957820 -2.40374124 9.28434768

C 1.62251418 -3.34079727 9.29127477

C 3.09529863 -1.78041911 10.52060171

C -3.46658858 -0.33689901 -10.53775391

C -3.19092919 1.06367291 -10.53183450

C -3.37369152 -1.06293223 -9.29769714

C -3.46228629 -0.35203067 -8.05922157

C -2.81387534 -2.37951277 -6.82699615

C -3.38074052 -1.07160001 -6.82499112

C -2.34895897 -2.93571698 -5.58682184

C -2.83523331 -2.40515943 -4.34806521

C -1.15651687 -3.71158781 -3.10720379

C -2.37494663 -2.96708722 -3.11423330

C -0.46074271 -3.88079290 -1.86171733

C -1.16903583 -3.73348459 -0.62051116

C 0.95322315 -3.73609563 0.63139522

C -0.46742231 -3.88756387 0.62190635

C 1.61287343 -3.41747166 1.86786534

C 0.95784362 -3.70316987 3.11131872

C 2.68963541 -2.43625115 4.33676594

C 1.62027448 -3.38933028 4.34128770

C 3.06531227 -1.80928285 5.56888908

C 2.69177897 -2.41901091 6.81356051

C 3.07559028 -1.79968048 8.04518191

C -2.29997902 -2.91103551 -10.53653613

C -2.79285802 -2.36604438 -9.29885539

C -1.08946524 -3.63072053 -8.05326935

C -2.32787624 -2.91324951 -8.06198277

C -0.40029902 -3.79169184 -6.81209518

C -1.12740318 -3.66610625 -5.58011935

C 0.98104766 -3.66944340 -4.32187333

C -0.43750708 -3.84609239 -4.33751646

C 1.61564394 -3.37638664 -3.07831326

C 0.95265469 -3.72094508 -1.85213249

C 2.60112646 -2.41928009 -0.60124517

C 1.60120436 -3.43155107 -0.60779755

C 2.96131864 -1.80569328 0.62636129

C 2.65866631 -2.44896357 1.86862076

C 3.04871583 -1.81906604 3.09870492

C -1.05830718 -3.61446221 -10.52541514

C 1.05503327 -3.57339802 -9.25343444

C -0.35958530 -3.75236062 -9.27691792

C 1.68518351 -3.27436644 -8.00691977

C 1.01285950 -3.60878071 -6.79157497

C 2.64846760 -2.30293633 -5.51377340

C 1.64376538 -3.31020246 -5.54052822

C 2.94653768 -1.67229508 -4.26618098

C 2.60671924 -2.34816022 -3.05772565

C 2.89373365 -1.71277323 -1.81186549

C 2.78246701 -2.28475589 -10.44836873

C 1.74473175 -3.26459030 -10.47571267

C 3.08554307 -1.63740221 -9.19978198

C 2.71289611 -2.27877102 -7.97825699

C 3.01466655 -1.64590159 -6.73353345

C -2.81257059 -2.38737836 -11.77307618

C -3.41278216 -1.07134478 -11.77301664

C -0.31893654 -3.79094848 -11.74533978

C 1.11642809 -3.61427569 -11.71970924

C 3.20660682 -1.65659792 -11.66882726

C 3.48750397 -0.23781818 -11.65666346

C 2.29988481 2.36560283 -11.67769416

C 1.03953395 3.07451415 -11.70394316

C -1.80198283 2.72545177 -11.74687180

C -2.85800272 1.73719269 -11.75861795

C -2.25894586 -2.88444552 -12.98027087

C -1.00753686 -3.58923192 -12.96730995

C 1.78053416 -3.24882899 -12.91666839

C 2.83175386 -2.27048777 -12.89108667

C 3.37459812 0.48529962 -12.87110213

C 2.77799825 1.79126461 -12.88241757

C -1.09793792 2.99131915 -12.94841391

C 0.32783679 3.16272818 -12.92651812

C -3.43217220 -0.33322052 -12.98389404

C -3.15207135 1.07493752 -12.97632964

C -0.31208876 -3.28700228 -14.19822949

C 1.07886061 -3.11966427 -14.17420222

C -2.92842190 -0.87857586 -14.22484898

C -2.34236516 -2.15059203 -14.22343727

C -2.47683840 1.40378361 -14.21264031

C -1.45071288 2.35741803 -14.19833010

C 0.85967978 2.63588024 -14.16319330

C 2.08190120 1.95163387 -14.13998288

C 2.78336438 -1.53549218 -14.13468095

C 3.05501622 -0.16154451 -14.12476615

C -1.13651884 -2.39526822 -14.98728278

C -2.33174184 0.19392384 -14.99428367

C -0.23976154 2.13437791 -14.96138600

C 2.24972240 0.74230067 -14.91920447

C 1.69642736 -2.05620940 -14.93842857

C -0.08648279 0.95178058 -15.71194804

C 1.18096920 0.24421219 -15.69166430

C 0.89894622 -1.17962992 -15.70094600

C -0.54232670 -1.35195547 -15.72529445

C -1.15138831 -0.03456503 -15.73024613

C -1.20358184 -3.59665527 11.75633873

C -2.43428879 -2.83660402 11.73782705

C 1.64822880 -3.36434948 11.76587270

C 2.74555665 -2.42154580 11.75874203

C 3.42145297 0.36107469 11.73434865

C 2.87695579 1.70137925 11.72066536

C 0.44216019 3.20608372 11.68280793

C -0.99935734 3.08830381 11.66623670

C -3.16907077 1.22193320 11.66717596

C -3.51102077 -0.18360228 11.68671533

C -0.48574251 -3.69706082 12.97386770

C 0.94643641 -3.58172258 12.97804607

C 3.08059686 -1.75644884 12.96545048

C 3.41868250 -0.36037753 12.95325670

C 2.35542939 2.24053082 12.92264558

C 1.13189690 2.99329339 12.90304859

C -2.75730894 1.83641659 12.87607555

C -1.66686493 2.77131306 12.87585104

C -2.87590503 -2.22284495 12.93597643

C -3.42003659 -0.89329193 12.91030856

C 1.33746872 -2.94694243 14.21697115

C 2.40270757 -2.03740363 14.21096734

C -2.17555455 -2.39427781 14.18878564

C -0.98227400 -3.12858556 14.20686274

C -3.05989972 -0.24163024 14.14900667

C -2.73034071 1.11979685 14.13182604

C -0.96097979 2.63087796 14.13023629

C 0.43553592 2.74051890 14.14430450

C 2.94990393 0.22462826 14.18989168

C 2.41759997 1.52064769 14.17435851

C 0.14476964 -2.66155007 14.98730665

C -2.28543643 -1.16721616 14.95000491

C -1.61511769 1.60586015 14.91713035

C 1.22832024 1.82737352 14.94179435

C 2.31542663 -0.81130806 14.97754159

C -0.84889359 0.70828286 15.68780696

C 0.59834564 0.81997614 15.69909339

C 1.15214504 -0.52190808 15.71871170

C 0.04722781 -1.46316920 15.72256638

C -1.18930533 -0.70275411 15.70371298

O -0.34342916 6.03010747 -1.30580978

H -0.08801385 5.16938774 -0.86412931

H -0.43059422 5.82995189 -2.25248381

Table S22. The coordinate of the structures for perfect nanotube adsorbed with CH_4_ for physical adsorption.

C 3.12357656 -0.67311504 9.87257838

C 3.09549352 -0.67249931 4.92611885

C 3.02048928 0.04811987 6.16501049

C 3.10277705 -0.66837582 7.39967841

C 2.44524305 1.35334633 8.63321962

C 3.02696269 0.04986461 8.63323817

C 1.97151866 1.90813677 9.87249529

C 3.09264183 -0.67399886 -0.01675030

C 3.01429195 0.04624575 1.21950054

C 3.09661686 -0.67272191 2.45774866

C 2.44180477 1.35322335 3.69049870

C 3.02455824 0.04692667 3.69043038

C 1.95365861 1.88660330 4.92612754

C 2.43974948 1.34950029 6.16502805

C 0.72103903 2.60421556 7.40060417

C 1.96136998 1.88936132 7.39963886

C 0.01589816 2.75325171 8.63504414

C 0.73475784 2.62085448 9.87345463

C 3.09297150 -0.67497279 -4.95685585

C 3.01382084 0.04373499 -3.72414513

C 3.08500732 -0.67674572 -2.48621946

C 2.43396974 1.34952853 -1.24969146

C 3.01252092 0.04338807 -1.24965591

C 1.95288688 1.88773612 -0.01665773

C 2.43507431 1.34679892 1.21956481

C 0.71794876 2.60112632 2.45888242

C 1.95466162 1.88787479 2.45789516

C 0.01252422 2.75376145 3.69240822

C 0.72003854 2.59778933 4.92711904

C -1.40431446 2.59802977 6.16830967

C 0.01282043 2.74831250 6.16686509

C -2.06414958 2.30880278 7.40343181

C -1.40351978 2.60274419 8.63649485

C -3.13588060 1.36650295 9.87782894

C -2.07609138 2.32275720 9.87635428

C 3.08710159 -0.67768247 -9.89991341

C 3.01312938 0.04060922 -8.66622256

C 3.08516053 -0.67707166 -7.43154280

C 2.42940080 1.34374270 -6.19264744

C 3.00963845 0.04334183 -6.19250227

C 1.95084105 1.88621316 -4.95720545

C 2.43321935 1.34794672 -3.72427335

C 0.71346779 2.59613923 -2.48566219

C 1.94618147 1.88405190 -2.48644494

C 0.00751201 2.75181999 -1.24819918

C 0.71550604 2.60252742 -0.01586698

C -1.40819133 2.59706319 1.22286413

C 0.00748608 2.74748712 1.22156249

C -2.06975699 2.30541870 2.46156811

C -1.40982923 2.60295382 3.69382719

C -3.12368505 1.34830317 4.93128980

C -2.06658256 2.30230312 4.92990563

C -3.48403493 0.72141411 6.17103949

C -3.12703091 1.34976479 7.40485222

C -3.78077975 -0.67199661 8.64026064

C -3.48523452 0.72446940 8.63934435

C -3.72100380 -1.39886809 9.87969228

C 2.44562017 1.35278754 -11.13934843

C 3.02738677 0.04929175 -11.13929903

C 1.94441467 1.88269996 -9.89997722

C 2.42966166 1.34793083 -8.66630636

C 0.70891135 2.59255080 -7.43110455

C 1.94363708 1.88097097 -7.43171668

C 0.00157151 2.74322220 -6.19148073

C 0.71172375 2.60078667 -4.95660205

C -1.41467729 2.59811744 -3.72188213

C 0.00494511 2.74904632 -3.72324872

C -2.07313752 2.29990920 -2.48306403

C -1.41350767 2.60071307 -1.24686165

C -3.13266270 1.34833364 -0.01198792

C -2.07219500 2.30623554 -0.01335422

C -3.48817830 0.71882719 1.22537513

C -3.12939244 1.34876599 2.46291266

C -3.78726586 -0.67518040 3.69728032

C -3.49135779 0.72422946 3.69642171

C -3.70355594 -1.39333355 4.93296017

C -3.77901629 -0.67280788 6.17191546

C -3.12339036 -2.69771747 7.40674262

C -3.70681590 -1.39037993 7.40662924

C -2.63803552 -3.23235581 8.64042306

C -3.13916590 -2.70234741 9.87977546

C -0.00352518 2.76387036 -11.13781314

C 0.70762493 2.59530391 -9.89929185

C -1.42078915 2.59523449 -8.66363551

C 0.00281491 2.74622729 -8.66492553

C -2.07665584 2.29706552 -7.42856718

C -1.41440427 2.59296036 -6.19019239

C -3.13836993 1.34640639 -4.95264355

C -2.07661811 2.30479949 -4.95408364

C -3.49533136 0.71932330 -3.71902159

C -3.12957912 1.34558595 -2.48164864

C -3.78936542 -0.67675941 -1.24329432

C -3.49402635 0.72141710 -1.24419310

C -3.71215188 -1.39489283 -0.01031974

C -3.78242415 -0.67408159 1.22623819

C -3.12755184 -2.69786932 2.46461322

C -3.70910897 -1.39406972 2.46452269

C -2.64479194 -3.23593147 3.69750198

C -3.12330764 -2.69368794 4.93308920

C -1.40281635 -3.94254367 6.17138686

C -2.63750418 -3.23090990 6.17214039

C -0.69663654 -4.09619542 7.40519505

C -1.40132954 -3.94509081 8.63964624

C 0.72937024 -3.96308541 9.87667719

C -0.69010130 -4.11364765 9.87811938

C -1.42297707 2.61325617 -11.13650230

C -3.14042377 1.34337576 -9.89533223

C -2.08056070 2.29947377 -9.89670550

C -3.50047914 0.71904787 -8.66089598

C -3.13473653 1.34243333 -7.42715058

C -3.78956684 -0.67747372 -6.18645048

C -3.49488609 0.71562270 -6.18735690

C -3.71842380 -1.39697862 -4.95079969

C -3.79055798 -0.67742106 -3.71810377

C -3.12916134 -2.69672632 -2.47985284

C -3.70903931 -1.39651560 -2.47985454

C -2.64736556 -3.23740347 -1.24323076

C -3.13011602 -2.70003602 -0.01026680

C -1.40772946 -3.94528966 1.22566488

C -2.64081332 -3.23373599 1.22642363

C -0.69922050 -4.09855914 2.46327393

C -1.40572841 -3.95049611 3.69676921

C 0.72040635 -3.94308329 4.93024321

C -0.69558301 -4.09320924 4.93165600

C 1.38275254 -3.64721173 6.16857168

C 0.72698896 -3.94524847 7.40377182

C 2.44663613 -2.69324621 8.63521294

C 1.38683235 -3.64941691 8.63678035

C 2.82810351 -2.06962368 9.87357727

C -3.81757632 -0.67650933 -11.13301455

C -3.52185379 0.71994906 -11.13382520

C -3.72120549 -1.39960926 -9.89368697

C -3.79706594 -0.68148761 -8.66002849

C -3.13395584 -2.69942831 -7.42541625

C -3.71473910 -1.39805304 -7.42539119

C -2.64772967 -3.23657476 -6.18654095

C -3.13572050 -2.70325011 -4.95083232

C -1.41196439 -3.95118281 -3.71905057

C -2.64853986 -3.23777548 -3.71814503

C -0.70173040 -4.09741691 -2.48157278

C -1.40970020 -3.95181445 -1.24405901

C 0.71934628 -3.95144050 -0.01303060

C -0.70182582 -4.10139517 -0.01172265

C 1.37909396 -3.65064071 1.22315686

C 0.72044930 -3.94828108 2.46195146

C 2.44426172 -2.69648789 3.69251472

C 1.38253017 -3.65494139 3.69408854

C 2.80074018 -2.06558526 4.92717188

C 2.44072815 -2.69244697 6.16699915

C 2.80646885 -2.06896809 7.40069110

C -2.66540007 -3.25756764 -11.13302867

C -3.13931094 -2.70300787 -9.89370160

C -1.41506465 -3.95383746 -8.66097829

C -2.65548752 -3.23910905 -8.66008586

C -0.70690309 -4.09809685 -7.42721153

C -1.41412908 -3.94774022 -6.18745606

C 0.71570412 -3.95352708 -4.95408409

C -0.70669368 -4.10401653 -4.95269683

C 1.37573957 -3.65658066 -3.72176151

C 0.71406154 -3.94806308 -2.48296385

C 2.43845652 -2.69961600 -1.24798753

C 1.37815807 -3.65760367 -1.24664973

C 2.79946242 -2.07241390 -0.01584226

C 2.43566392 -2.69643926 1.22174550

C 2.80128457 -2.06952484 2.45883307

C -1.42855727 -3.97015935 -11.13387594

C 0.70960546 -3.95221015 -9.89669071

C -0.70981911 -4.10268523 -9.89538156

C 1.37016539 -3.65847791 -8.66355196

C 0.71022275 -3.94782624 -7.42855274

C 2.42949155 -2.69832109 -6.19124022

C 1.37245234 -3.65240509 -6.19006715

C 2.79709723 -2.07445136 -4.95623746

C 2.43528682 -2.69978268 -3.72307700

C 2.79334395 -2.06989478 -2.48540649

C 2.44218535 -2.71603576 -11.13770486

C 1.38227517 -3.67212720 -11.13646811

C 2.79150224 -2.07411910 -9.89915289

C 2.43311446 -2.69952102 -8.66476186

C 2.78999534 -2.07127027 -7.43086126

C -3.19535180 -2.73848656 -12.36461910

C -3.78505761 -1.41759442 -12.36462648

C -0.71488776 -4.16752298 -12.36627362

C 0.72359575 -4.01501029 -12.36759831

C 2.84911852 -2.09761050 -12.37019155

C 3.14856641 -0.68240831 -12.37100381

C 1.98190220 1.93166632 -12.37104769

C 0.72855450 2.65389153 -12.37029117

C -2.11806764 2.35186972 -12.36759103

C -3.19214309 1.38292588 -12.36622478

C -2.66746332 -3.24880360 -13.57680832

C -1.42236371 -3.96616373 -13.57764665

C 1.37087178 -3.67004491 -13.58024615

C 2.43785620 -2.70754759 -13.58154041

C 3.01918504 0.04046935 -13.58312869

C 2.43358720 1.35269154 -13.58317346

C -1.42910764 2.60339640 -13.58025005

C -0.00016631 2.75497489 -13.58161197

C -3.81238574 -0.68390963 -13.57684734

C -3.51478127 0.72190443 -13.57760155

C -0.74581925 -3.68223899 -14.82311552

C 0.64751898 -3.53454095 -14.82440411

C -3.33358315 -1.24098876 -14.82182404

C -2.76241458 -2.52043583 -14.82177977

C -2.85163188 1.03588380 -14.82304579

C -1.81127506 1.97444213 -14.82438906

C 0.50301228 2.21993400 -14.82661423

C 1.71705158 1.52040380 -14.82735264

C 2.37556616 -1.97566435 -14.82652041

C 2.66553883 -0.60485708 -14.82729629

C -1.57180142 -2.78399872 -15.60317856

C -2.73462100 -0.17873118 -15.60316312

C -0.61618373 1.73228071 -15.60584443

C 1.85580135 0.30803285 -15.60738331

C 1.26529236 -2.48320128 -15.60580060

C -0.49119038 0.54656155 -16.35712564

C 0.76663836 -0.17813553 -16.35793539

C 0.46615055 -1.59835015 -16.35709399

C -0.97739978 -1.75141368 -16.35579446

C -1.56907371 -0.42579696 -16.35577772

C -1.42206384 -4.00337992 11.11066871

C -2.67537267 -3.28109472 11.11148575

C 1.42453350 -3.70146614 11.10768790

C 2.49861986 -2.73251602 11.10609455

C 3.09116171 0.06814972 11.10408609

C 2.50155553 1.38909590 11.10403667

C 0.02119328 2.81824488 11.10591123

C -1.41728305 2.66570666 11.10741121

C -3.54269022 0.74814110 11.11038961

C -3.84212092 -0.66707475 11.11134662

C -0.69327212 -4.10428843 12.32196611

C 0.73567564 -3.95275437 12.32046458

C 2.82124717 -2.07125790 12.31736593

C 3.11870948 -0.66542795 12.31634597

C 1.97385156 1.89955024 12.31626767

C 0.72877145 2.61694649 12.31722693

C -3.13129685 1.35816476 12.32165201

C -2.06437083 2.32072240 12.32016627

C -3.12696090 -2.70200746 12.32359584

C -3.71271021 -1.38985140 12.32353498

C 1.11793983 -3.32364770 13.56450441

C 2.15828754 -2.38510990 13.56295018

C -2.41041817 -2.86961580 13.56777661

C -1.19637146 -3.56910757 13.56696420

C -3.35899197 -0.74437746 13.56762987

C -3.06895641 0.62641386 13.56669856

C -1.34092053 2.18528506 13.56427371

C 0.05240157 2.33308558 13.56281033

C 2.64005437 -0.10822213 13.56129960

C 2.06897967 1.17125539 13.56127162

C -0.07709171 -3.08140420 14.34603772

C -2.54917305 -1.65718855 14.34775591

C -1.95862238 1.13401820 14.34584654

C 0.87848752 1.43488082 14.34281865

C 2.04128964 -1.17037757 14.34288510

C -1.15939753 0.24925825 15.09714129

C 0.28415533 0.40234912 15.09557442

C 0.87584149 -0.92324656 15.09563374

C -0.20203543 -1.89561583 15.09722713

C -1.45989546 -1.17095927 15.09813659

H 5.42588681 1.79631086 -1.24951164

C 6.05899304 0.91698640 -1.35776581

H 5.89353605 0.24627166 -0.51592765

H 7.10414037 1.22093547 -1.38123634

H 5.80830852 0.40269859 -2.28455980

Table S23. The coordinate of the structures for perfect nanotube adsorbed with NH_3_ for physical adsorption.

C 3.31773062 -0.09549011 10.50263279

C 3.29309561 -0.09394336 5.55696313

C 3.21260369 0.62602208 6.79573369

C 3.29854425 -0.09038138 8.03002664

C 2.62630559 1.92656606 9.26355219

C 3.21730652 0.62722564 9.26359834

C 2.14714714 2.47737639 10.50244827

C 3.29725122 -0.09173056 0.61475778

C 3.21283555 0.62704901 1.85149958

C 3.29688994 -0.09264245 3.08897421

C 2.62663654 1.92773165 4.32166173

C 3.21885422 0.62581176 4.32185184

C 2.13308125 2.45692163 5.55671462

C 2.62263178 1.92327102 6.79564211

C 0.89258562 3.16349620 8.02965491

C 2.13905462 2.45924824 8.02985766

C 0.18533718 3.30662902 9.26342447

C 0.90460376 3.18012405 10.50225182

C 3.30332383 -0.09021849 -4.32378286

C 3.21892691 0.62761961 -3.09115074

C 3.29318644 -0.09292543 -1.85338305

C 2.63245267 1.93144527 -0.61727976

C 3.21639949 0.62659950 -0.61717257

C 2.13851248 2.46015575 0.61494435

C 2.62303730 1.92277955 1.85135145

C 0.89337946 3.16101979 3.08879679

C 2.13632860 2.45885720 3.08899031

C 0.18541691 3.30715138 4.32147799

C 0.89330184 3.15721589 5.55659579

C -1.23198131 3.13885478 6.79541529

C 0.18379367 3.30124716 6.79555623

C -1.89029192 2.84448713 8.02999308

C -1.23281299 3.14412473 9.26345237

C -2.95666791 1.89496699 10.50325801

C -1.90399596 2.85909394 10.50276231

C 3.30719904 -0.08892385 -9.26565614

C 3.22586610 0.62794500 -8.03176080

C 3.29966353 -0.09064524 -6.79783224

C 2.62824611 1.92489086 -5.55888605

C 3.21732592 0.62849317 -5.55872529

C 2.14300738 2.46241026 -4.32403508

C 2.62757589 1.92693093 -3.09117158

C 0.89604071 3.15826652 -1.85432550

C 2.13472818 2.45778415 -1.85381352

C 0.18669095 3.30754155 -0.61818584

C 0.89469790 3.16276525 0.61454003

C -1.23108133 3.13906211 1.85034886

C 0.18325820 3.30100047 1.85082042

C -1.89174393 2.84173429 3.08812996

C -1.23553476 3.14408418 4.32118610

C -2.94133936 1.87652962 5.55651408

C -1.89099133 2.83797935 5.55648671

C -3.29847011 1.24724048 6.79602276

C -2.94629224 1.87786125 8.03021342

C -3.58777207 -0.14790192 9.26519675

C -3.30106159 1.25039480 9.26456093

C -3.52351173 -0.87407072 10.50488572

C 2.65493227 1.93848669 -10.50448777

C 3.24559606 0.63894868 -10.50437383

C 2.14709982 2.46381566 -9.26592256

C 2.63353698 1.93130019 -8.03189008

C 0.90036977 3.16133963 -6.79891699

C 2.14082787 2.45978238 -6.79808222

C 0.18902263 3.30455366 -5.56068778

C 0.89793516 3.16653236 -4.32479710

C -1.23043930 3.14403379 -3.09373640

C 0.18778844 3.30650418 -3.09262593

C -1.88855029 2.83934516 -1.85636574

C -1.23297183 3.14420778 -0.61900419

C -2.94486250 1.87837409 0.61257789

C -1.89135352 2.84392532 0.61325512

C -3.29777210 1.24598510 1.84940311

C -2.94457758 1.87762525 3.08783327

C -3.59132030 -0.15070619 4.32098948

C -3.30386930 1.25050780 4.32097629

C -3.50410196 -0.86845341 5.55696786

C -3.58485424 -0.14880031 6.79637616

C -2.91594814 -2.16900712 8.03164343

C -3.50865354 -0.86580885 8.03139783

C -2.42711228 -2.69975639 9.26583532

C -2.93247005 -2.17335339 10.50523506

C 0.19510728 3.33093128 -10.50659849

C 0.90480039 3.16684469 -9.26681325

C -1.22655902 3.14758342 -8.03535968

C 0.19573529 3.31051938 -8.03394434

C -1.88274353 2.84248025 -6.80202916

C -1.22564600 3.14231908 -5.56195955

C -2.94246367 1.88087258 -4.32872958

C -1.88786284 2.84717113 -4.32743054

C -3.29763330 1.25022341 -3.09622598

C -2.93822571 1.87760579 -1.85737978

C -3.58748560 -0.14979082 -0.62130698

C -3.30056911 1.25013416 -0.62079489

C -3.50778441 -0.86805561 0.61179740

C -3.58384921 -0.14862630 1.84918376

C -2.91693523 -2.16824070 3.08794586

C -3.50756282 -0.86850711 3.08770349

C -2.43117160 -2.70284257 4.32151388

C -2.91472732 -2.16477560 5.55724922

C -1.18456781 -3.39914926 6.79686149

C -2.42537531 -2.69815706 6.79692055

C -0.47788799 -3.54685706 8.03135231

C -1.18433593 -3.40201730 9.26562201

C 0.94608831 -3.40249074 10.50386988

C -0.47213089 -3.56438085 10.50448501

C -1.22314945 3.16885971 -10.50812152

C -2.93375662 1.88403331 -9.27184786

C -1.88115927 2.84820569 -9.27017065

C -3.29202040 1.25547605 -8.03887837

C -2.93346165 1.87974478 -6.80349097

C -3.57802866 -0.14595704 -5.56599007

C -3.29199860 1.24901258 -5.56500984

C -3.50514299 -0.86614664 -4.33058127

C -3.58421562 -0.14833530 -3.09696884

C -2.91183899 -2.16405732 -1.85869501

C -3.50054497 -0.86784499 -1.85878815

C -2.42817662 -2.70178533 -0.62117532

C -2.91671477 -2.16912588 0.61195036

C -1.18539212 -3.39980330 1.84993354

C -2.42478950 -2.69932119 1.84952009

C -0.47690130 -3.54737847 3.08848082

C -1.18580732 -3.40655186 4.32166699

C 0.93928064 -3.38094973 5.55722117

C -0.47541802 -3.54330837 5.55732553

C 1.59846409 -3.08034953 6.79622680

C 0.94440899 -3.38389606 8.03089711

C 2.65452469 -2.11979761 9.26405720

C 1.60159341 -3.08357165 9.26438701

C 3.03147472 -1.49393753 10.50274391

C -3.59386438 -0.13872284 -10.51348702

C -3.30792091 1.25973640 -10.51201122

C -3.49597266 -0.86279094 -9.27470049

C -3.57942970 -0.14695800 -8.04009999

C -2.90630642 -2.16197132 -6.80601815

C -3.49566774 -0.86441956 -6.80586325

C -2.41909876 -2.69688554 -5.56622944

C -2.91364137 -2.16850160 -4.33065829

C -1.18236847 -3.40301097 -3.09617990

C -2.42503032 -2.70021275 -3.09704533

C -0.47314002 -3.54344164 -1.85726177

C -1.18424683 -3.40519597 -0.62048257

C 0.94291453 -3.38585526 0.61367799

C -0.47679211 -3.54872042 0.61301081

C 1.59881992 -3.08061356 1.85082561

C 0.94138974 -3.38450141 3.08875790

C 2.65518530 -2.12120302 4.32174580

C 1.60006819 -3.08689474 4.32173242

C 3.00701195 -1.48883663 5.55696685

C 2.64984043 -2.11834019 6.79605929

C 3.01096879 -1.49279131 8.03015265

C -2.42460808 -2.71177866 -10.51400628

C -2.90555531 -2.16238802 -9.27491397

C -1.17506250 -3.40157398 -8.03934675

C -2.42107758 -2.69656691 -8.04052906

C -0.46869731 -3.54120087 -6.80385294

C -1.17968585 -3.39808180 -5.56516885

C 0.94752111 -3.38575000 -4.32736676

C -0.47352259 -3.54891635 -4.32879483

C 1.60260883 -3.08362852 -3.09367680

C 0.94119419 -3.38104064 -1.85619024

C 2.65447852 -2.12027999 -0.61800542

C 1.60100951 -3.08581123 -0.61878407

C 3.01023454 -1.49158697 0.61476362

C 2.64851168 -2.11893420 1.85119786

C 3.00951988 -1.49109907 3.08902756

C -1.18239893 -3.41496450 -10.51274408

C 0.95237166 -3.38087516 -9.27046094

C -0.46578149 -3.54326230 -9.27249613

C 1.60748347 -3.08310964 -8.03544100

C 0.94712951 -3.37885087 -6.80203389

C 2.65427356 -2.11698729 -5.56043372

C 1.60428971 -3.07887019 -5.56191633

C 3.01566297 -1.49143192 -4.32466388

C 2.65528988 -2.11928705 -3.09251798

C 3.00773881 -1.48762979 -1.85421947

C 2.67893522 -2.13048488 -10.50639031

C 1.62589844 -3.09422691 -10.50835315

C 3.02059260 -1.48728879 -9.26658429

C 2.66324397 -2.11623686 -8.03368659

C 3.01332029 -1.48668231 -6.79872108

C -2.95470418 -2.19451919 -11.74644027

C -3.55335893 -0.87771984 -11.74615418

C -0.46415705 -3.60482614 -11.74379643

C 0.97304774 -3.44100895 -11.74144079

C 3.08478887 -1.50806499 -11.73722010

C 3.37463158 -0.09087902 -11.73625530

C 2.18964230 2.51506116 -11.73652280

C 0.93088865 3.22786207 -11.73755545

C -1.91326745 2.90370010 -11.74106842

C -2.98004320 1.92674500 -11.74285401

C -2.42023669 -2.69929205 -12.95817458

C -1.16990385 -3.40748066 -12.95687322

C 1.62083666 -3.09005029 -12.95214179

C 2.68078321 -2.11983406 -12.94999281

C 3.24320489 0.63226639 -12.94799907

C 2.64829168 1.94032118 -12.94814556

C -1.22366069 3.16208375 -12.95200527

C 0.20407091 3.32457514 -12.95028245

C -3.58282165 -0.14267426 -12.95755244

C -3.29518151 1.26513925 -12.95584634

C -0.49240906 -3.11695979 -14.20032026

C 0.89977436 -2.95882302 -14.19807742

C -3.09733651 -0.69455438 -14.20221918

C -2.51721830 -1.96994070 -14.20247937

C -2.63178852 1.58560029 -14.19950979

C -1.59839318 2.53180400 -14.19765006

C 0.71399281 2.79453635 -14.19471483

C 1.93319810 2.10395085 -14.19368452

C 2.61638107 -1.38728454 -14.19445400

C 2.89688727 -0.01448156 -14.19347542

C -1.32286688 -2.22369209 -14.98133772

C -2.50423863 0.37293326 -14.98085992

C -0.39989642 2.29940536 -14.97682828

C 2.08247777 0.89336286 -14.97457282

C 1.51180304 -1.90219254 -14.97692004

C -0.26453395 1.11549873 -15.72920654

C 0.99852408 0.39995653 -15.72798874

C 0.70823385 -1.02235322 -15.72937742

C -0.73417745 -1.18597468 -15.73150063

C -1.33537181 0.13535184 -15.73134967

C -1.20520946 -3.46000431 11.73690355

C -2.46432047 -2.74790904 11.73735698

C 1.63882428 -3.13595350 11.73526289

C 2.70605189 -2.15945742 11.73483084

C 3.27927409 0.64517062 11.73434593

C 2.68006366 1.96183114 11.73425083

C 0.18854540 3.37155535 11.73409577

C -1.24868977 3.20752831 11.73423608

C -3.35992614 1.27420797 11.73573908

C -3.64963201 -0.14298223 11.73662386

C -0.47601498 -3.55525516 12.94845514

C 0.95167751 -3.39285702 12.94769387

C 3.02386926 -1.49652151 12.94656789

C 3.31127716 -0.08856261 12.94635971

C 2.14749817 2.46806970 12.94609492

C 0.89690075 3.17584259 12.94594337

C -2.95366576 1.88746014 12.94719728

C -1.89398697 2.85790693 12.94651653

C -2.92023556 -2.17207483 12.94953580

C -3.51555420 -0.86430345 12.94920729

C 1.32881822 -2.76143618 14.19208952

C 2.36245903 -1.81546363 14.19165914

C -2.20274766 -2.33405593 14.19395276

C -0.98343715 -3.02420997 14.19341836

C -3.16695806 -0.21616852 14.19338786

C -2.88685620 1.15661632 14.19245707

C -1.17042161 2.72828214 14.19119522

C 0.22173255 2.88674265 14.19094244

C 2.82767422 0.46484320 14.19112186

C 2.24696918 1.74009603 14.19092553

C 0.13175430 -2.52833735 14.97313927

C -2.35079896 -1.12284834 14.97398673

C -1.78082897 1.67248362 14.97235971

C 1.05391981 1.99464067 14.97152799

C 2.23614520 -0.60187883 14.97199137

C -0.97557151 0.79381341 15.72440463

C 0.46685346 0.95760784 15.72382662

C 1.06833919 -0.36353981 15.72401152

C -0.00236792 -1.34385298 15.72475574

C -1.26560628 -0.62859843 15.72511235

H 5.64984437 2.47250729 -0.75488858

H 5.10183451 3.86131102 -1.51123013

N 5.01262698 3.26781473 -0.68466554

H 5.21814392 3.80792553 0.15765667

Table S24. The coordinate of the structures for perfect nanotube adsorbed with H_2_O for physical adsorption.

C 3.38216935 -0.54786138 10.51018638

C 3.36638734 -0.54236134 5.56394804

C 3.28926670 0.17718463 6.80326741

C 3.36734904 -0.54061388 8.03742396

C 2.71067018 1.48116050 9.27085763

C 3.29003127 0.17663639 9.27134141

C 2.23552058 2.03577225 10.50958884

C 3.38053901 -0.53783068 0.62017196

C 3.29760369 0.18047487 1.85755115

C 3.37476203 -0.53997406 3.09550317

C 2.71793953 1.48493992 4.32844228

C 3.29988702 0.17840419 4.32872094

C 2.22725024 2.01767036 5.56331308

C 2.71023468 1.47927524 6.80289138

C 0.99100402 2.73589279 8.03539133

C 2.23011572 2.01896195 8.03671878

C 0.28395123 2.88579722 9.26855191

C 1.00024513 2.75105950 10.50832054

C 3.38707262 -0.53458269 -4.32208553

C 3.30817850 0.18303031 -3.08841745

C 3.37887264 -0.53795269 -1.85018443

C 2.72451071 1.48644483 -0.61268517

C 3.30707455 0.18153235 -0.61265975

C 2.23886802 2.02218278 0.61996986

C 2.71783137 1.48068871 1.85741157

C 0.99767076 2.73425761 3.09391780

C 2.23398095 2.02054851 3.09500127

C 0.28990137 2.88768677 4.32591374

C 0.99453830 2.73021680 5.56203259

C -1.13215556 2.73515532 6.79881007

C 0.28535641 2.88184442 6.80039425

C -1.79505995 2.44731891 8.03251499

C -1.13582575 2.73870724 9.26698119

C -2.87363830 1.50639659 10.50420193

C -1.81135919 2.45983353 10.50545221

C 3.38422275 -0.53299690 -9.26654175

C 3.31028239 0.18411386 -8.03205457

C 3.38105369 -0.53463667 -6.79759554

C 2.72510204 1.48508277 -5.55750445

C 3.30515254 0.18461294 -5.55765527

C 2.24511495 2.02587930 -4.32172011

C 2.72638661 1.48674524 -3.08847053

C 1.00416756 2.73178786 -1.85134607

C 2.23736220 2.02064506 -1.85015578

C 0.29570640 2.89064874 -0.61444443

C 1.00121598 2.73631044 0.61960021

C -1.12582248 2.73557463 1.85356905

C 0.29018033 2.88196710 1.85531685

C -1.79125158 2.44622114 3.09064978

C -1.13276619 2.74074573 4.32434044

C -2.85336728 1.49158675 5.55774073

C -1.79297498 2.44193899 5.55896623

C -3.21760154 0.86485210 6.79641263

C -2.86070110 1.49135061 8.03125539

C -3.52142373 -0.52964106 9.26388942

C -3.22257959 0.86606328 9.26466357

C -3.46543219 -1.25750682 10.50284134

C 2.74499725 1.49899264 -10.50510728

C 3.32568092 0.19505102 -10.50557085

C 2.24341483 2.02801907 -9.26547976

C 2.72740050 1.49176062 -8.03170150

C 1.00626014 2.73581574 -6.79697992

C 2.24059352 2.02355197 -6.79679667

C 0.29759906 2.88576718 -5.55803308

C 1.00602197 2.74059757 -4.32229499

C -1.12267627 2.74015889 -3.09062795

C 0.29735651 2.88767975 -3.08987111

C -1.78446155 2.44276266 -1.85355663

C -1.12646084 2.73911623 -0.61592371

C -2.85320978 1.49450292 0.61463726

C -1.78839185 2.44753431 0.61610497

C -3.21358835 0.86575045 1.85089936

C -2.85475863 1.49376595 3.08937624

C -3.52056368 -0.52910433 4.32103892

C -3.22113340 0.86962485 4.32177044

C -3.43980253 -1.24840725 5.55626142

C -3.51591872 -0.52867162 6.79569561

C -2.86561988 -2.55559653 8.02980911

C -3.44696059 -1.24735152 8.02985770

C -2.38360503 -3.09201259 9.26393131

C -2.88636884 -2.56214472 10.50278743

C 0.29717173 2.91259297 -10.50448853

C 1.00722813 2.74169106 -9.26546937

C -1.12219792 2.74340210 -8.03214814

C 0.30163472 2.89194665 -8.03150831

C -1.78013748 2.44563330 -6.79826181

C -1.11861382 2.73839970 -5.55875708

C -2.84862774 1.49607637 -4.32537629

C -1.78360448 2.45089210 -4.32416792

C -3.21035911 0.86913427 -3.09329627

C -2.84512421 1.49318644 -1.85478530

C -3.51350327 -0.52764438 -0.61953358

C -3.21446924 0.86975973 -0.61861356

C -3.43949464 -1.24653033 0.61308027

C -3.51151926 -0.52639871 1.85003406

C -2.86005429 -2.55125612 3.08774784

C -3.44144349 -1.24732874 3.08792714

C -2.37987363 -3.09054320 4.32094258

C -2.86075190 -2.54930266 5.55612510

C -1.14414856 -3.80146561 6.79631735

C -2.37794003 -3.08824689 6.79557781

C -0.44116823 -3.95802881 8.03150467

C -1.14820616 -3.80695801 9.26472281

C 0.97961436 -3.83149777 10.50616913

C -0.44022000 -3.97845683 10.50463691

C -1.12249281 2.76410328 -10.50495733

C -2.84324625 1.49636754 -9.26708736

C -1.78154828 2.45037462 -9.26613881

C -3.20610467 0.87195224 -8.03381380

C -2.84064829 1.49380933 -6.79934285

C -3.50199200 -0.52545350 -5.56173905

C -3.20458798 0.86702689 -5.56099974

C -3.43442406 -1.24592976 -4.32685987

C -3.50888591 -0.52689588 -3.09406612

C -2.85052849 -2.54673848 -1.85631849

C -3.43091811 -1.24682704 -1.85630642

C -2.37117288 -3.08773861 -0.61925306

C -2.85716671 -2.55152195 0.61297030

C -1.13723190 -3.79735154 1.85087833

C -2.37039897 -3.08592972 1.85017892

C -0.43185627 -3.95303459 3.08969901

C -1.14120378 -3.80578775 4.32173059

C 0.98214483 -3.80519894 5.55948035

C -0.43424695 -3.95156540 5.55794307

C 1.64233068 -3.51302007 6.79973581

C 0.98278491 -3.81056098 8.03312839

C 2.70316463 -2.56500509 9.26962598

C 1.64067740 -3.51818272 9.26799393

C 3.08331285 -1.94361540 10.50945480

C -3.52209610 -0.52183406 -10.50693641

C -3.22457244 0.87421444 -10.50623000

C -3.42787484 -1.24579706 -9.26820331

C -3.50479697 -0.52807816 -8.03457731

C -2.84493484 -2.54712732 -6.80071137

C -3.42528793 -1.24559913 -6.80064202

C -2.36079750 -3.08487823 -5.56157673

C -2.85159897 -2.55211565 -4.32681617

C -1.12976237 -3.79964227 -3.09298346

C -2.36660200 -3.08682404 -3.09371069

C -0.42231176 -3.94671311 -1.85439904

C -1.13317995 -3.80146939 -0.61854123

C 0.99302177 -3.80556031 0.61628600

C -0.42838160 -3.95263751 0.61500048

C 1.65081290 -3.50892371 1.85424766

C 0.98820688 -3.80637555 3.09113603

C 2.71359070 -2.56319918 4.32678965

C 1.64830049 -3.51761522 4.32523485

C 3.06857881 -1.93474559 5.56307421

C 2.70336631 -2.56172722 6.80140273

C 3.06776373 -1.94048363 8.03656599

C -2.37204361 -3.10395399 -10.50720806

C -2.84685130 -2.54952400 -9.26835108

C -1.12455521 -3.80179671 -8.03426473

C -2.36460447 -3.08641871 -8.03464625

C -0.41816330 -3.94690435 -6.79961887

C -1.12720120 -3.79606172 -5.56096490

C 1.00064753 -3.80502636 -4.32429808

C -0.42203539 -3.95281217 -4.32518775

C 1.65904333 -3.51028765 -3.09061797

C 0.99375636 -3.80013441 -1.85327916

C 2.72059719 -2.55935366 -0.61434467

C 1.65592092 -3.51264556 -0.61550865

C 3.08150853 -1.93526955 0.61948734

C 2.71153286 -2.55936418 1.85563725

C 3.07612833 -1.93596125 3.09471609

C -1.13577651 -3.81758284 -10.50699104

C 1.00150596 -3.80300526 -9.26748261

C -0.41818800 -3.95117019 -9.26788138

C 1.66138033 -3.51128315 -8.03334155

C 0.99928398 -3.79938042 -6.79901030

C 2.72055490 -2.55530721 -5.55875258

C 1.66037002 -3.50593320 -5.55935847

C 3.08834830 -1.93346746 -4.32258097

C 2.72241803 -2.55769582 -3.08970672

C 3.08099856 -1.93015151 -1.85078179

C 2.73717772 -2.56926458 -10.50643863

C 1.67555706 -3.52350265 -10.50666849

C 3.08688008 -1.92909421 -9.26690688

C 2.72661031 -2.55477674 -8.03298599

C 3.08374072 -1.92844084 -6.79798297

C -2.90063812 -2.58383718 -11.73889252

C -3.48907722 -1.26235224 -11.73872243

C -0.42147502 -4.01520015 -11.73882705

C 1.01717309 -3.86454311 -11.73859918

C 3.14542180 -1.95037304 -11.73834692

C 3.44662862 -0.53558546 -11.73796071

C 2.28245446 2.07937150 -11.73668272

C 1.02986220 2.80288934 -11.73656750

C -1.81716433 2.50477916 -11.73681434

C -2.89274520 1.53749551 -11.73768133

C -2.37230525 -3.09401780 -12.95080541

C -1.12785781 -3.81250984 -12.95070963

C 1.66556705 -3.51972853 -12.95065868

C 2.73370998 -2.55857487 -12.95048333

C 3.31847621 0.18834294 -12.94969370

C 2.73418684 1.50109070 -12.94910972

C -1.12695548 2.75620317 -12.94861568

C 0.30213842 2.90595456 -12.94844796

C -3.51447849 -0.52775055 -12.95035312

C -3.21522234 0.87771506 -12.94974413

C -0.45019825 -3.52873112 -14.19549317

C 0.94327978 -3.38266804 -14.19533390

C -3.03525245 -1.08462279 -14.19514087

C -2.46557437 -2.36479493 -14.19540307

C -2.55064599 1.19176305 -14.19429366

C -1.50901166 2.12890138 -14.19363682

C 0.80556347 2.37150527 -14.19356757

C 2.01876715 1.67061198 -14.19373353

C 2.67303192 -1.82586380 -14.19501067

C 2.96482839 -0.45555913 -14.19464454

C -1.27457104 -2.62920280 -14.97583927

C -2.43446366 -0.02245871 -14.97519872

C -0.31359283 1.88587894 -14.97419761

C 2.15664824 0.45874255 -14.97470456

C 1.56280422 -2.33152571 -14.97553411

C -0.18945556 0.70066623 -15.72631628

C 1.06748703 -0.02553926 -15.72651031

C 0.76526776 -1.44537489 -15.72696264

C -0.67842643 -1.59673014 -15.72717739

C -1.26853897 -0.27038421 -15.72681314

C -1.17468120 -3.86782357 11.73575725

C -2.42644668 -3.14281953 11.73496515

C 1.67271322 -3.57298033 11.73892997

C 2.74921115 -2.60676198 11.74059746

C 3.34861668 0.19230135 11.74217655

C 2.76194172 1.51457189 11.74183302

C 0.28490670 2.94939474 11.73949871

C -1.15391981 2.80038284 11.73789783

C -3.28420745 0.88824990 11.73555573

C -3.58723253 -0.52620507 11.73479715

C -0.44873477 -3.97151490 12.94844776

C 0.98051885 -3.82347174 12.94996225

C 3.07084185 -1.94736855 12.95308565

C 3.37177123 -0.54231037 12.95387618

C 2.23299797 2.02524851 12.95331334

C 0.98965355 2.74556058 12.95206682

C -2.87338581 1.49635978 12.94792354

C -1.80409520 2.45622518 12.94915776

C -2.87924769 -2.56362608 12.94668418

C -3.46182219 -1.25007856 12.94667527

C 1.36164076 -3.19637122 14.19533290

C 2.40446139 -2.26060364 14.19696718

C -2.16554289 -2.73383391 14.19216525

C -0.95320460 -3.43619775 14.19290429

C -3.10891937 -0.60634040 14.19193762

C -2.81534453 0.76366084 14.19255169

C -1.08343520 2.31834418 14.19462944

C 0.31024010 2.46269786 14.19620888

C 2.89193243 0.01509866 14.19832349

C 2.32394077 1.29594287 14.19806053

C 0.16562350 -2.95171813 14.97449500

C -2.30285949 -1.52165260 14.97283877

C -1.70536640 1.26810323 14.97426122

C 1.13261960 1.56202784 14.97731876

C 2.28889388 -1.04623560 14.97770609

C -0.90980317 0.38090395 15.72645894

C 0.53416117 0.53040861 15.72803486

C 1.12242693 -0.79664837 15.72799137

C 0.04213022 -1.76624956 15.72642707

C -1.21384384 -1.03855401 15.72557449

O 0.29899078 5.10544297 -0.64794279

H 0.29668004 6.06400941 -0.80013204

O 0.84451752 6.22841066 -1.58053557
